# Adverse effects associated with acupuncture therapies: An evidence mapping from 535 systematic reviews

**DOI:** 10.1186/s13020-023-00743-7

**Published:** 2023-04-10

**Authors:** Meng Xu, Chaoqun Yang, Tao Nian, Chen Tian, Liying Zhou, Yanan Wu, Yanfei Li, Xinxin Deng, Xiuxia Li, Kehu Yang

**Affiliations:** 1 Health Technology Assessment Center/Evidence-Based Social Science Research Center, School of Public Health, Lanzhou University, 199 Donggang West Road, Lanzhou, 730000 China; 2 Evidence-Based Medicine Center, School of Basic Medical Sciences, Lanzhou University, 199 Donggang West Road, Lanzhou, 730000 China; 3 Key Laboratory of Evidence Based Medicine and Knowledge Translation of Gansu Province, Lanzhou, 730000 China

**Keywords:** Acupuncture, Adverse events, Evidence mapping, Systematic review

## Abstract

**Background and Objective:**

Considering that physicians and patients widely use acupuncture, it is necessary to explore its adverse effects during treatment. Herein, an evidence map was generated based on published studies to identify acupuncture-induced adverse effects and assess their severity, with the overarching goal of providing references for safe and effective implementation.

**Methods:**

A comprehensive literature search was performed in four public databases (PubMed, Embase, Web of science, and the Cochrane Library) to identify relevant studies published up to 15^th^ June 2022. In addition, relevant studies were explored in the Epistemonikos database and reference lists were retrieved as a supplement. A MeaSurement Tool to Assess systematic Reviews, Version 2 (AMSTAR-2) quality assessment tool was applied to determine the methodological quality of included systematic reviews (SRs) and/or meta-analysis (MAs), whereas Microsoft Excel 2019 tool was used for data extraction and coding. Heatmaps were generated to display disease type, countries of origin for the first authors, and the sample sizes of original studies. Moreover, bubble charts comprehensively presented intervention categories, adverse reaction types, and evidence levels.

**Results:**

A total of 535 SRs involving 33 adverse reactions were included. Among them, 22 studies were rated as high quality, 28 as moderate, 106 as low, and the rest were of critically-low quality. Numerous adverse effects were described in the studies, including syncope (86 SRs), organ or tissue injury (233 SRs), systemic reactions (113 SRs), infection (19 SRs), and other adverse events (373 SRs). Importantly, these adverse reactions were mainly associated with 19 acupuncture techniques, including electroacupuncture (n = 67), manual acupuncture (n = 47) and acupoint catgut embedding (n = 41). Furthermore, the 535 SRs described 23 diseases, among which symptoms, signs or clinical findings (83 SRs), mental, behavioral or neurodevelopmental disorders (67 SRs), and diseases of the nervous system (66 SRs) had the highest incidence.

**Conclusion:**

This evidence mapping explores the adverse effects of acupuncture, showing that there are multiple types of adverse reactions to acupuncture, with milder symptoms. The methodological assessment revealed that most of the included studies were of low- or critically low-quality. Therefore, there is a need for future randomized controlled trials and SRs to comprehensively analyze acupuncture-related adverse events in order to provide reliable and credible evidence.

**Supplementary Information:**

The online version contains supplementary material available at 10.1186/s13020-023-00743-7.

## Introduction

Acupuncture, a traditional Chinese medicine technique based on a meridional theory, stimulates acupoints along the meridian channels to resolve a clinical problem. Notably, the vital energy is referred to as “*Qi*” flows [1, 2]. Acupuncture has been practiced for over 3000 years and is currently gaining wide popularity in complementary and alternative medicine [3, 4]. To date, numerous systematic reviews (SRs) and meta-analyses (MAs) have synthesized and analyzed acupuncture-related data using strict inclusion and exclusion criteria. Acupuncture is mainly recommended for diseases involving the digestive system, circulatory system, and neurodevelopmental disorders, with studies showing that its efficacy is superior to conventional treatment [5–7].

However, the safety of acupuncture is marred with uncertainties and there are many debates about acupuncture-related adverse events. For instance, a previous systematic review identified 202 acupuncture-related adverse events reported in 98 relevant papers from 22 countries [8]. In addition, 652 acupuncturists reported 6733 adverse reactions [9]. In 2014, Cheuk et al*.* [10] conducted a systematic analysis of acupuncture treatment for epilepsy and found that patients were prone to dizziness, nausea, malaise, anorexia, and sleepiness during the treatment. However, these adverse events disappeared quickly and were not statistically different after analysis [10]. On the contrary, Quan et al*.* [11] analyzed the effect of acupuncture in infertility treatment using 27 randomized controlled trials. The study found that although acupuncture was effective in treating infertility, significant adverse reactions occurred in the treatment process [11]. It is also worth noting that the values and preferences of patients are rarely taken into account in acupuncture guidelines, which further limits the effectiveness of acupuncture recommendations and may be a factor in the occurrence of potential adverse reactions [12].

Considering the growing number of SRs involving acupuncture application for conditions or symptoms and the ensuing need for critical evaluation, this study constructed an evidence map to systematically analyze acupuncture-related adverse events, with the overarching goal of providing decision-makers with high-quality evidence [13]. Notably, an evidence map does not seek to critically analyze or synthesize evidence for the effectiveness of a particular clinical indication, but rather it helps identify gaps in evidence and inform future research priorities [14]. This study presents the current status of acupuncture-related adverse effects from the following aspects: (1) the distribution and quality of SRs and/or MAs that have reported the outcome of adverse reactions caused by acupuncture; (2) the main adverse reactions, types of needles used, and diseases associated with acupuncture; (3) whether the existence of adverse reactions is associated with the acupuncture therapist and the acupuncture techniques; and (4) whether there is a significant difference between acupuncture-related adverse effects and other interventions, such as exercise and drugs.

## Methods

This evidence mapping was in accordance with the Preferred Reporting Items for Systematic Reviews and Meta-Analyses for Scoping Reviews (PRISMA-ScR) [15] and was conducted following the methodology proposed by Global Evidence Mapping [16, 17].

### Inclusion and exclusion criteria

Studies were considered eligible if they met the following criteria: (1) Participants were healthy or unhealthy; (2) Included any acupuncture technique, such as electroacupuncture, fire needle, dry needle, auricular acupuncture, acupoint catgut embedding, and acupoint injection [18]; (3) Studies that reported adverse reactions associated with acupuncture; (4) For SRs of the same topic, we choose the updated system reviews; and (5) English language based SRs and/or MAs that were conducted according to the PRISMA guidelines [19].

Studies were excluded if they were only abstracts, online publications, conference proceedings, protocol, news, non-English language, and no full-text was available. In addition, we excluded studies describing research on acupoints of non-acupuncture treatment (no penetration into the skin), such as massage, moxibustion, and acupressure, and SRs that included animal studies.

### Literature search

Two reviewers (MX and YNW) performed a systematic search in four English-language databases (PubMed, Embase, Web of science, and the Cochrane Library) to identify relevant acupuncture-related SRs published up to 15th June 2022. The following Medical Subject Headings (MeSH) and arbitrary terms were used: (“acupuncture*” OR “electroacupuncture*” OR “electro-acupuncture” OR “needl*” OR “acupoin*” OR “laser”) AND (“meta-analysis” OR “meta analysis” OR “meta analyses” OR “meta-analyses” OR “metaanalysis” OR “metanalysis” OR “met-analysis” OR “metaanalyses” OR “metanalyses” OR “met-analyses” OR “data pooling” OR “systematic review” OR “systematic reviews”). Details of the retrieval strategy are presented in Additional file 1: Appendix text S1. We also searched the Epistemonikos database, a comprehensive evidence-based medicine database containing clinical evidence for acupuncture therapy, and reviewed the references of eligible articles.

### Study selection and data extraction

After removing duplicated studies using Endnote X9, two reviewers (MX and CT) independently screened the remaining studies using Rayyan [20] following the inclusion/exclusion criteria. Any disagreements were resolved by discussion or consensus with a third reviewer (XXL).

Next, two reviewers (MX and CQY) independently extracted data from eligible studies using a standard protocol and a data collection form in Microsoft Excel 2019, and discrepancies were resolved by a third reviewer (XXL). The following information or data were obtained from each eligible SR/MA: the name of the first author, country, and year of publication; the characteristic of participants consisting of sample size, disease types; intervention details including the name of treatment; and the type of adverse events that occurred in each study. However, since some studies did not distinguish the acupuncture technique used, multiple acupuncture types are simply represented as "acupuncture."

### Quality assessment

The methodological quality of each included systematic review was assessed using A MeaSurement Tool to Assess systematic Reviews, Version 2 (AMSTAR-2), a reliable and valid tool for quality assessment of SRs and/or MAs of both interventional and observational research [21, 22]. Notably, the AMSTAR-2 quality assessment tool consists of 16 items. Seven of the items were considered critical, including registration of the protocol before starting the review; conduct of an adequate literature search; justifying the exclusion of individual studies; satisfactory assessment of the risk of bias in the studies included in the review; use of appropriate statistical methods in performing a meta-analysis; accounting for risk of bias when interpreting the results; and evaluation of the presence and effect of publication bias. The results were divided into four grades: High indicated none or one weakness in a non-critical domain; Moderate referred to more than one weakness in non-critical domains (if multiple non-critical weaknesses, then it was considered low); Low indicated with or without one defect in a non-critical domain; and Critically low indicated more than one critical flaw with or without non-critical weaknesses. Before evaluating the methodological quality, we conducted two rounds of calibrations on the AMSTAR-2 tool and three pilot assessment tests were done by all reviewers until they understood and agreed on the assessment items. Two reviewers (MX and YNW) independently applied the AMSTAR-2 tool to rate the methodological quality of the SRs, and any disagreements were resolved by consensus with a third reviewer (XXL).

### Data analyses

We generated a heatmap using GraphPad prism version 9.3 and a bubble plot using Microsoft Excel 2019 to visually display the information associated with adverse effects in the process of acupuncture therapy. The heatmap indicated the countries in which the eligible acupuncture SRs were conducted and the targeted disease types. Specifically, the Y-axis showed the disease type (according to International Classification of Diseases 11th Revision, ICD-11) [23], the X-axis displayed the country of the first author, and color represented the number of studies. On the other hand, the bubble plot summarized the quantity and quality of SRs of each identified acupuncture-induced adverse effect and the current situation of adverse effects caused by different acupuncture types. The X-axis on the bubble plot depicted the type of adverse effects in the acupuncture process, whereas the Y-axis illustrated the SRs and/or MAs included in each quality category, and presented the statistical effect of each adverse reaction in each quality. The number of bubbles showed the number of adverse effects reported in the included studies, the size represented the number of original studies included in each eligible systematic review, and the color indicated the different types of acupuncture.

## Results

### Literature screening process

**Fig. 1:**
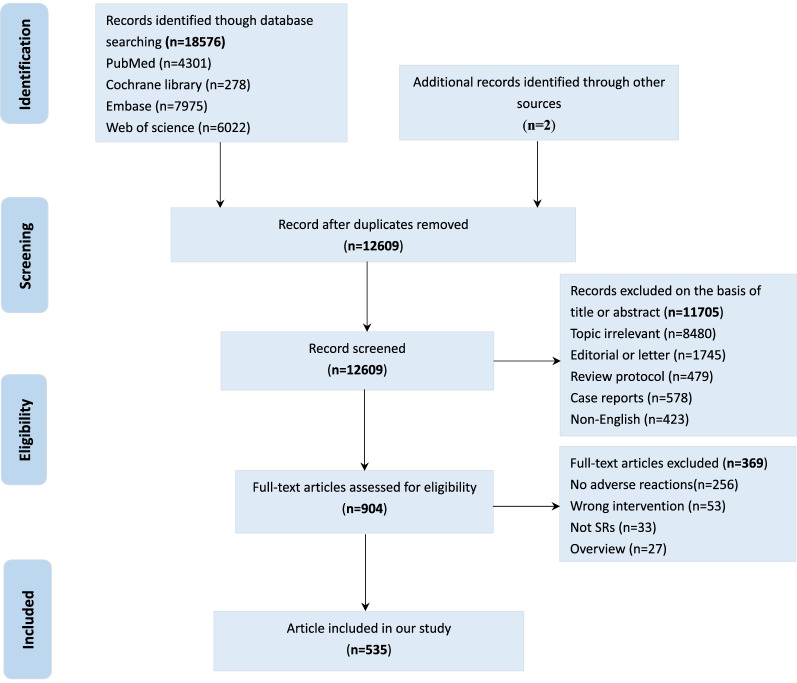
Preferred Reporting Items for Systematic Reviews and Meta-Analyses (PRISMA) Flow Diagram of Study Selection Process

A total of 18576 studies were retrieved after screening the databases, of which 5967 duplicate articles were excluded. After reviewing the title and abstract, 11705 studies were excluded (irrelevant topics, editorial or letters, review protocols, case reports, and non-English studies). The remaining 904 studies were subjected to full-text screening to exclude the studies without adverse effects reactions, wrong intervention, and non-SR. Finally, 535 studies were included in the analysis (Fig. 1 and Additional file 1: appendix table S1). Additional file 1: Appendix text S2 presents the list of eligible studies.

### Study characteristics

**Fig. 2:**
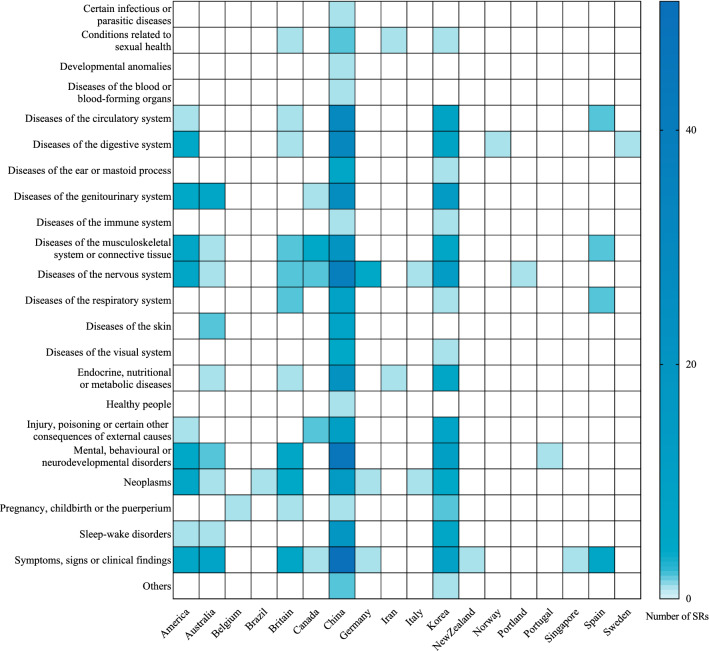
Heatmap for Characteristic of Included Studies

The heatmap illustrated in Fig. 2 shows the country distribution of the first authors, disease types, and the number of included studies. Results indicated that the studies were conducted in 18 countries, with China having the largest number (n = 336), followed by South Korea (n = 90) and the United States (n = 30). The included SRs described 23 disease types classified by ICD-11, of which the most widespread were symptoms, signs or clinical findings (83 SRs), mental, behavioral or neurodevelopmental disorders (67 SRs), and diseases of the nervous system (66 SRs). In addition, the acupuncture techniques involved were electroacupuncture (n = 67), manual acupuncture (n = 47), acupoint catgut embedding (n = 41), dry needling therapy (n = 39), auricular acupuncture (n = 22), acupoint injection (n = 14), scalp acupuncture (n = 9), bee venom acupuncture (n = 9), fire needling therapy (n = 9), battlefield acupuncture (n = 5), ear acupuncture (n = 5), filiform acupuncture (n = 2), wrist-ankle acupuncture (n = 3), laser acupuncture (n = 2), warm needling therapy (n = 2), body acupuncture (n = 1), abdominal acupuncture (n = 1), and eye-acupuncture (n = 1).

### Quality assessment

**Fig. 3:**
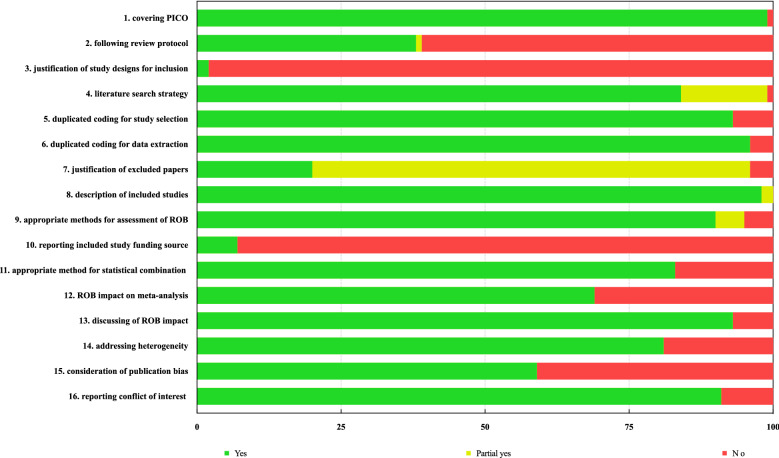
Evaluation of the Methodological Quality with AMSTAR-2

Results obtained from the AMSTAR-2 [22] quality assessment tool indicated that the methodological quality of most SRs ranged from low to critically low (Additional file 1: Appendix table S2 provides details of the evaluation result of methodological quality, with the single items summarized in Fig. 3). Specifically, 106 SRs exhibited low methodological quality, whereas 379 SRs had critically low quality. Among the remaining 50 SRs, 22 were free of critical flaws and "provided an accurate and comprehensive summary of the results that address the question of interest." With regard to critical items in AMSTAR-2, such as protocol registration, literature search strategy, excluded studies, risk of bias assessment and influence on results, methods for meta-analyses, and assessment and influence of publication bias, only 38% and 20% of the studies reported protocol registration (domain 2) and excluded studies (domain 7), respectively. In other domains, almost all included studies (over 99%) reported PICO (i.e., population, intervention, comparison and outcomes) elements (domain 1). However, the vast majority did not explain the study designs (domain 3, over 98%) nor indicate the source of funding (item 10, over 93%).

### Evidence map of acupuncture-related adverse events

**Fig. 4:**
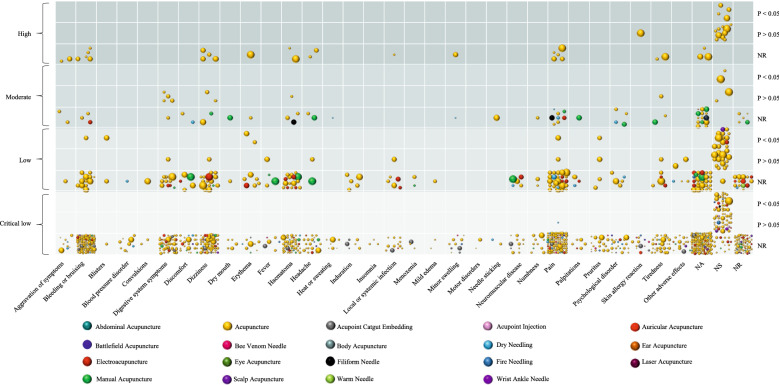
Distribution of Acupuncture-related Adverse Effect

Figure 4 shows that 119 SRs were statistically analyzed for adverse events, 176 SRs reported the occurrence of no adverse events, 120 SRs reported no specific adverse reaction type, 89 SRs showed that none of the included original studies reported adverse reactions, and 260 SRs reported different adverse reactions. Acupuncture-related adverse events were classified as syncope (86 SRs), organ or tissue injury (233 SRs), systemic reactions (113 SRs), infection (19 SRs), and other adverse events (373 SRs). The most common adverse reactions are pain (144 SRs), bleeding/bruising (120 SRs), dizziness (86 SRs), haematoma (70 SRs), digestive system symptoms (46 SRs). Notably, only a few included studies reported adverse effects due to acupuncture therapists.

### The effect size of adverse events caused by acupuncture

#### Evidence of a positive effect

This study found 50 SRs with statistically significant acupuncture-related adverse effects, ranging from high- to critically low-quality. Most studies described general acupuncture or the specific types of acupuncture could not be distinguished, except for one slap acupuncture and one electroacupuncture in the low-quality category, and two fire acupuncture, two electroacupuncture, one auricular acupuncture, and one filiform needle in the critically low-quality category.

#### Evidence of a negative effect

Among the acupuncture-related adverse events, 76 were found to have no statistical difference when acupuncture alone or combined with other interventions was compared with other interventions. There was no significant difference in the adverse effects described in 44 studies that were rated as high quality (n = 11), moderate quality (n = 10) and low quality (n = 23) by the AMSTAR-2 tool, all of which used traditional acupuncture or the specific acupuncture type was not described. The remaining 32 SRs were of critically low quality, including dry needling (n = 1), fire needle (n = 1), scalp acupuncture (n = 1), wrist ankle needle (n = 1), electroacupuncture (n = 2), acupoint injection (n = 2), and the rest acupuncture.

#### Evidence of unclear effects

Moreover, results showed that more than 90 percent of the adverse reactions were only qualitatively described, and no statistical difference was reported. Forty-three adverse effects were described in a high-quality study that compared acupuncture with other interventions. In moderate quality studies, there were several types of acupuncture techniques, including electroacupuncture, filiform needle, manual acupuncture, auricular acupuncture, and dry needle, compared to other interventions. Low-quality studies compared acupoint catgut embedding, acupoint injection, ear acupuncture, scalp acupuncture, warm acupuncture, and fire needle. In moderate- and low-qualities, manual acupuncture was the most common method compared to other interventions. Importantly, critically low-quality studies explored all acupuncture types, with acupoint catgut embedding being the most frequently used among the minority acupuncture types.

### Acupuncture-related adverse effects

#### Syncope

Acupuncture-related syncope and fainting, a reflex induced by vagal excitation and a common adverse event during acupuncture [24], were reported in 86 SRs (7.04%). This may be attributed to excessive stress and fear of acupuncture in patients, especially those new to acupuncture, frail, seriously ill, with excessive blood loss, with a history of dizziness from acupuncture, or in an improper posture. For doctors, most incidents of needle sickness were caused by the intensity of acupuncture. However, most studies demonstrated that acupuncture disease could be improved by rest and symptomatic treatment, with only a few severe cases.

#### Organ or tissue injury

A total of 233 SRs reported tissue or organ injury caused by acupuncture, mainly including pain (11.79%, 144 SRs), bleeding or bruising (9.83%, 120 SRs), haematoma (5.73%, 70 SRs), headache (2.21%, 27 SRs), pruritus (1.88%, 23 SRs), erythema (1.56%, 19 SRs), neuromuscular disease (1.31%, 16 SRs), aggravation of symptoms (1.15%, 14 SRs), skin allergy reaction (1.15%, 14 SRs), minor swelling (1.06%, 13 SRs), numbness (1.06%, 13 SRs), induration (0.90%, 11 SRs), palpitations (0.82%, 10 SRs), menoxenia (0.41%, 5 SRs), mild edema (0.25%, 3 SRs), blisters (0.25%, 3 SRs), and motor disorders (0.16%, 2 SRs).

Pain was the most frequently reported tissue or organ injury, with 144 studies. It was evident that improper acupuncture operation could lead to local chronic pain, chronic swelling pain, and other adverse reactions.

Local bleeding during acupuncture was reported in 120 studies. It is worth noting that bleeding during acupuncture is relatively common because many blood vessels are under the skin. Although acupuncturists avoid large and thick blood vessels when performing acupuncture, the tiny capillaries are difficult to see with the naked eye, and thus the needle may break the small blood vessel resulting in bleeding. However, patients only need to wipe the blood with a cotton swab in time and press it to relieve pain.

Heamatoma, a swelling and pain caused by bleeding under the skin at the acupuncture site, was reported in 70 studies. Given that the needle tip is bent with a hook, it can damage the flesh or stab a blood vessel. After withdrawing the needle, the acupuncture site was swollen and painful, and the skin was blue-purple. Generally, it is not necessary to deal with a small amount of subcutaneous hemorrhage and local bruising because it can subside on its own. However, cold compresses can be applied to stop the bleeding in instances where the local swelling and pain are severe, the bruising area is large, and the activity function is affected. In addition, hot compresses or light massage can be applied in the local area to promote dissipation and absorption of local blood stasis. To avoid heamatoma adverse effects, acupuncturists or professionals should carefully check the needles, familiarize themselves with the anatomical parts of the human body, avoid puncturing blood vessels, and press the needle hole with a sterile dry cotton ball immediately after the needle is withdrawn.

Headache, primarily due to subarachnoid hemorrhage, was reported in 27 SRs. Results revealed that the acupoints associated with headaches were Fengchi (GB20), Yamen (GV16), Anmian (EX-HN14), and Yiming (EXHN13).

Furthermore, 23 SRs reported observation of pruritus events during the interventions. The remaining 80 SRs reported mild and transient adverse events, including erythema (1.56%, 19 SRs), neuromuscular disease (1.31%, 16 SRs), aggravation of symptoms (1.15%, 14 SRs), skin allergy reactions (1.15%, 14 SRs), swelling (1.06%, 13 SRs), numbness (1.06%, 13 SRs), induration (0.90%, 11 SRs), palpitations (0.82%, 10 SRs), blood pressure disorders (0.82%, 10 SRs), menoxenia (0.41%, 5 SRs), edema (0.25%, 3 SRs), blisters (0.25%, 3 SRs), insomnia (0.25%, 3 SRs), motor disorders (0.16%, 2 SRs), and other adverse events (1.72%, 21 SRs). It should be noted that no serious adverse events were associated with the acupuncture treatments.

#### Systemic reactions

Acupuncture-related systemic reactions, including digestive system symptoms, tiredness, discomfort, psychological disorder, fever, and heat/sweating, were reported in 113SRs.

Among them, digestive system symptoms were reported in 46 SRs (3.77%). The common adverse effects associated with digestive symptoms included nausea/vomiting, loss of appetite, dry mouth, constipation, diarrhea, stomach, dyspepsia, and heartburn.

The reported tiredness (3.28%, 40 SRs), discomforts (2.54%, 31 SRs), and psychological disorder (2.46%, 30 SRs) adverse effects may be attributed to improvement of the viscera function after acupuncture, blood adjusting well, short fatigue and discomforts after constantly improved body function, improved metabolism, and improved immunity and physical fitness. However, results suggested that blood biochemical and related inspection diagnosis should be done before treatment for patients with severe fatigue. Moreover, the patient's complexion was generally dark after treatment, leading to a psychological disorder. Therefore, it is recommended that acupuncture treatment should first consider personal conditions and should not be carried out frequently.

Fevers and heat/sweating were reported in 10 SRs (0.82%) and 8 SRs (0.66%), respectively. Although some patients developed a fever after acupuncture, it was infrequent. Patients may suffer from mild colds and low fever after acupuncture because the body is exposed and may not be well protected during the process. On the other hand, fever after acupuncture may be due to hyperthermia during acupuncture, and is characterized by a slight local or systemic heat sensation after the treatment, which is a normal phenomenon.

#### Infection

Infection, one of the most common acupuncture adverse effects, was reported in 19 studies (1.56%). All patients recovered after appropriate treatment. Infection is mainly due to unsterilized needles, repeated use of needles, or contact with clothing at the needlepoint. Acupuncture points on the head are most commonly infected because the hair makes sterility standards challenging to enforce. One study reported that acupuncture techniques for relieving toothache could cause facial abscesses [25]. Other infections may be associated with the patient's blood glucose level [26]. However, acupuncture-related infection rates have been dwindling in recent years due to the popularity of health consciousness and the concept of disinfection.

#### Other adverse events

A total of 373 articles reported adverse reactions that were neither due to trauma nor infection, such as needle sticking, broken needle, and bent needle. These effects may be associated with a poor sense of responsibility of the doctors, improper twisting, improper patient positioning during the treatment process, and poor needle quality [26]. In addition, 176 studies did not report any acupuncture-related adverse events, 120 studies did not specify which adverse events were involved, and 89 studies stated that no adverse events were reported in the original study**.**

## Discussion

This evidence map provides a broad overview of adverse effects associated with acupuncture based on 535 published SRs.

Considering that acupuncture therapy is widely used worldwide, acupuncture-related adverse events are increasing with each passing day. Consequently, the imperfection of acupuncture therapy has become a potential restriction factor hindering its further development. It is worth mentioning that acupuncture-related accidents cause considerable losses to patients, doctors, hospitals, and other medical systems.

### Key findings

Among the 535 included SRs, 53 studies reported that acupuncture adverse events were associated with the practitioners. This may be attributed to the fact that most doctors, especially grassroots doctors, use acupuncture points based on their medical knowledge and experience.

Adverse events in medical practice have always been the focus of public and medical attention. Herein, we found that severe needle-related adverse reactions were rare, with the incidence of adverse events ranging from 6.71% to 8.6%, and the incidence of serious adverse events was about 0.001% [27, 28]. A comparison of the incidence of adverse events between acupuncture and conventional prescription drugs in primary care showed that acupuncture was a safe treatment [29]. Some adverse reactions, such as syncope, can be reduced by adequately preparing and positioning the patient, preferably in the supine, lateral or prone position. In addition, the patient should not feel hungry or fatigued. Care should be taken to avoid damage to nerves and blood vessels when acupuncture is directed at the umbilical points of the legs, arms, and face. In addition, doctors should pay more attention to aseptic operations to prevent infection. They should also reduce the force of acupuncture on very shallow acupoints to avoid damage to peripheral nerves, capillaries, and muscle fibers.

This study applied the latest high-quality methodological standards and tools to ensure high quality and transparency. However, most of the included studies were of low- to critically low-quality, which raises questions about whether the AMSTAR-2 tool was flawed. Although most of the included SRs were of low- or critically low-quality due to failure to report the reason of study design for inclusion (domain 3) and to provide a list and reasons for excluded studies (domain 7), some critical domains of the AMSTAR-2 tool, such as domain 11 (“If meta-analysis was performed, were appropriate statistical analysis methods used?”) and domain 15 (“If quantitative synthesis was performed, did the authors investigate and discuss publication bias?”) [16, 17], had an impact on the quality of SRs that did not perform a meta-analysis. However, despite these issues, we believe that using another tool would most likely have given the same results because many of the signaling questions in AMSTAR-2 overlap with similar tools such as Risk of Bias in Systematic Review [30] and the original AMSTAR tool [31], which is embedded in AMSTAR-2.

### Clinical value

Acupuncture is the insertion of one or several needles into the skin at specific sites or acupuncture points for therapeutic purposes [32]. This study has shown that many types and large numbers of adverse reactions are associated with acupuncture. In addition, these conditions may be caused by the patient's constitution (such as allergies to metal products or other complications), poor needle quality, or improper operation by the acupuncturist. Therefore, to avoid these adverse reactions, the needle quality should be strictly controlled, acupuncturists or other professional operators should receive strict and standardized training, and a pre-test (similar to a skin test before an antibiotic injection) should be performed to understand the patient's constitution before the acupuncture process.

## Strengths and limitations

The study conducted a systematic and comprehensive literature search in four public databases to identify all published SRs and/or MAs regarding the adverse effects of acupuncture. Notably, the entire evaluation process, from literature screening and data extraction to quality assessment, was independently completed by two evaluators. We believe that our findings are reliable because the AMSTAR-2 quality assessment method was applied to determine the methodological quality of the included studies.

However, the study had some limitations. First, we only searched four English databases, which may result in the missing of eligible studies. Fortunately, we conducted a supplementary search of Epistemonikos, the most comprehensive acupuncture research database, to minimize the omission of relevant studies. Second, the results may be inconclusive because many included studies were not detailed. Specifically, most of the included SRs only provided a brief description of the adverse reactions and rarely analyzed the causes of adverse reactions [33]. Finally, because this study primarily analyzed the distribution of evidence, we did not consider the overlap of randomized controlled trials in the included SRs, which may have biased the study results.

## Conclusion

This evidence map provides a comprehensive summary of acupuncture-related adverse effects as described in published SRs and/or MAs. Unfortunately, most of the included studies were of low- or critically low-quality and only qualitatively described the acupuncture-related adverse effects or did not analyze individual adverse effects. This may be attributed to the fact that most primary studies did not provide adequate details of acupuncture-related adverse effects, thereby limiting the extent of conclusions drawn by the SRs. Therefore, researchers need to pay more attention to high-quality evidence, including original studies and SRs.

## Supplementary Information

**Additional file 1**: **Text S1**: Search strategies. **Text S2**: The list of eligible studies. **Table S1**: Basic characteristics of the included studies. **Table S2**: Detailed evaluation of the methodological quality with AMSTAR-2.

## Data Availability

Not applicable.

## Abbreviation

AMSTAR-2: A MeaSurement Tool to Assess systematic Reviews, Version 2
SR: Systematic review
MA: Meta-analysis
PRISMA: Preferred Reporting Items for Systematic Reviews and Meta-Analyses
PRISMA-ScR: Preferred Reporting Items for Systematic Reviews and Meta-Analyses for Scoping Reviews
PICO: Population, Intervention, Comparison and Outcomes
ICD-11: International Classification of Diseases 11th Revision
