# Supplementary material for: Adverse effects associated with acupuncture therapies: An evidence mapping from 535 systematic reviews
Source: Chin Med. 2023 Apr 10;18:38. doi: 10.1186/s13020-023-00743-7 (PMC10088157; doi:10.1186/s13020-023-00743-7)
Supplement: Supplementary file 1 — Additional file 1: Text S1: Search strategies. Text S2: The list of eligible studies. Table S1: Basic characteristics of the included studies. Table S2: Detailed evaluation of the methodological quality with AMSTAR-2. [file 13020_2023_743_MOESM1_ESM.docx]

**Adverse effects associated with acupuncture therapies: An evidence mapping from 535 systematic reviews**

Meng Xu, Chaoqun Yang, Tao Nian, Chen Tian, Liying Zhou, Yanan Wu, Yanfei Li, Xinxin Deng, Kehu Yang, Xiuxia Li

Content

[Text S1: Search strategies 3](#_Toc116682873)

[Text S2: The list of eligible studies 5](#_Toc116682874)

[Table S1: Basic characteristics of the included studies 44](#_Toc116682875)

[Table S2: Detailed evaluation of the methodological quality with AMSTAR 2 136](#_Toc116682876)

# Text S1: Search strategies

| **#** | **Pubmed** |
| --- | --- |
| 1 | "Acupuncture"[MeSH Terms] OR "Acupuncture Therapy"[MeSH Terms] OR "Acupuncture Points"[MeSH Terms] OR "Electroacupuncture"[MeSH Terms] |
| 2 | acupuncture*[Title/Abstract] OR electroacupuncture*[Title/Abstract] OR "electro-acupuncture"[Title/Abstract] OR needl*[Title/Abstract] OR acupoin*[Title/Abstract] OR laser [Title/Abstract] |
| 3 | meta-analysis"[Publication Type] OR "Meta-Analysis as Topic"[MeSH Terms] OR "meta analysis"[Title/Abstract] OR "meta analyses"[Title/Abstract] OR "meta-analysis"[Title/Abstract] OR "meta-analyses"[Title/Abstract] OR "metaanalysis"[Title/Abstract] OR "metanalysis"[Title/Abstract] OR "met-analysis"[Title/Abstract] OR "metaanalyses"[Title/Abstract] OR "metanalyses"[Title/Abstract] OR "met-analyses"[Title/Abstract] OR "data pooling"[Title/Abstract] OR "data poolings"[Title/Abstract] OR "systematic review"[Title/Abstract] OR "systematic reviews"[Title/Abstract] |
| 4 | #1 OR #2 |
| 5 | #3 AND #4 |

| **#** | **Web of Science** |
| --- | --- |
| 1 | TS= (acupuncture*  OR electroacupuncture*  OR electro-acupuncture  OR needl*  OR acupoin*  OR laser） |
| 2 | TS= ("Meta-Analysis as Topic" OR "Meta-Analysis" OR " meta analysis" OR "meta analyses" OR "meta-analysis" OR "meta-analyses" OR metaanalysis OR metanalysis OR "met-analysis" OR metaanalyses OR metanalyses OR "met-analyses" OR "data pooling" OR "data poolings" OR "clinical trial overview" OR "clinical trial overviews" OR "systematic review" OR "systematic reviews") |
| 3 | #1 AND #2 |

| **#** | **Cochrane Library** |
| --- | --- |
| 1 | MeSH descriptor: [Acupuncture] explode all trees |
| 2 | MeSH descriptor: [Acupuncture Therapy] explode all trees |
| 3 | MeSH descriptor: [Acupuncture Points] explode all trees |
| 4 | MeSH descriptor: [Electroacupuncture] explode all trees |
| 5 | (acupuncture* OR electroacupuncture* OR electro-acupuncture OR needl* OR acupoin* OR laser):ti,ab,kw |
| 6 | #2 AND #2 AND #3 AND #4 AND #5 |

| **#** | **Embase** |
| --- | --- |
| 1 | 'acupuncture'/exp OR 'acupuncture point'/exp OR 'electroacupuncture'/exp |
| 2 | acupuncture*:ti,ab OR electroacupuncture*:ti,ab OR 'electro acupuncture':ti,ab OR needl*:ti,ab OR acupoin*:ti,ab OR laser:ti,ab |
| 3 | 'meta analysis':ab,ti OR 'meta analyses':ab,ti OR 'meta-analysis':ab,ti OR 'meta-analyses':ab,ti OR metaanalysis:ab,ti OR metanalysis:ab,ti OR 'met-analysis':ab,ti OR metaanalyses:ab,ti OR metanalyses:ab,ti OR 'met-analyses':ab,ti OR 'data pooling':ab,ti OR 'data poolings:':ab,ti OR 'clinical trial overview':ab,ti OR 'clinical trial overviews':ab,ti OR 'systematic review':ab,ti OR 'systematic reviews':ab,ti OR 'systematic review'/exp OR 'systematic review (topic)'/exp OR 'meta analysis (topic)'/exp OR 'meta analysis'/exp |
| 4 | #1 OR #2 |
| 5 | #3 AND #4 |

# Text S2: The list of eligible studies

1. Manyanga T, Froese M, Zarychanski R, et al. Pain management with acupuncture in osteoarthritis: a systematic review and meta-analysis.14:312. <https://www.ncbi.nlm.nih.gov/pmc/articles/PMC4158087/pdf/12906_2013_Article_1895.pdf>.

2. Luo Y, Qian W, Dai T, et al. A New Therapy for Vitiligo Using Fire Needles: A Systematic Review of Evidence from 3618 Subjects. 2020;2020:8492097. <https://www.ncbi.nlm.nih.gov/pmc/articles/PMC7474359/pdf/ECAM2020-8492097.pdf>.

3. Lu WW, Zhang JM, Lv ZT, Chen AM. Update on the Clinical Effect of Acupuncture Therapy in Patients with Gouty Arthritis: Systematic Review and Meta-Analysis. 2016;2016:9451670. <https://www.ncbi.nlm.nih.gov/pmc/articles/PMC5099464/pdf/ECAM2016-9451670.pdf>.

4. Lu C, Wu LQ, Hao H, et al. Clinical efficacy and safety of acupuncture treatment of TIC disorder in children: A systematic review and meta-analysis of 22 randomized controlled trials.59:102734. <https://www.sciencedirect.com/science/article/pii/S0965229921000753?via%3Dihub>.

5. Liu R, Zhang K, Tong QY, Cui GW, Ma W, Shen WD. Acupuncture for post-stroke depression: a systematic review and meta-analysis.21(1):109. <https://www.ncbi.nlm.nih.gov/pmc/articles/PMC8017746/pdf/12906_2021_Article_3277.pdf>.

6. Liu FG, Tan AH, Peng CQ, Tan YX, Yao MC. Efficacy and Safety of Scalp Acupuncture for Insomnia: A Systematic Review and Meta-Analysis. 2021;2021:6621993. <https://www.ncbi.nlm.nih.gov/pmc/articles/PMC8166479/pdf/ECAM2021-6621993.pdf>.

7. Liu F, You J, Li Q, et al. Acupuncture for Chronic Pain-Related Insomnia: A Systematic Review and Meta-Analysis. 2019;2019:5381028. <https://www.ncbi.nlm.nih.gov/pmc/articles/PMC6612974/pdf/ECAM2019-5381028.pdf>.

8. Liu BP, Wang YT, Chen SD. Effect of acupuncture on clinical symptoms and laboratory indicators for chronic prostatitis/chronic pelvic pain syndrome: a systematic review and meta-analysis.48(12):1977-1991. <https://link.springer.com/content/pdf/10.1007/s11255-016-1403-z.pdf>.

9. Linde K, Allais G, Brinkhaus B, Manheimer E, Vickers A, White AR. Acupuncture for migraine prophylaxis. 2009(1). <https://www.embase.com/search/results?subaction=viewrecord&id=L355250927&from=export> <http://dx.doi.org/10.1002/14651858.CD001218.pub2>.

10. Linde K, Allais G, Brinkhaus B, et al. Acupuncture for the prevention of episodic migraine. 2016(6).

11. Linde K, Allais G, Brinkhaus B, et al. Acupuncture for the prevention of tension-type headache.4(4):Cd007587.

12. He C, Ma H. Effectiveness of trigger point dry needling for plantar heel pain: a meta-analysis of seven randomized controlled trials. 2017;10:1933-1942. <https://www.dovepress.com/getfile.php?fileID=37997>.

13. Hou XB, Chen DD. Effectiveness and safety of acupuncture on cancer pain: a meta-analysis. 2020;4. <https://www.embase.com/search/results?subaction=viewrecord&id=L2017507986&from=export> <http://dx.doi.org/10.53388/TMRIM202004019>.

14. Hoxtermann MD, Haller H, Aboudamaah S, et al. Safety of acupuncture in oncology: A systematic review and meta-analysis of randomized controlled trials %J CANCER.

15. Huang JF, Zheng XQ, Chen D, et al. Can Acupuncture Improve Chronic Spinal Pain? A Systematic Review and Meta-Analysis.11(8):1248-1265. <https://www.ncbi.nlm.nih.gov/pmc/articles/PMC8453671/pdf/10.1177_2192568220962440.pdf>.

16. Wang L, Xian J, Sun M, et al. Acupuncture for emotional symptoms in patients with functional gastrointestinal disorders: A systematic review and meta-analysis. 2022;17(1):e0263166. <https://www.ncbi.nlm.nih.gov/pmc/articles/PMC8794137/pdf/pone.0263166.pdf>.

17. Wang L, Xu M, Zheng Q, Zhang W, Li Y. The Effectiveness of Acupuncture in Management of Functional Constipation: A Systematic Review and Meta-Analysis. 2020;2020:6137450. <https://www.ncbi.nlm.nih.gov/pmc/articles/PMC7317618/pdf/ECAM2020-6137450.pdf>.

18. Wang X, Zhao NQ, Sun YX, et al. Acupuncture for ulcerative colitis: a systematic review and meta-analysis of randomized clinical trials.20(1):309. <https://www.ncbi.nlm.nih.gov/pmc/articles/PMC7560249/pdf/12906_2020_Article_3101.pdf>.

19. Wang XF, Cai W, Wang YP, Huang S, Zhang QB, Wang F. Is Electroacupuncture an Effective and Safe Treatment for Poststroke Depression? An Updated Systematic Review and Meta-Analysis.2021.

20. Fan SQ, Jin S, Tang TC, Chen M, Zheng H. Efficacy of acupuncture for migraine prophylaxis: a trial sequential meta-analysis.268(11):4128-4137. <https://link.springer.com/content/pdf/10.1007/s00415-020-10178-x.pdf>.

21. Fei Y, Fei R, Zhang J, Sun Y, Yu Q. Systematic Evaluation of Efficacy and Safety of Acupuncture Treatment for Patients with Atrial Fibrillation.7(3):461-466. <https://www.ncbi.nlm.nih.gov/pmc/articles/PMC6390133/pdf/OAMJMS-7-461.pdf>.

22. Guo JB, Xing XX, Wu JN, et al. Acupuncture for Adults with Diarrhea-Predominant Irritable Bowel Syndrome or Functional Diarrhea: A Systematic Review and Meta-Analysis.2020.

23. Xu J, Zhang FQ, Pei J, Ji J. Acupuncture for migraine without aura: a systematic review and meta-analysis.16(5):312-321. <https://www.sciencedirect.com/science/article/pii/S2095496418300761?via%3Dihub>.

24. Xu Y, Zhao WL, Li T, et al. Effects of acupoint-stimulation for the treatment of primary dysmenorrhoea compared with NSAIDs: a systematic review and meta-analysis of 19 RCTs.17.

25. Yang JJ, Li XY, Li C, et al. Comparative efficacy and safety of acupuncture and Western medicine for poststroke thalamic pain.

26. Yang MX, Sun MS, Du T, et al. The efficacy of acupuncture for stable angina pectoris: A systematic review and meta-analysis.28(13):1415-1425.

27. You JY, Li HY, Xie DY, Chen RX, Chen MR. Acupuncture for Chronic Pain-Related Depression: A Systematic Review and Meta-Analysis.2021.

28. Zhang J, He Y, Huang X, Liu Y, Yu H. The effects of acupuncture versus sham/placebo acupuncture for insomnia: A systematic review and meta-analysis of randomized controlled trials.41:101253. <https://www.sciencedirect.com/science/article/pii/S1744388120311282?via%3Dihub>.

29. Zhang QH, Yue JH, Golianu B, Sun ZR, Lu Y. Updated systematic review and meta-analysis of acupuncture for chronic knee pain.35(6):392-403.

30. Zhang SH, Liu M, Asplund K, Li L. Acupuncture for acute stroke. (2):Cd003317.

31. Zhang W, Sun JH, Gao Y, et al. System review on treating post-stroke depression with acupuncture. 2014;24(2):52-59. <https://www.embase.com/search/results?subaction=viewrecord&id=L603724431&from=export> <http://dx.doi.org/10.1016/S1003-5257(14)60026-X>.

32. Zhang Y, Jiang JS, Kuai L, et al. Efficacy and Safety of Fire Needle Therapy for Flat Warts: Evidence from 29 Randomized Controlled Trials. 2021;2021. <https://www.embase.com/search/results?subaction=viewrecord&id=L2010895293&from=export> <http://dx.doi.org/10.1155/2021/9513762>.

33. Zhang ZJ, Chen HY, Yip KC, Ng R, Wong VT. The effectiveness and safety of acupuncture therapy in depressive disorders: systematic review and meta-analysis.124(1):9-21.

34. Zheng C, Zhou T. Effect of Acupuncture on Pain, Fatigue, Sleep, Physical Function, Stiffness, Well-Being, and Safety in Fibromyalgia: A Systematic Review and Meta-Analysis. 2022;15:315-329. <https://www.dovepress.com/getfile.php?fileID=78030>.

35. Zhou L, Hu X, Yu Z, et al. Efficacy and Safety of Acupuncture in the Treatment of Poststroke Insomnia: A Systematic Review and Meta-Analysis of Twenty-Six Randomized Controlled Trials. 2022;2022:5188311. <https://www.ncbi.nlm.nih.gov/pmc/articles/PMC8957466/pdf/ECAM2022-5188311.pdf>.

36. Zhu LB, Chan WC, Lo KC, Yum TP, Li L. Wrist-ankle acupuncture for the treatment of pain symptoms: a systematic review and meta-analysis. 2014;2014:261709. <https://www.ncbi.nlm.nih.gov/pmc/articles/PMC4123534/pdf/ECAM2014-261709.pdf>.

37. Lan Y, Wu X, Tan HJ, et al. Auricular acupuncture with seed or pellet attachments for primary insomnia: a systematic review and meta-analysis.15:103. <https://www.ncbi.nlm.nih.gov/pmc/articles/PMC4425871/pdf/12906_2015_Article_606.pdf>.

38. Li P, Luo Y, Wang Q, et al. Efficacy and Safety of Acupuncture at Tianshu (ST25) for Functional Constipation: Evidence from 10 Randomized Controlled Trials. 2020;2020:2171587. <https://www.ncbi.nlm.nih.gov/pmc/articles/PMC7665928/pdf/ECAM2020-2171587.pdf>.

39. Li XB, Wang J, Xu AD, et al. Clinical effects and safety of electroacupuncture for the treatment of post-stroke depression: a systematic review and meta-analysis of randomised controlled trials.36(5):284-293.

40. Wang XY, Wang H, Guan YY, Cai RL, Shen GM. Acupuncture for functional gastrointestinal disorders: A systematic review and meta-analysis.36(11):3015-3026. <https://onlinelibrary.wiley.com/doi/pdfdirect/10.1111/jgh.15645?download=true>.

41. Wang YY, Yu SF, Xue HY, Li Y, Zhao C, Jin YH. Effectiveness and Safety of Acupuncture for the Treatment of Alzheimer's Disease: A Systematic Review and Meta-Analysis. 2020;12:98. <https://www.ncbi.nlm.nih.gov/pmc/articles/PMC7218057/pdf/fnagi-12-00098.pdf>.

42. Xiao X, Zhang J, Jin Y, Wang Y, Zhang Q. Effectiveness and Safety of Acupuncture for Perimenopausal Depression: A Systematic Review and Meta-Analysis of Randomized Controlled Trials. 2020;2020:5865697. <https://www.ncbi.nlm.nih.gov/pmc/articles/PMC6995321/pdf/ECAM2020-5865697.pdf>.

43. Mu J, Furlan AD, Lam WY, Hsu MY, Ning Z, Lao L. Acupuncture for chronic nonspecific low back pain. 2020(12). <http://dx.doi.org/10.1002/14651858.CD013814>.

44. Ou MQ, Fan WH, Sun FR, et al. A Systematic Review and Meta-analysis of the Therapeutic Effect of Acupuncture on Migraine. 2020;11:596. <https://www.ncbi.nlm.nih.gov/pmc/articles/PMC7344239/pdf/fneur-11-00596.pdf>.

45. Pei W, Zeng J, Lu L, Lin G, Ruan J. Is acupuncture an effective postherpetic neuralgia treatment? A systematic review and meta-analysis. 2019;12:2155-2165. <https://www.dovepress.com/getfile.php?fileID=51252>.

46. Quan K, Yu C, Wen X, Lin Q, Wang N, Ma H. Acupuncture as Treatment for Female Infertility: A Systematic Review and Meta-Analysis of Randomized Controlled Trials. 2022;2022:3595033. <https://www.ncbi.nlm.nih.gov/pmc/articles/PMC8865966/pdf/ECAM2022-3595033.pdf>.

47. Smith CA, Armour M, Shewamene Z, Tan HY, Norman RJ, Johnson NP. Acupuncture performed around the time of embryo transfer: a systematic review and meta-analysis.38(3):364-379. <https://www.sciencedirect.com/science/article/pii/S1472648318306758?via%3Dihub>.

48. Smith CA, Armour M, Zhu X, Li X, Lu ZY, Song J. Acupuncture for dysmenorrhoea. 2016(4). <http://dx.doi.org/10.1002/14651858.CD007854.pub3>.

49. Smith CA, Hay PP. Acupuncture for depression. (2):Cd004046.

50. Su XT, Sun N, Zhang N, et al. Effectiveness and Safety of Acupuncture for Vascular Cognitive Impairment: A Systematic Review and Meta-Analysis. 2021;13:692508. <https://www.ncbi.nlm.nih.gov/pmc/articles/PMC8377366/pdf/fnagi-13-692508.pdf>.

51. Tao B, Chao S, Zheng CH, Huang GY. Acupuncture for the treatment of functional constipation.36(5):578-587.

52. Zheng J, Lai X, Zhu W, Huang Y, Chen C, Chen J. Effects of Acupuncture Combined with Rehabilitation on Chronic Pelvic Pain Syndrome in Females: A Meta-Analysis Running Head-Acupuncture Combined with Rehabilitation on Chronic Pelvic Pain. 2022;2022:8770510. <https://www.ncbi.nlm.nih.gov/pmc/articles/PMC8941541/pdf/JHE2022-8770510.pdf>.

53. Deng KF, Li LH, Pan TZ, et al. Meta-analysis and trial sequential analysis on blood uric acid and joint function in gouty arthritis treated with fire needling therapy in comparison with western medication.32(1):49-60.

54. Jiang Y, Bai P, Chen H, et al. The Effect of Acupuncture on the Quality of Life in Patients With Migraine: A Systematic Review and Meta-Analysis. 2018;9:1190. <https://www.ncbi.nlm.nih.gov/pmc/articles/PMC6212461/pdf/fphar-09-01190.pdf>.

55. Kong DZ, Liang N, Yang GL, et al. Acupuncture for chronic hepatitis B. 2019(8). <http://dx.doi.org/10.1002/14651858.CD013107.pub2>.

56. Kwon CY, Ko SJ, Lee B, Cha JM, Yoon JY, Park JW. Acupuncture as an Add-On Treatment for Functional Dyspepsia: A Systematic Review and Meta-Analysis. 2021;8:682783. <https://www.ncbi.nlm.nih.gov/pmc/articles/PMC8350114/pdf/fmed-08-682783.pdf>.

57. Armour M, Smith CA, Wang LQ, et al. Acupuncture for Depression: A Systematic Review and Meta-Analysis.8(8).

58. Bae H, Bae H, Min BI, Cho S. Efficacy of acupuncture in reducing preoperative anxiety: a meta-analysis. 2014;2014:850367. <https://www.ncbi.nlm.nih.gov/pmc/articles/PMC4165564/pdf/ECAM2014-850367.pdf>.

59. Cao H, Pan X, Li H, Liu J. Acupuncture for treatment of insomnia: a systematic review of randomized controlled trials.15(11):1171-1186. <https://www.ncbi.nlm.nih.gov/pmc/articles/PMC3156618/pdf/acm.2009.0041.pdf>.

60. Cao HJ, Yu ML, Wang LQ, Fei YT, Xu H, Liu JP. Acupuncture for Primary Insomnia: An Updated Systematic Review of Randomized Controlled Trials.25(5):451-474. <https://www.liebertpub.com/doi/10.1089/acm.2018.0046?url_ver=Z39.88-2003&rfr_id=ori:rid:crossref.org&rfr_dat=cr_pub%3dpubmed>

https://www.liebertpub.com/doi/pdf/10.1089/acm.2018.0046?download=true.

61. Chen H, Shen FE, Tan XD, Jiang WB, Gu YH. Efficacy and Safety of Acupuncture for Essential Hypertension: A Meta-Analysis.24:2946-2969. <https://www.ncbi.nlm.nih.gov/pmc/articles/PMC5963739/pdf/medscimonit-24-2946.pdf>.

62. Abdi F, Alimoradi Z, Roozbeh N, Amjadi MA, Robatjazi M. Does acupuncture improve sexual dysfunction? A systematic review.

63. Amorim D, Amado J, Brito I, et al. Acupuncture and electroacupuncture for anxiety disorders: A systematic review of the clinical research.31:31-37. <https://www.sciencedirect.com/science/article/pii/S1744388118300689?via%3Dihub>.

64. Ang L, Song E, Jun JH, Choi TY, Lee MS. Acupuncture for treating diabetic retinopathy: A systematic review and meta-analysis of randomized controlled trials.52:102490. <https://www.sciencedirect.com/science/article/pii/S0965229920304076?via%3Dihub>.

65. Ang L, Song E, Lee HW, Kim JT, Kim E, Lee MS. Acupuncture Treatment for Nocturnal Crying in Pediatric Patients: A Systematic Review of Clinical Studies. 2021;9:647098. <https://www.ncbi.nlm.nih.gov/pmc/articles/PMC8316751/pdf/fped-09-647098.pdf>.

66. Ba J, Wu Y, Li Y, Xu D, Zhu W, Yu J. Updated meta-analysis of acupuncture for treating dry Eye. 2013;25(5):317-327. <https://www.embase.com/search/results?subaction=viewrecord&id=L370125679&from=export> <http://dx.doi.org/10.1089/acu.2013.0968>.

67. Bae K, Yoo HS, Lamoury G, Boyle F, Rosenthal DS, Oh B. Acupuncture for Aromatase Inhibitor-Induced Arthralgia: A Systematic Review.14(6):496-502.

68. Bai Y, Guo Y, Wang H, et al. Efficacy of acupuncture on fibromyalgia syndrome: A Meta-analysis. 2014;34(4):381-391. <https://www.embase.com/search/results?subaction=viewrecord&id=L600238402&from=export> <http://dx.doi.org/10.1016/s0254-6272(15)30037-6>.

69. Bai ZH, Zhang ZX, Li CR, et al. Eye Acupuncture Treatment for Stroke: A Systematic Review and Meta-Analysis. 2015;2015:871327. <https://www.ncbi.nlm.nih.gov/pmc/articles/PMC4486759/pdf/ECAM2015-871327.pdf>.

70. Bäumler PI, Park SW, Yi SH, et al. Acupuncture for the treatment of spasticity after stroke: A meta-analysis of randomized controlled trials. 2015;58(1):30-32. <https://www.embase.com/search/results?subaction=viewrecord&id=L603286376&from=export> <http://dx.doi.org/10.1016/S0415-6412(15)60012-1>.

71. Baviera AF, Olson K, de Paula JM, Toneti BF, Sawada NO. Acupuncture in adults with Chemotherapy-Induced Peripheral Neuropathy: a systematic review. 2019;27.

72. Befus D, Coeytaux RR, Goldstein KM, et al. Management of Menopause Symptoms with Acupuncture: An Umbrella Systematic Review and Meta-Analysis.24(4):314-323. <https://www.liebertpub.com/doi/10.1089/acm.2016.0408?url_ver=Z39.88-2003&rfr_id=ori%3Arid%3Acrossref.org&rfr_dat=cr_pub%3Dpubmed>

https://www.liebertpub.com/doi/pdf/10.1089/acm.2016.0408?download=true.

73. Ben-Arie E, Kao PY, Lee YC, Ho WC, Chou LW, Liu HP. The Effectiveness of Acupuncture in the Treatment of Frozen Shoulder: A Systematic Review and Meta-Analysis. 2020;2020:9790470. <https://www.ncbi.nlm.nih.gov/pmc/articles/PMC7532995/pdf/ECAM2020-9790470.pdf>.

74. Blanco-Díaz M, Ruiz-Redondo R, Escobio-Prieto I, De la Fuente-Costa M, Albornoz-Cabello M, Casaña J. A Systematic Review of the Effectiveness of Dry Needling in Subacromial Syndrome.11(2).

75. Bonomo P, Stocchi G, Caini S, et al. Acupuncture for radiation-induced toxicity in head and neck squamous cell carcinoma: a systematic review based on PICO criteria.279(4):2083-2097. <https://www.ncbi.nlm.nih.gov/pmc/articles/PMC8930866/pdf/405_2021_Article_7002.pdf>.

76. Bower WF, Diao M, Tang JL, Yeung CK. Acupuncture for nocturnal enuresis in children: a systematic review and exploration of rationale. 2005;24(3):267-272. <https://onlinelibrary.wiley.com/doi/10.1002/nau.20108>.

77. Cai YY, Zhang CS, Liu SN, et al. Electroacupuncture for Poststroke Spasticity: A Systematic Review and Meta-Analysis.98(12):2578-2589. <https://www.archives-pmr.org/article/S0003-9993(17)30257-5/fulltext>.

78. Cao H, Wang Y, Chang D, Zhou L, Liu J. Acupuncture for vascular mild cognitive impairment: a systematic review of randomised controlled trials.31(4):368-374. <https://www.ncbi.nlm.nih.gov/pmc/articles/PMC3888636/pdf/acupmed-2013-010363.pdf>.

79. Cao JP, Yuan AH, Zhang Y, Yang J, Song XG. Effect of warm needling therapy and acupuncture in the treatment of peripheral facial paralysis: A systematic review and meta-analysis.28(4):278-286.

80. Cao L, Zhang XL, Gao YS, Jiang Y. Needle acupuncture for osteoarthritis of the knee. A systematic review and updated meta-analysis.33(5):526-532.

81. Casimiro L, Barnsley L, Brosseau L, et al. Acupuncture and electroacupuncture for the treatment of rheumatoid arthritis. 2005(4). <http://dx.doi.org/10.1002/14651858.CD003788.pub2>.

82. Chai Q, Fei Y, Cao H, Wang C, Tian J, Liu J. Acupuncture for melasma in women: a systematic review of randomised controlled trials.33(4):254-261.

83. Chan YT, Wang N, Tam CW, et al. Systematic Review with Meta-Analysis: Effectiveness and Safety of Acupuncture as Adjuvant Therapy for Side Effects Management in Drug Therapy-Receiving Breast Cancer Patients.2021.

84. Chang H, Lee H, Kim H, Chung WS. The Use of Acupuncture in the Management of Patients With Humeral Fractures: A Systematic Review and Meta-analysis.44(2):146-153. <https://www.jmptonline.org/article/S0161-4754(20)30194-9/fulltext>

https://www.sciencedirect.com/science/article/pii/S0161475420301949?via%3Dihub.

85. Chang SC, Hsu CH, Hsu CK, Yang SS, Chang SJ. The efficacy of acupuncture in managing patients with chronic prostatitis/chronic pelvic pain syndrome: A systemic review and meta-analysis.36(2):474-481. <https://onlinelibrary.wiley.com/doi/10.1002/nau.22958>.

86. Chao GQ, Zhang S. Effectiveness of acupuncture to treat irritable bowel syndrome: a meta-analysis.20(7):1871-1877. <https://www.ncbi.nlm.nih.gov/pmc/articles/PMC3930986/pdf/WJG-20-1871.pdf>.

87. Chau JPC, Lo SHS, Yu X, et al. Effects of Acupuncture on the Recovery Outcomes of Stroke Survivors with Shoulder Pain: A Systematic Review. 2018;9:30. <https://www.ncbi.nlm.nih.gov/pmc/articles/PMC5797784/pdf/fneur-09-00030.pdf>.

88. Chen C, Liu J, Sun MX, Liu WH, Han J, Wang HC. Acupuncture for type 2 diabetes mellitus: A systematic review and meta-analysis of randomized controlled trials.36:100-112. <https://www.sciencedirect.com/science/article/pii/S1744388119300052?via%3Dihub>.

89. Chen H, Liu Y, Wu J, Liang F, Liu Z. Acupuncture for postprostatectomy incontinence: a systematic review.

90. Chen H, Zhang W, Sun Y, Jiao R, Liu Z. The Role of Acupuncture in Relieving Post-Hemorrhoidectomy Pain: A Systematic Review of Randomized Controlled Trials. 2022;9:815618. <https://www.ncbi.nlm.nih.gov/pmc/articles/PMC8995644/pdf/fsurg-09-815618.pdf>.

91. Chen HY, Shi Y, Ng CS, Chan SM, Yung KKL, Zhang QL. Auricular acupuncture treatment for insomnia: A systematic review.13(6):669-676. <https://www.liebertpub.com/doi/10.1089/acm.2006.6400?url_ver=Z39.88-2003&rfr_id=ori:rid:crossref.org&rfr_dat=cr_pub%3dpubmed>

https://www.liebertpub.com/doi/pdf/10.1089/acm.2006.6400?download=true.

92. Chen J, Chen D, Ren Q, et al. Acupuncture and related techniques for obesity and cardiovascular risk factors: a systematic review and meta-regression analysis.38(4):227-234.

93. Chen J, Lin Z, Ding J. Zusanli (ST36) Acupoint Injection With Dexamethasone for Chemotherapy-Induced Myelosuppression: A Systematic Review and Meta-Analysis. 2021;11:684129. <https://www.ncbi.nlm.nih.gov/pmc/articles/PMC8291031/pdf/fonc-11-684129.pdf>.

94. Chen J, Ren Y, Tang Y, Li Z, Liang F. Acupuncture therapy for angina pectoris: a systematic review.32(4):494-501.

95. Chen L, Lin CC, Huang TW, et al. Effect of acupuncture on arornatase inhibitor-induced arthralgia in patients with breast cancer: A meta-analysis of randomized controlled trials.33:132-138. <https://www.thebreastonline.com/article/S0960-9776(17)30422-8/fulltext>

https://www.sciencedirect.com/science/article/pii/S0960977617304228?via%3Dihub.

96. Chen N, Wang J, Mucelli A, Zhang X, Wang C. Electro-Acupuncture is Beneficial for Knee Osteoarthritis: The Evidence from Meta-Analysis of Randomized Controlled Trials. 2017;45(5):965-985. <https://www.worldscientific.com/doi/10.1142/S0192415X17500513?url_ver=Z39.88-2003&rfr_id=ori:rid:crossref.org&rfr_dat=cr_pub%3dpubmed>.

97. Chen N, Zhou M, He L, Zhou D, Li N. Acupuncture for Bell's palsy.2010(8):Cd002914.

98. Chen PW, Zhong X, Dai YK, et al. The efficacy and safety of acupuncture in nonalcoholic fatty liver disease A systematic review and meta-analysis of randomized controlled trials.100(38).

99. Chen SW, Zhao M, Qiu JX. Acupuncture for the treatment of sudden sensorineural hearing loss: A systematic review and meta-analysis Acupuncture for SSNHL.42:381-388. <https://www.sciencedirect.com/science/article/pii/S0965229918304217?via%3Dihub>.

100. Chen W, Chen Z, Li J, et al. Electroacupuncture as an Adjuvant Approach to Rehabilitation during Postacute Phase after Total Knee Arthroplasty: A Systematic Review and Meta-Analysis of Randomized Controlled Trials. 2021;2021:9927699. <https://www.ncbi.nlm.nih.gov/pmc/articles/PMC8355970/pdf/ECAM2021-9927699.pdf>.

101. Chen W, Yang GY, Liu B, Manheimer E, Liu JP. Manual acupuncture for treatment of diabetic peripheral neuropathy: a systematic review of randomized controlled trials. 2013;8(9):e73764. <https://www.ncbi.nlm.nih.gov/pmc/articles/PMC3771980/pdf/pone.0073764.pdf>.

102. Chen X, Liu F, Lin N, et al. Warming needle moxibustion for Alzheimer's disease: A systematic review of randomized controlled trials.43:219-226. <https://www.sciencedirect.com/science/article/pii/S0197457221003815?via%3Dihub>.

103. Chen XT, Yang Y, Qian YM, et al. Acupuncture for acute gastrointestinal injury: A systematic review and meta-analysis 针灸治疗急性胃肠损伤的系统评价和Meta 分析. 2022. <https://www.embase.com/search/results?subaction=viewrecord&id=L2017625651&from=export> <http://dx.doi.org/10.1016/j.wjam.2022.03.008>.

104. Chen YC, Wu LK, Lee MS, Kung YL. The Efficacy of Acupuncture Treatment for Attention Deficit Hyperactivity Disorder: A Systematic Review and Meta-Analysis. 2021;28(4):357-367.

105. Chen YP, Liu T, Peng YY, et al. Acupuncture for hot flashes in women with breast cancer: A systematic review.12(2):535-542. <https://www.cancerjournal.net/article.asp?issn=0973-1482;year=2016;volume=12;issue=2;spage=535;epage=542;aulast=Chen>.

106. Chen YP, Peng MD, Li YQ. Acupuncture for the Adjunctive Therapy of Post-stoke Fatigue: A Systematic Review and Meta-analysis. 2021;47(1):115-128.

107. Chen ZH, Shen Z, Ye XL, et al. Acupuncture for Rehabilitation After Total Knee Arthroplasty: A Systematic Review and Meta-Analysis of Randomized Controlled Trials.7.

108. Chen ZH, Wang YT, Wang R, Xie J, Ren YL. Efficacy of Acupuncture for Treating Opioid Use Disorder in Adults: A Systematic Review and Meta-Analysis. 2018;2018.

109. Cheng K, Law A, Guo M, et al. Acupuncture for acute hordeolum. 2017(2). <http://dx.doi.org/10.1002/14651858.CD011075.pub2>.

110. Cheong KB, Zhang JP, Huang Y, Zhang ZJ. The effectiveness of acupuncture in prevention and treatment of postoperative nausea and vomiting--a systematic review and meta-analysis. 2013;8(12):e82474. <https://www.ncbi.nlm.nih.gov/pmc/articles/PMC3862842/pdf/pone.0082474.pdf>.

111. Cheuk DKL, Wong V. Acupuncture for epilepsy. 2014(5). <http://dx.doi.org/10.1002/14651858.CD005062.pub4>.

112. Cheuk DKL, Wong V, Chen WX. Acupuncture for autism spectrum disorders (ASD). 2011(9). <http://dx.doi.org/10.1002/14651858.CD007849.pub2>.

113. Cheuk DKL, Yeung WF, Chung KF, Wong V. Acupuncture for insomnia. 2007(3).

114. Chien TJ, Hsu CH, Liu CY, Fang CJ. Effect of acupuncture on hot flush and menopause symptoms in breast cancer- A systematic review and meta-analysis. 2017;12(8):e0180918. <https://www.ncbi.nlm.nih.gov/pmc/articles/PMC5568723/pdf/pone.0180918.pdf>.

115. Chien TJ, Liu CY, Chang YF, Fang CJ, Hsu CH. Acupuncture for treating aromatase inhibitor-related arthralgia in breast cancer: a systematic review and meta-analysis.21(5):251-260. <https://www.ncbi.nlm.nih.gov/pmc/articles/PMC4432489/pdf/acm.2014.0083.pdf>.

116. Chien TJ, Liu CY, Fang CJ. The Effect of Acupuncture in Breast Cancer–Related Lymphoedema (BCRL): A Systematic Review and Meta-Analysis. 2019;18. <https://www.embase.com/search/results?subaction=viewrecord&id=L628845389&from=export> <http://dx.doi.org/10.1177/1534735419866910>.

117. Chien TJ, Liu CY, Fang CJ, Kuo CY. The maintenance effect of acupuncture on breast cancer-related menopause symptoms: a systematic review.23(2):130-139. <https://www.tandfonline.com/doi/full/10.1080/13697137.2019.1664460>.

118. Chiu HY, Hsieh YJ, Tsai PS. Acupuncture to Reduce Sleep Disturbances in Perimenopausal and Postmenopausal Women: A Systematic Review and Meta-analysis.127(3):507-515.

119. Chiu HY, Hsieh YJ, Tsai PS. Systematic review and meta-analysis of acupuncture to reduce cancer-related pain.26(2).

120. Chiu HY, Pan CH, Shyu YK, Han BC, Tsai PS. Effects of acupuncture on menopause-related symptoms and quality of life in women in natural menopause: a meta-analysis of randomized controlled trials.22(2):234-244.

121. Cho KH, Kim TH, Jung WS, et al. Pharmacoacupuncture for Idiopathic Parkinson's Disease: A Systematic Review of Randomized Controlled Trials. 2018;2018:3671542. <https://www.ncbi.nlm.nih.gov/pmc/articles/PMC6036809/pdf/ECAM2018-3671542.pdf>.

122. Cho SH, Hwang EW. Acupuncture for primary dysmenorrhoea: a systematic review.117(5):509-521. <https://obgyn.onlinelibrary.wiley.com/doi/10.1111/j.1471-0528.2010.02489.x>.

123. Cho SH, Kim J. Efficacy of acupuncture in management of premenstrual syndrome: a systematic review.18(2):104-111. <https://www.sciencedirect.com/science/article/pii/S096522990900082X?via%3Dihub>.

124. Cho SH, Lee H, Ernst E. Acupuncture for pain relief in labour: a systematic review and meta-analysis.117(8):907-920. <https://obgyn.onlinelibrary.wiley.com/doi/10.1111/j.1471-0528.2010.02570.x>.

125. Cho SH, Lee JS, Thabane L, Lee J. Acupuncture for obesity: a systematic review and meta-analysis.33(2):183-196. <https://www.nature.com/articles/ijo2008269>.

126. Cho SH, Whang WW. Acupuncture for vasomotor menopausal symptoms: a systematic review.16(5):1065-1073.

127. Cho SH, Whang WW. Acupuncture for alcohol dependence: a systematic review.33(8):1305-1313. <https://onlinelibrary.wiley.com/doi/10.1111/j.1530-0277.2009.00959.x>.

128. Cho WC, Li C, Chen HY. Clinical efficacy of acupoint embedment in weight control: A systematic review and meta-analysis.97(36):e12267. <https://www.ncbi.nlm.nih.gov/pmc/articles/PMC6133431/pdf/medi-97-e12267.pdf>.

129. Cho YH, Kim CK, Heo KH, et al. Acupuncture for acute postoperative pain after back surgery: a systematic review and meta-analysis of randomized controlled trials.15(3):279-291. <https://www.ncbi.nlm.nih.gov/pmc/articles/PMC4409074/pdf/papr0015-0279.pdf>.

130. Choi GH, Wiel, LS, et al. Acupuncture and related interventions for the treatment of symptoms associated with carpal tunnel syndrome.12(12):Cd011215.

131. Choi TY, Kim JI, Lim HJ, Lee MS. Acupuncture for Managing Cancer-Related Insomnia: A Systematic Review of Randomized Clinical Trials.16(2):135-146. <https://www.ncbi.nlm.nih.gov/pmc/articles/PMC5739128/pdf/10.1177_1534735416664172.pdf>.

132. Choi TY, Lee MS, Ernst E. Acupuncture for cancer patients suffering from hiccups: a systematic review and meta-analysis.20(6):447-455. <https://www.sciencedirect.com/science/article/pii/S0965229912001112?via%3Dihub>.

133. Choi TY, Lee MS, Kim TH, Zaslawski C, Ernst E. Acupuncture for the treatment of cancer pain: a systematic review of randomised clinical trials.20(6):1147-1158. <https://link.springer.com/content/pdf/10.1007/s00520-012-1432-9.pdf>.

134. Chung SY, Noh BJ, Lee CW, et al. Acupuncture for Tourette syndrome: A systematic review and meta-analysis.8(5):809-816.

135. Clark RJ, Tighe M. The effectiveness of acupuncture for plantar heel pain: a systematic review.30(4):298-306.

136. Cooper K, Martyn-St James M, Kaltenthaler E, et al. Complementary and Alternative Medicine for Management of Premature Ejaculation: A Systematic Review.5(1):e1-e18. <https://www.ncbi.nlm.nih.gov/pmc/articles/PMC5302385/pdf/main.pdf>.

137. Cox J, Varatharajan S, Côté P, Optima C. Effectiveness of Acupuncture Therapies to Manage Musculoskeletal Disorders of the Extremities: A Systematic Review.46(6):409-429.

138. Coyle M, Deng J, Zhang AL, et al. Acupuncture therapies for psoriasis vulgaris: a systematic review of randomized controlled trials. 2015;22(2):102-109.

139. Coyle ME, Stupans I, Abdel-Nour K, et al. Acupuncture versus placebo acupuncture for in vitro fertilisation: a systematic review and meta-analysis.39(1):20-29.

140. Cui W, Sun W, Mao-Ying QL, Mi W, Chu Y, Wang Y. Evaluation of catgut implantation at acupoints for asthma: A systematic review and meta-analysis. 2018;1(2):123-132. <https://www.embase.com/search/results?subaction=viewrecord&id=L2015915457&from=export> <http://dx.doi.org/10.1142/S2575900018400025>.

141. Cui XM, Zhou J, Qin ZS, Liu ZS. Acupuncture for Erectile Dysfunction: A Systematic Review. 2016;2016.

142. Cui Y, Li Q, Wang DL, et al. Does electroacupuncture benefit mixed urinary incontinence? A systematic review and meta-analysis with trial sequential analysis %J INTERNATIONAL UROGYNECOLOGY JOURNAL.

143. Dai L, Liu Y, Ji G, Xu Y. Acupuncture and Derived Therapies for Pain in Palliative Cancer Management: Systematic Review and Meta-Analysis Based on Single-Arm and Controlled Trials.24(7):1078-1099. <https://www.liebertpub.com/doi/10.1089/jpm.2020.0405?url_ver=Z39.88-2003&rfr_id=ori:rid:crossref.org&rfr_dat=cr_pub%3dpubmed>

https://www.liebertpub.com/doi/pdf/10.1089/jpm.2020.0405?download=true.

144. Dai L, Ooi VV, Zhou WJ, Ji G. Acupoint embedding therapy improves nonalcoholic fatty liver disease with abnormal transaminase A PRISMA-compliant systematic review and meta-analysis.99(3).

145. Davis MA, Kononowech RW, Rolin SA, Spierings EL. Acupuncture for tension-type headache: a meta-analysis of randomized, controlled trials.9(8):667-677. <https://www.jpain.org/article/S1526-5900(08)00503-8/fulltext>.

146. Deare JC, Zheng Z, Xue CCL, et al. Acupuncture for treating fibromyalgia. 2013(5). <http://dx.doi.org/10.1002/14651858.CD007070.pub2>.

147. Deng M, Wang XF. Acupuncture for amnestic mild cognitive impairment: a meta-analysis of randomised controlled trials.34(5):342-348.

148. Dimitrova A, Murchison C, Oken B. Acupuncture for the Treatment of Peripheral Neuropathy: A Systematic Review and Meta-Analysis.23(3):164-179. <https://www.ncbi.nlm.nih.gov/pmc/articles/PMC5359694/pdf/acm.2016.0155.pdf>.

149. Dodin S, Blanchet C, Marc I, et al. Acupuncture for menopausal hot flushes. 2013;2013(7). <https://www.embase.com/search/results?subaction=viewrecord&id=L620561305&from=export> <http://dx.doi.org/10.1002/14651858.CD007410.pub2>.

150. Dong B, Chen Z, Yin X, et al. The Efficacy of Acupuncture for Treating Depression-Related Insomnia Compared with a Control Group: A Systematic Review and Meta-Analysis. 2017;2017:9614810. <https://www.ncbi.nlm.nih.gov/pmc/articles/PMC5329663/pdf/BMRI2017-9614810.pdf>.

151. Dong B, Lin L, Chen QY, et al. Wrist-ankle acupuncture has a positive effect on cancer pain: a meta-analysis.21(1).

152. Ee CC, Manheimer E, Pirotta MV, White AR. Acupuncture for pelvic and back pain in pregnancy: a systematic review.198(3):254-259. <https://www.ajog.org/article/S0002-9378(07)02115-1/fulltext>

<https://www.sciencedirect.com/science/article/pii/S0002937807021151?via%3Dihub>.

153. Ernst E, White AR. Acupuncture as a treatment for temporomandibular joint dysfunction: a systematic review of randomized trials.125(3):269-272. <https://jamanetwork.com/journals/jamaotolaryngology/articlepdf/509211/ooa8135.pdf>.

154. Ezzo J, Hadhazy V, Birch S, et al. Acupuncture for osteoarthritis of the knee - A systematic review.44(4):819-825. <https://onlinelibrary.wiley.com/doi/pdfdirect/10.1002/1529-0131%28200104%2944%3A4%3C819%3A%3AAID-ANR138%3E3.0.CO%3B2-P?download=true>.

155. Ezzo JM, Richardson MA, Vickers A, et al. Acupuncture-point stimulation for chemotherapy-induced nausea or vomiting. 2006(2).

156. Fan W, Kuang X, Hu J, et al. Acupuncture therapy for poststroke spastic hemiplegia: A systematic review and meta-analysis of randomized controlled trials.40:101176. <https://www.sciencedirect.com/science/article/pii/S1744388120303388?via%3Dihub>.

157. Fang S, Wang M, Zheng Y, Zhou S, Ji G. Acupuncture and Lifestyle Modification Treatment for Obesity: A Meta-Analysis. 2017;45(2):239-254. <https://www.worldscientific.com/doi/10.1142/S0192415X1750015X?url_ver=Z39.88-2003&rfr_id=ori:rid:crossref.org&rfr_dat=cr_pub%3dpubmed>.

158. Farag AM, Malacarne A, Pagni SE, Maloney GE. The effectiveness of acupuncture in the management of persistent regional myofascial head and neck pain: A systematic review and meta-analysis.49:102297. <https://www.sciencedirect.com/science/article/pii/S096522991930202X?via%3Dihub>.

159. Feng SY, Han MM, Fan YP, et al. Acupuncture for the treatment of allergic rhinitis: A systematic review and meta-analysis.29(1):57-62.

160. Fernández-de-Las-Peñas C, Pérez-Bellmunt A, Llurda-Almuzara L, Plaza-Manzano G, De-la-Llave-Rincón AI, Navarro-Santana MJ. Is Dry Needling Effective for the Management of Spasticity, Pain, and Motor Function in Post-Stroke Patients? A Systematic Review and Meta-Analysis.22(1):131-141. <https://watermark.silverchair.com/pnaa392.pdf?token=AQECAHi208BE49Ooan9kkhW_Ercy7Dm3ZL_9Cf3qfKAc485ysgAAAs8wggLLBgkqhkiG9w0BBwagggK8MIICuAIBADCCArEGCSqGSIb3DQEHATAeBglghkgBZQMEAS4wEQQM22U2UuRHCY-kONIZAgEQgIICgn7RPIhXavFJifjbaLqFc9ebPhLqiU8Xwm_b-O9z8C9IVaaClv_KsTmYLZ92qunMSZrgdXq1nEyiZ5HYWk5hvu1WAPETEHG82UJ1I6XeNzS0VJByucrCPC7Pak7anBxytO9XYENQmPWZshFsVOVa_Se5qXPruf5-RJMK37AZmFkqnrrMqUjAbfBkMQ4iYoFlxj3lBhgIFXK3FkR3AGY5LP2Mjj9UFOBCG9nb9A-pI6hsr7_toVhFN_3IIrmRJmxN9MHuzQWwtvVlzPo1wgeG9Ri30AVnMnXL_417-1CbMcLOSVv49t9aXm2_vTo9lfOkW6pIPFApHW8I2n3g3haLdS9RFIE-LC9qTW8m75X5bN_Yaha6k24Qbe9QBmZkRJ8op_ayPIdsch0aD2CUxoItUVEtZIYmP27QLVxJax81-A5E-lp2XCG_YgIxDOQDWSDkEGnEKqDhtHfQqagZG8ysduQPKPOxQTRYL4tzmhMVlu_ZsA3NTX02yNuSjkpNVMF-jo0x5nTZ0_XfCMlXcahsciK1Fd2zNCBEEjW5GTDZa1fW5oaYpvBOF4rkdx6N7VsEBiToJoC4cQEO5UTj8yf4iQRS0-Ohrkc39e8dZVM4DjRg6du8Jfcz2Po9AxFl1yUGVlZ3ya97E-zycABAGi-STuwr320kRO2bo3JJVTaIrxCrXZtUVi_-3CiZ1HZjCn2Cg7rVNexjqleOxqEOxAqr03BYnFjPKzZ_osHLqo17QAX1TA-syY3LSDvnLDZzkG--RpJAmOk8VAKfFD2PqbZ7-ZgbbtUHsZrz3HpOZNXAziEEW-QYmddnr-XVK57Hwfe52Vgw_wsfAb60UVU2h3PBtb9LVw>.

161. Fernández-Jané C, Vilaró J, Fei Y, et al. Acupuncture techniques for COPD: a systematic review.20(1):138. <https://www.ncbi.nlm.nih.gov/pmc/articles/PMC7323612/pdf/12906_2020_Article_2899.pdf>.

162. Fern, ez-Jane C, Vilaro J, et al. Filiform needle acupuncture for copd: A systematic review and meta-analysis.47.

163. France S, Bown J, Nowosilskyj M, Mott M, S, Walters J. Evidence for the use of dry needling and physiotherapy in the management of cervicogenic or tension-type headache: a systematic review.34(12):994-1003.

164. Fu LM, Li JT, Wu WS. Randomized controlled trials of acupuncture for neck pain: systematic review and meta-analysis.15(2):133-145. <https://www.liebertpub.com/doi/10.1089/acm.2008.0135?url_ver=Z39.88-2003&rfr_id=ori:rid:crossref.org&rfr_dat=cr_pub%3dpubmed>

https://www.liebertpub.com/doi/pdf/10.1089/acm.2008.0135?download=true.

165. Furlan AD, van Tulder MW, Cherkin D, et al. Acupuncture and dry‐needling for low back pain. 2005(1). <http://dx.doi.org/10.1002/14651858.CD001351.pub2>.

166. Gao X, Zhang Y, Zhang Y, Ku Y, Guo Y. Electroacupuncture for Gastrointestinal Function Recovery after Gynecological Surgery: A Systematic Review and Meta-Analysis. 2021;2021:8329366. <https://www.ncbi.nlm.nih.gov/pmc/articles/PMC8714373/pdf/ECAM2021-8329366.pdf>.

167. Garcia MK, Graham-Getty L, Haddad R, et al. Systematic review of acupuncture to control hot flashes in cancer patients.121(22):3948-3958. <https://www.ncbi.nlm.nih.gov/pmc/articles/PMC4635055/pdf/nihms717276.pdf>.

168. Gates S, Smith LA, Foxcroft DR. Auricular acupuncture for cocaine dependence. (1):Cd005192.

169. Ge S, Lan J, Yi Q, Wen H, Lu L, Tang C. Acupuncture for illicit drug withdrawal syndrome: A systematic review and meta-analysis. 2020;35. <https://www.embase.com/search/results?subaction=viewrecord&id=L2005191425&from=export> <http://dx.doi.org/10.1016/j.eujim.2020.101096>.

170. Giovanardi CM, Cinquini M, Aguggia M, et al. Acupuncture vs. Pharmacological Prophylaxis of Migraine: A Systematic Review of Randomized Controlled Trials. 2020;11:576272. <https://www.ncbi.nlm.nih.gov/pmc/articles/PMC7773012/pdf/fneur-11-576272.pdf>.

171. Grant S, Colaiaco B, Motala A, Shanman R, Sorbero M, Hempel S. Acupuncture for the Treatment of Adults with Posttraumatic Stress Disorder: A Systematic Review and Meta-Analysis.19(1):39-58. <https://www.tandfonline.com/doi/full/10.1080/15299732.2017.1289493>.

172. Grant S, rack R, Motala A, et al. Acupuncture for substance use disorders: A systematic review and meta-analysis.163:1-15. <https://www.sciencedirect.com/science/article/pii/S0376871616001095?via%3Dihub>.

173. Green S, Buchbinder R, Barnsley L, et al. Acupuncture for lateral elbow pain. 2002(1). <http://dx.doi.org/10.1002/14651858.CD003527>.

174. Green S, Buchbinder R, Hetrick S. Acupuncture for shoulder pain. (2):Cd005319.

175. Guo TP, Ren YL, Kou J, Shi J, Sun TX, Liang FR. Acupoint Catgut Embedding for Obesity: Systematic Review and Meta-Analysis. 2015;2015.

176. Hai YC, Shi Y, Chi SN, Sai MC, Yung KKL, Qing LZ. Auricular acupuncture treatment for insomnia: A systematic review. 2007;13(6):669-676. <https://www.embase.com/search/results?subaction=viewrecord&id=L47357089&from=export> <http://dx.doi.org/10.1089/acm.2006.6400>.

177. Hall ML, Mackie AC, Ribeiro DC. Effects of dry needling trigger point therapy in the shoulder region on patients with upper extremity pain and dysfunction: a systematic review with meta-analysis.104(2):167-177. <https://www.physiotherapyjournal.com/article/S0031-9406(17)30079-2/fulltext>

https://www.sciencedirect.com/science/article/pii/S0031940617300792?via%3Dihub.

178. Han J, Wang HC, Rong PJ, et al. Systematic review and meta-analysis of the therapeutic effect on functional dyspepsia treated with acupuncture and electroacupuncture.31(1):44-51.

179. Han X, Shen HY, Chen JM, Wu Y. Efficacy and safety of electrical stimulation for stress urinary incontinence in women: a systematic review and meta-analysis %J INTERNATIONAL UROGYNECOLOGY JOURNAL.

180. Hao CZ, Wu F, Guo Y, et al. Acupuncture for neurogenic bladder after spinal cord injury: A systematic review and meta-analysis.5(2):100-108.

181. He L, Zhou D, Wu B, Li N, Zhou MK. Acupuncture for Bell's palsy. 2004(1):Cd002914.

182. He L, Zhou MK, Zhou D, et al. Acupuncture for Bell's palsy. 2007(4). <https://www.embase.com/search/results?subaction=viewrecord&id=L351805132&from=export> <http://dx.doi.org/10.1002/14651858.CD002914.pub3>.

183. He M, Li X, Liu Y, et al. Electroacupuncture for Tinnitus: A Systematic Review. 2016;11(3):e0150600. <https://www.ncbi.nlm.nih.gov/pmc/articles/PMC4777560/pdf/pone.0150600.pdf>.

184. Heo I, Shin BC, Kim YD, Hwang EH, Han CW, Heo KH. Acupuncture for spinal cord injury and its complications: a systematic review and meta-analysis of randomized controlled trials. 2013;2013:364216. <https://www.ncbi.nlm.nih.gov/pmc/articles/PMC3586459/pdf/ECAM2013-364216.pdf>.

185. Hong QX, Hong M, Ying L, Cai W, Hua CX. Acupuncture compares with Western medicine for climacteric depression. 2016;11:S144-S153.

186. Hong S, Ahn L, Kwon J, Choi DJ. Acupuncture for Regulating Blood Pressure of Stroke Patients: A Systematic Review and Meta-Analysis.26(12):1105-1116. <https://www.liebertpub.com/doi/10.1089/acm.2019.0454?url_ver=Z39.88-2003&rfr_id=ori:rid:crossref.org&rfr_dat=cr_pub%3dpubmed>

<https://www.liebertpub.com/doi/pdf/10.1089/acm.2019.0454?download=true>.

187. Hou WZ, Pei LX, Song YF, et al. Acupuncture therapy for breast cancer-related lymphedema: A systematic review and meta-analysis.45(12):2307-2317. <https://obgyn.onlinelibrary.wiley.com/doi/10.1111/jog.14122>.

188. Hou Z, Xu S, Li Q, et al. The Efficacy of Acupuncture for the Treatment of Cervical Vertigo: A Systematic Review and Meta-Analysis. 2017;2017:7597363. <https://www.ncbi.nlm.nih.gov/pmc/articles/PMC5474245/pdf/ECAM2017-7597363.pdf>.

189. Hsieh PC, Yang MC, Wu YK, et al. Acupuncture therapy improves health-related quality of life in patients with chronic obstructive pulmonary disease: A systematic review and meta-analysis.35:208-218. <https://www.sciencedirect.com/science/article/pii/S1744388119300568?via%3Dihub>.

190. Hu CQ, Zhang HB, Wu WY, et al. Acupuncture for Pain Management in Cancer: A Systematic Review and Meta-Analysis. 2016;2016.

191. Hu H, Chen L, Ma R, Gao H, Fang J. Acupuncture for primary trigeminal neuralgia: A systematic review and PRISMA-compliant meta-analysis.34:254-267. <https://www.sciencedirect.com/science/article/pii/S1744388118305498?via%3Dihub>.

192. Hu HT, Gao H, Ma RJ, Zhao XF, Tian HF, Li L. Is dry needling effective for low back pain?: A systematic review and PRISMA-compliant meta-analysis.97(26):e11225. <https://www.ncbi.nlm.nih.gov/pmc/articles/PMC6242300/pdf/medi-97-e11225.pdf>.

193. Hu XY, Trevelyan E, Yang GY, et al. The effectiveness of acupuncture/TENS for phantom limb syndrome. I: A systematic review of controlled clinical trials.6(3):355-364.

194. Huang CW, Wang ZP, Xu XL, Hu SS, Zhu R, Chen X. Does Acupuncture Benefit Delayed-Onset Muscle Soreness After Strenuous Exercise? A Systematic Review and Meta-Analysis.11.

195. Huang F, Xie Y, Zhao S, Feng Z, Chen G, Xu Y. The Effectiveness and Safety of Acupoint Catgut Embedding for the Treatment of Postmenopausal Osteoporosis: A Systematic Review and Meta-Analysis. 2019;2019:2673763. <https://www.ncbi.nlm.nih.gov/pmc/articles/PMC6710781/pdf/ECAM2019-2673763.pdf>.

196. Huang J, Shi Y, Qin X, Shen M, Wu M, Huang Y. Clinical Effects and Safety of Electroacupuncture for the Treatment of Poststroke Dysphagia: A Comprehensive Systematic Review and Meta-Analysis. 2020;2020:1560978. <https://www.ncbi.nlm.nih.gov/pmc/articles/PMC7533748/pdf/ECAM2020-1560978.pdf>.

197. Huang JJ, Liang JQ, Xu XK, Xu YX, Chen GZ. Safety of Thread Embedding Acupuncture Therapy: A Systematic Review.27(12):947-955. <https://link.springer.com/content/pdf/10.1007/s11655-021-3443-1.pdf>.

198. Huang KY, Liang S, Chen L, Grellet A. Acupuncture for tinnitus: a systematic review and meta-analysis of randomized controlled trials.39(4):264-271.

199. Huang Q, Luo D, Chen L, Liang FX, Chen R. Effectiveness of Acupuncture for Alzheimer's Disease: An Updated Systematic Review and Meta-analysis.39(3):500-511. <https://link.springer.com/content/pdf/10.1007/s11596-019-2065-8.pdf>.

200. Huang W, Kutner N, Bliwise DL. A systematic review of the effects of acupuncture in treating insomnia.13(1):73-104. <https://www.sciencedirect.com/science/article/pii/S1087079208000270?via%3Dihub>.

201. Huang YJ, Huang CS, Leng KF, Sung JY, Cheng SW. Efficacy of Scalp Acupuncture in Patients With Post-stroke Hemiparesis: Meta-Analysis of Randomized Controlled Trials. 2021;12:746567. <https://www.ncbi.nlm.nih.gov/pmc/articles/PMC8695983/pdf/fneur-12-746567.pdf>.

202. Huh JH, Jeong HI, Kim KH. Effect of Manual Acupuncture for Mild-to-Moderate Carpal Tunnel Syndrome: A Systematic Review.24(4):153-164. <https://www.ncbi.nlm.nih.gov/pmc/articles/PMC8716703/pdf/jop-24-4-153.pdf>.

203. Hwang MS, Lee HY, Choi TY, et al. A systematic review and meta-analysis of the efficacy of acupuncture and electroacupuncture against chemotherapy-induced peripheral neuropathy.99(17):e19837. <https://www.ncbi.nlm.nih.gov/pmc/articles/PMC7220547/pdf/medi-99-e19837.pdf>.

204. Jan AL, Aldridge ES, Rogers IR, Visser EJ, Bulsara MK, Niemtzow RC. Does Ear Acupuncture Have a Role for Pain Relief in the Emergency Setting? A Systematic Review and Meta-Analysis.29(5):276-289. <https://www.ncbi.nlm.nih.gov/pmc/articles/PMC5653340/pdf/acu.2017.1237.pdf>.

205. Jang A, Brown C, Lamoury G, et al. The Effects of Acupuncture on Cancer-Related Fatigue: Updated Systematic Review and Meta-Analysis.19:1534735420949679. <https://www.ncbi.nlm.nih.gov/pmc/articles/PMC7533944/pdf/10.1177_1534735420949679.pdf>.

206. Jang S, Kim KH, You S. Acupuncture for in vitro fertilization in women with poor ovarian response: a systematic review. 2020;9. <https://www.embase.com/search/results?subaction=viewrecord&id=L2007697645&from=export> <http://dx.doi.org/10.1016/j.imr.2020.100562>.

207. Jang S, Ko Y, Sasaki Y, et al. Acupuncture as an adjuvant therapy for management of treatment-related symptoms in breast cancer patients: Systematic review and meta-analysis (PRISMA-compliant).99(50):e21820. <https://www.ncbi.nlm.nih.gov/pmc/articles/PMC7738093/pdf/medi-99-e21820.pdf>.

208. Jedel E. Acupuncture in xerostomia--a systematic review.32(6):392-396. <https://onlinelibrary.wiley.com/doi/10.1111/j.1365-2842.2005.01445.x>.

209. Jerng UM, Jo JY, Lee S, Lee JM, Kwon O. The effectiveness and safety of acupuncture for poor semen quality in infertile males: a systematic review and meta-analysis.16(6):884-891. <https://www.ncbi.nlm.nih.gov/pmc/articles/PMC4236334/pdf/AJA-16-884.pdf>.

210. Ji M, Wang XX, Chen MJ, Shen Y, Zhang X, Yang J. The Efficacy of Acupuncture for the Treatment of Sciatica: A Systematic Review and Meta-Analysis. 2015;2015.

211. Jia W, Wang CA, Yin Y. Acupuncture for oligospermia and asthenozoospermia A systematic review and meta-analysis.100(48).

212. Jiang YB, Shi X, Tang Y. Efficacy and safety of acupuncture therapy for nerve deafness: a meta-analysis of randomized controlled trials. 2015;8(2):2614-2620. <https://www.ncbi.nlm.nih.gov/pmc/articles/PMC4402856/pdf/ijcem0008-2614.pdf>.

213. Jin Y, Wang Y, Zhang J, Xiao X, Zhang Q. Efficacy and Safety of Acupuncture against Chemotherapy-Induced Peripheral Neuropathy: A Systematic Review and Meta-Analysis. 2020;2020:8875433. <https://www.ncbi.nlm.nih.gov/pmc/articles/PMC7669337/pdf/ECAM2020-8875433.pdf>.

214. Jo J, Lee YJ. Effectiveness of acupuncture in women with polycystic ovarian syndrome undergoing in vitro fertilisation or intracytoplasmic sperm injection: a systematic review and meta-analysis.35(3):162-170.

215. Jo J, Lee YJ, Lee H. Effectiveness of Acupuncture for Primary Ovarian Insufficiency: A Systematic Review and Meta-Analysis. 2015;2015:842180. <https://www.ncbi.nlm.nih.gov/pmc/articles/PMC4451156/pdf/ECAM2015-842180.pdf>.

216. Jo J, Lee YJ, Lee H. Acupuncture for polycystic ovarian syndrome: A systematic review and meta-analysis.96(23):e7066. <https://www.ncbi.nlm.nih.gov/pmc/articles/PMC5466220/pdf/medi-96-e7066.pdf>.

217. Ju ZY, Wang K, Cui HS, et al. Acupuncture for neuropathic pain in adults. 2017(12). <http://dx.doi.org/10.1002/14651858.CD012057.pub2>.

218. Jung A, Shin BC, Lee MS, Sim H, Ernst E. Acupuncture for treating temporomandibular joint disorders: a systematic review and meta-analysis of randomized, sham-controlled trials.39(5):341-350. <https://www.sciencedirect.com/science/article/pii/S0300571211000509?via%3Dihub>.

219. Kim H, Kim HK, Kim SY, Kim YI, Yoo HR, Jung IC. Cognitive improvement effects of electro-acupuncture for the treatment of MCI compared with Western medications: a systematic review and Meta-analysis.19(1):13. <https://www.ncbi.nlm.nih.gov/pmc/articles/PMC6325879/pdf/12906_2018_Article_2407.pdf>.

220. Kim JI, Choi JY, Lee DH, Choi TY, Lee MS, Ernst E. Acupuncture for the treatment of tinnitus: a systematic review of randomized clinical trials.12:97. <https://www.ncbi.nlm.nih.gov/pmc/articles/PMC3493359/pdf/1472-6882-12-97.pdf>.

221. Kim JI, Lee MS, Choi TY, Lee H, Kwon HJ. Acupuncture for Bell's palsy: a systematic review and meta-analysis.18(1):48-55. <https://link.springer.com/content/pdf/10.1007/s11655-011-0861-5.pdf>.

222. Kim KH, Kim TH, Lee BR, et al. Acupuncture for lumbar spinal stenosis: a systematic review and meta-analysis.21(5):535-556. <https://www.sciencedirect.com/science/article/pii/S0965229913001295?via%3Dihub>.

223. Kim KH, Lee BR, Ryu JH, Choi TY, Yang GY. The role of acupuncture in emergency department settings: a systematic review.21(1):65-72. <https://www.sciencedirect.com/science/article/pii/S0965229912001707?via%3Dihub>.

224. Kim KH, Lee MS, Choi SM, Ernst E. Acupuncture for Treating Uremic Pruritus in Patients with End-Stage Renal Disease: A Systematic Review.40(1):117-125. <https://www.jpsmjournal.com/article/S0885-3924(10)00276-9/pdf>.

225. Kim KH, Lee MS, Choi TY, Kim TH. Acupuncture for symptomatic gastroparesis.12(12):Cd009676.

226. Kim KH, Lee MS, Kim TH, Kang JW, Choi TY, Lee JD. Acupuncture and related interventions for symptoms of chronic kidney disease.2016(6):Cd009440.

227. Kim KN, Chung SY, Cho SH. Efficacy of acupuncture treatment for functional dyspepsia: A systematic review and meta-analysis.23(6):759-766. <https://www.sciencedirect.com/science/article/pii/S0965229915001132?via%3Dihub>.

228. Kim SH, Jeong JH, Lim JH, Kim BK. Acupuncture using pattern-identification for the treatment of insomnia disorder: a systematic review and meta-analysis of randomized controlled trials.8(3):216-226. <https://www.ncbi.nlm.nih.gov/pmc/articles/PMC6718809/pdf/main.pdf>.

229. Kim SY, Park HJ, Lee H, Lee H. Acupuncture for premenstrual syndrome: a systematic review and meta-analysis of randomised controlled trials.118(8):899-915. <https://obgyn.onlinelibrary.wiley.com/doi/10.1111/j.1471-0528.2011.02994.x>.

230. Kim SY, Shin IS, Park YJ. Effect of acupuncture and intervention types on weight loss: a systematic review and meta-analysis.19(11):1585-1596. <https://onlinelibrary.wiley.com/doi/pdfdirect/10.1111/obr.12747?download=true>.

231. Kim TH, Lee MS, Kim KH, Kang JW, Choi TY, Ernst E. Acupuncture for treating acute ankle sprains in adults. (6):Cd009065.

232. Kim YD, Heo I, Shin BC, Crawford C, Kang HW, Lim JH. Acupuncture for posttraumatic stress disorder: a systematic review of randomized controlled trials and prospective clinical trials. 2013;2013:615857. <https://www.ncbi.nlm.nih.gov/pmc/articles/PMC3580897/pdf/ECAM2013-615857.pdf>.

233. Ko GWY, Clarkson C. The effectiveness of acupuncture for pain reduction in delayed-onset muscle soreness: a systematic review.38(2):63-74.

234. Ko HF, Chen CH, Dong KR, Wu HC. Effects of Acupuncture on Postoperative Pain After Total Knee Replacement: Systematic Literature Review and Meta-Analysis.22(9):2117-2127. <https://watermark.silverchair.com/pnab201.pdf?token=AQECAHi208BE49Ooan9kkhW_Ercy7Dm3ZL_9Cf3qfKAc485ysgAAAtAwggLMBgkqhkiG9w0BBwagggK9MIICuQIBADCCArIGCSqGSIb3DQEHATAeBglghkgBZQMEAS4wEQQMMSpDGdvQrFf5_e59AgEQgIICg5JfQSV8AARF5wQsIifKDVkenBOAIbVi7URiSruRjMr-3CvfsDCmmIANdDWU82SS9lleEAhBzVlpotPGh3EiubFtk-9Q9TwDCVdPSblZcRhyrAQrHFFzxr8LJYmmvf0AucT6fzxpyNbC1N9dhLtPqaLRbRBZDSmw-HX8TvXon-H3-Ei3Pl7mWUz5qXi-j1UJWOT6-PyYVuxJYC5LF9zSo1pvNllt6oJbr1LxuKHrKXrSIIuYj-1p2iEh58QRwgXo4G9J6uHas3wrcfPMGiQCQfBKQY6gvV9-UPD_4Qx8zbCgSHjyClvW5mtk4ZyZYzfGnhgVFA_LgnxmDJklxRniDG8XPPesVlQuWJ0oz1fQGI2AUDHXDPJpHpnXCYsWn2EHr2EiHVhELbINVEiLaj2whwJyyYfRKSyhhbDzD44HeNgEgf9ba_3FWgIrgJsjRdFcacdvDIK7DkxkBLASn5b2NAOhxXxCPciGZj8LXJeRmOqbHw_EiRmewxwy3IMABFS-2dRe_onNtkl0BMdgScxRec70THvJ-FVptRtI4p5zrUqTEk5HjAs43ftfHURC7YbxzA8hf5gqLmnN369TQUIHCscnUMFZrrjHesOq3D-AKLvAgHarY3vJEU9q7dBVhTGX-uSRGfjlQI9USEI1UsC2hSsCLRJ5DjOdRRv3Zp7vCfj23ae5_7AYOVvERJipj9bxe5Ml4iSpiW7P_rTJTa8HJRUqaEAb6Ou901zfoULiEnPRnLuPHYc3D4rbr6PALodZIobk0mU1y-TW2Id_AfRU4ojFJ7ejZivyooWuihSN4nkUqEJrY-O_fIlrMxy-w1GiqF3E60MqgedyMG36EG7trtwUmF8>.

235. Koog YH, Lee JS, Wi H. Clinically meaningful nocebo effect occurs in acupuncture treatment: a systematic review.67(8):858-869. <https://www.jclinepi.com/article/S0895-4356(14)00089-4/fulltext>

<https://www.sciencedirect.com/science/article/pii/S0895435614000894?via%3Dihub>.

236. Kuang X, Fan WJ, Hu JW, et al. Acupuncture for post-stroke cognitive impairment: a systematic review and meta-analysis.39(6):577-588.

237. Kwon CY, Lee B, Ha DJ. Effectiveness and safety of acupuncture in treating sleep disturbance in dementia patients: A PRISMA-compliant systematic review and limitations of current evidence.100(32):e26871. <https://www.ncbi.nlm.nih.gov/pmc/articles/PMC8360405/pdf/medi-100-e26871.pdf>.

238. Kwon CY, Lee B, Kim SH, Tu WJ. Effectiveness and safety of ear acupuncture for trauma-related mental disorders after large-scale disasters: A PRISMA-compliant systematic review. 2020;99(8). <https://www.embase.com/search/results?subaction=viewrecord&id=L631011144&from=export> <http://dx.doi.org/10.1097/MD.0000000000019342>.

239. Kwon CY, Lee B, Suh HW, Chung SY, Kim JW. Efficacy and Safety of Auricular Acupuncture for Cognitive Impairment and Dementia: A Systematic Review. 2018;2018:3426078. <https://www.ncbi.nlm.nih.gov/pmc/articles/PMC6000857/pdf/ECAM2018-3426078.pdf>.

240. La Touche R, Goddard G, De-la-Hoz JL, et al. Acupuncture in the treatment of pain in temporomandibular disorders: a systematic review and meta-analysis of randomized controlled trials.26(6):541-550.

241. Lai XH, Zhang JP, Chen J, Lai CW, Huang CP. Is electroacupuncture safe and effective for treatment of stress urinary incontinence in women? A systematic review and meta-analysis.48(10).

242. Lam YC, Kum WF, Durairajan SS, et al. Efficacy and safety of acupuncture for idiopathic Parkinson's disease: a systematic review.14(6):663-671. <https://www.liebertpub.com/doi/10.1089/acm.2007.0011?url_ver=Z39.88-2003&rfr_id=ori:rid:crossref.org&rfr_dat=cr_pub%3dpubmed>

<https://www.liebertpub.com/doi/pdf/10.1089/acm.2007.0011?download=true>.

243. Lan J, Miao JF, Ge SQ, Chai TQ, Tang CZ, Lu LM. Acupuncture for cognitive impairment in vascular dementia, alzheimer's disease and mild cognitive impairment: A systematic review and meta-analysis. 2020;35. <https://www.embase.com/search/results?subaction=viewrecord&id=L2005097515&from=export> <http://dx.doi.org/10.1016/j.eujim.2020.101085>.

244. Lan L, Zeng F, Liu GJ, et al. Acupuncture for functional dyspepsia. (10):Cd008487.

245. Langhorst J, Klose P, Musial F, Irnich D, Häuser W. Efficacy of acupuncture in fibromyalgia syndrome--a systematic review with a meta-analysis of controlled clinical trials.49(4):778-788. <https://watermark.silverchair.com/kep439.pdf?token=AQECAHi208BE49Ooan9kkhW_Ercy7Dm3ZL_9Cf3qfKAc485ysgAAAtcwggLTBgkqhkiG9w0BBwagggLEMIICwAIBADCCArkGCSqGSIb3DQEHATAeBglghkgBZQMEAS4wEQQMLxtpAjAm_JfxBOohAgEQgIICiqUbglm-D4S5gyWyLI4eLWD68U8_P4vnT16vAYPfhhZyypntUdxAuWfOWY5R5nc2PJtbIleAUkcE-udPd8_tq6IAzx6O8Rb2qTznH6oyp7RJHQUdSnEDi1jwVGCGBMBciKWL7OU6hfzH_W84gdZZeRn1YeGVzvWw-Y8-ZXk3qy94zWgC_sgiHNDC8G1L6X4g7ylwhSAYtgpQXuLBOIhjTn1aocSoc2dWmkVBxTe0ABHA4mnQ_aKImXW8QfHzhG_pgfqqilau4rPbao93wr8AU6BYs_rGxU1XGZUjZ3VZNJwKWmOfIJCk7oUmCIO02tV6xWKQ_PpE0wTfjgTpkcQlfFS0TBmRfy74FutiBApEXcBs27lVi_PZ0rbCNewcPtfiSDZ2v20WC-zeFomUgzaXUTZ9JfJxUHQlDP5lsYDqguQ-46-VOg6cfhdbScB5epSQBPz-dFragZmqbQpRB-Kbov8vkwFMd4i2Us1JmMdwy0qllyewCkAVENTl_dy5KERk_6mUiPBluXd8TcCoKCwSlud9LDiVimkpzM7QDckrSadUQ5N2AklmIUFZPwZAkunPqx_bUQYbhsuY6A44Wr7oM5OYSBqZwCBTZmuXKyukbTa226J9y84nHgeMRS4rmXKG2y2U3jyQVIp3r0HSb-Su2664ZWRR2U6jjh8GelT9Aw6RVYJv5E2_2ePsNYE9xZdL2bV0-2mwLkVRMXaegp4IT3wDv8gezWtRztFBTL1Jqx5NjxcYbUeIWmDNMKWKJsIxwdBliHPIZoVcCDNH96QJEc48859LR-cnLcdyTDf7RUUQTrvQVJSI87d-DjHMAL9GAvitt5z-ZkMJSpzN1v5Xo9ZZyTZBPJfnRF7z>.

246. Lee A, Chan SKC, Fan LTY. Stimulation of the wrist acupuncture point PC6 for preventing postoperative nausea and vomiting. 2015(11).

247. Lee B, Lee J, Cheon JH, Sung HK, Cho SH, Chang GT. The Efficacy and Safety of Acupuncture for the Treatment of Children with Autism Spectrum Disorder: A Systematic Review and Meta-Analysis. 2018;2018:1057539. <https://www.ncbi.nlm.nih.gov/pmc/articles/PMC5820575/pdf/ECAM2018-1057539.pdf>.

248. Lee D, Lee H, Kim J, et al. Acupuncture for Infantile Colic: A Systematic Review of Randomised Controlled Trials. 2018;2018:7526234. <https://www.ncbi.nlm.nih.gov/pmc/articles/PMC6220386/pdf/ECAM2018-7526234.pdf>.

249. Lee H, Ernst E. Acupuncture for GI endoscopy: a systematic review.60(5):784-789. <https://www.giejournal.org/article/S0016-5107(04)02030-9/fulltext>

<https://www.sciencedirect.com/science/article/pii/S0016510704020309?via%3Dihub>.

250. Lee H, Ernst E. Acupuncture for labor pain management: A systematic review.191(5):1573-1579. <https://www.ajog.org/article/S0002-9378(04)00510-1/fulltext>

<https://www.sciencedirect.com/science/article/pii/S0002937804005101?via%3Dihub>.

251. Lee H, Kim SY, Park J, Kim YJ, Lee H, Park HJ. Acupuncture for lowering blood pressure: systematic review and meta-analysis.22(1):122-128. <https://watermark.silverchair.com/22_1_122.pdf?token=AQECAHi208BE49Ooan9kkhW_Ercy7Dm3ZL_9Cf3qfKAc485ysgAAAr8wggK7BgkqhkiG9w0BBwagggKsMIICqAIBADCCAqEGCSqGSIb3DQEHATAeBglghkgBZQMEAS4wEQQMTX9bQmbtqAIwxNTOAgEQgIICctvlNo9gqEG86p40pVtDsZQIKILhfNZLnh3oS57-XiCawr7HU5MUE3aW0fvCU3spwNUPEzUHpC806D17gX8GTjW48O5xv0ByEJ9tBSekUl9b2elf7Ncvy-4EXbmMwTSpVy0WuyxT4fRWe7vSpeY2EIdigxh76SxWUDEVAD5Mif4Y-VksHzwI4g5GeKrRwXaI-aspraAA2PVm-GwGNpx_WB9gJQVh55hnnAdpTLVQzEtmXImRHYY2DRi03COnFZQp6l6RHO5yiFrZgPPG7dqBw49P-nVov1CvSVTmsIADueQAwVytCGA0dhWh_UcTakPCEdoHfTFJ2mFS7WSMz6XzTgP7PU3CpwYibGkCkdEXOEXpIjQwTbdTnuIphijR6Bu6GF7lJ9LJNz8N_xqtPfsHR5xpDArSBViMEzw9_LyOM4rMZbdqAL_2P-v6yG0xpkpP11QuB-AyoX_sXVGxXQHJDpsxPUG-c4mQQEJkI8DIh20Ed8n_TtbbV1jbrOIpuRNwMUFAvtDUr7yYXb6p3EZ4plIzgEK19h8BUwuqW47ZNeB0-2hV3Wnlt0gZ0BWpQpMR8LD2j37XgHsrDpoAFce_M6Cyk3HFS1TcwHmjyDWDUqe1K6urHSpuuKHmrL6ELs_pfyxf4hsq9JWI0e77EwmxH5lJPXc0QYOROayKGsFpeEdDw1rjOCPnZCKkQEw1bPF_loccCK8-WgQrausF8gFf1lUhUndMrfwLq0GT2f6GenyRU46vJVB8avrR5fgvOMYie9XnCv90xZDstZRqlgJmniDBybjLSHi9Qp5XPnNMMb6YQUStHPvaXPK7uWYYn8A7XGM1>.

252. Lee H, Kim TH, Leem J. Acupuncture for heart failure: A systematic review of clinical studies.222:321-331. <https://www.internationaljournalofcardiology.com/article/S0167-5273(16)31589-3/fulltext>

<https://www.sciencedirect.com/science/article/pii/S0167527316315893?via%3Dihub>.

253. Lee H, Lee JH, Choi TY, Lee MS, Lee H, Shin BC. Acupuncture for acute low back pain: A systematic review. 2013;29(2):172-185. <https://www.embase.com/search/results?subaction=viewrecord&id=L368072771&from=export> <http://dx.doi.org/10.1097/AJP.0b013e31824909f9>.

254. Lee H, Schmidt K, Ernst E. Acupuncture for the relief of cancer-related pain--a systematic review.9(4):437-444. <https://onlinelibrary.wiley.com/doi/abs/10.1016/j.ejpain.2004.10.004>.

255. Lee HS, Park HL, Lee SJ, Shin BC, Choi JY, Lee MS. Scalp acupuncture for Parkinson's disease: a systematic review of randomized controlled trials.19(4):297-306. <https://link.springer.com/content/pdf/10.1007/s11655-013-1431-9.pdf>.

256. Lee JA, Son MJ, Choi J, Jun JH, Kim JI, Lee MS. Bee venom acupuncture for rheumatoid arthritis: a systematic review of randomised clinical trials.4(11):e006140. <https://www.ncbi.nlm.nih.gov/pmc/articles/PMC4225238/pdf/bmjopen-2014-006140.pdf>.

257. Lee JH, Jang E, Jung MH, Ha KT, Han C. Clinical effectiveness of acupuncture in the treatment of chemotherapy-induced leukopenia: A systematic review.8(5):802-808.

258. Lee JW, Lee JH, Kim SY. Use of acupuncture for the treatment of sportsrelated injuries in athletes: A systematic review of case reports. 2020;17(21):1-24. <https://www.embase.com/search/results?subaction=viewrecord&id=L2005411211&from=export> <http://dx.doi.org/10.3390/ijerph17218226>.

259. Lee MS, Choi TY, Shin BC, Ernst E. Acupuncture for children with autism spectrum disorders: a systematic review of randomized clinical trials.42(8):1671-1683. <https://link.springer.com/content/pdf/10.1007/s10803-011-1409-4.pdf>.

260. Lee MS, Kim KH, Choi SM, Ernst E. Acupuncture for treating hot flashes in breast cancer patients: a systematic review.115(3):497-503. <https://link.springer.com/content/pdf/10.1007/s10549-008-0230-z.pdf>.

261. Lee MS, Kim KH, Shin BC, Choi SM, Ernst E. Acupuncture for treating hot flushes in men with prostate cancer: a systematic review.17(7):763-770. <https://link.springer.com/content/pdf/10.1007/s00520-009-0589-3.pdf>.

262. Lee MS, Pittler MH, Shin BC, Kim JI, Ernst E. Acupuncture for allergic rhinitis: a systematic review.102(4):269-279; quiz 279-281, 307. <https://www.annallergy.org/article/S1081-1206(10)60330-4/fulltext>.

263. Lee MS, Pittler MH, Shin BC, Kong JC, Ernst E. Bee venom acupuncture for musculoskeletal pain: a review.9(4):289-297. <https://www.jpain.org/article/S1526-5900(07)01021-8/fulltext>.

264. Lee MS, Shin BC, Choi TY, Ernst E. Acupuncture for treating dry eye: a systematic review.89(2):101-106. <https://onlinelibrary.wiley.com/doi/pdfdirect/10.1111/j.1755-3768.2009.01855.x?download=true>.

265. Lee MS, Shin BC, Ernst E. Acupuncture for rheumatoid arthritis: a systematic review.47(12):1747-1753. <https://watermark.silverchair.com/ken330.pdf?token=AQECAHi208BE49Ooan9kkhW_Ercy7Dm3ZL_9Cf3qfKAc485ysgAAAtowggLWBgkqhkiG9w0BBwagggLHMIICwwIBADCCArwGCSqGSIb3DQEHATAeBglghkgBZQMEAS4wEQQMNUcAWV9m_cdos5PfAgEQgIICjSDlJZoc5GhkXAZ8SmZhus1gN_5Krt5H-02M2zqkWp4DBzxOyt55bIW_z9DVj8HdZ4CHHaj-seEMRgEuHL0ZZunxadQ-bt7HsjpbBYUG9wHu2FqJFJT1gN5_KqPQI5gG6bq38fX2izvfJXhWPtPEw-hs3ijlrfkebxO03kBZnpXBcxt1e9hf54UL-IJBSq4Pyr0-4OYKbAxwtFkZyl7afbmFFoJNyOWGmILxuD9d_C5Q6Xy4Q9sLlG3sq3IXKKdMOUOGl50SCV0jslIFblUHzMgyVALoMMEXnPiVVQfTV_SEGLrx-95NjWGNB6m8ctzfjGfwABctwan7mcg-_9sXs7t6Nk_RMyBSnlSLZBt0n5a5SlBnHnR7T6l7aYIX0lbm8vjpRL6Nqeh6tbjsZ6KLAdMWPeMhktwzT8WhxzT4fh9OG81-xVQCfqh7F6yGx5espZxSApim_hpgaaxFElGjV-DaJXzmPBFzt9cFBeXVRxSvz3_KWawYU3G6qMTWyq0OJwdvo0HTS2Qsu0ry-75mLjnpVZE8bq08JdJ8uS0nKAaUjsIvzIICUQgzUQmvu931J3xm4aJQfuFLPw7cUzsT5ahvvgOM6SzShbTS0c98SOLFb9l53QbRcWaAyeO_kZbPZYynQQ4yBdZHl4g4HmMi3M_dMVwCiFT_aXiYja68LT06nXfiggANVxgP1V5FSVAMpJYtIrVPgnLDYKyPpqSmVDxQbnWwMdnE_FNovwQIh_tC00u8h45UbmRGRfuujaMn5L7KgxpX0E6O6_ppyaRgG85jxM-lnS0fcD46HnKNTn5tABywnfcgE7EkYxiDdBYXO5zbnDHjvsbgLSrVz7dGKp2ySBIhC85pG5mMEKb5>.

266. Lee MS, Shin BC, Ernst E. Acupuncture for treating erectile dysfunction: a systematic review.104(3):366-370. <https://bjui-journals.onlinelibrary.wiley.com/doi/10.1111/j.1464-410X.2009.08422.x>.

267. Lee MS, Shin BC, Ernst E. Acupuncture for treating menopausal hot flushes: a systematic review.12(1):16-25. <https://www.tandfonline.com/doi/full/10.1080/13697130802566980>.

268. Lee MS, Shin BC, Kong JC, Ernst E. Effectiveness of acupuncture for Parkinson disease: A systematic review.23(11):1505-1515. <https://movementdisorders.onlinelibrary.wiley.com/doi/10.1002/mds.21993>.

269. Lee MS, Shin BC, Ronan P, Ernst E. Acupuncture for schizophrenia: a systematic review and meta-analysis.63(11):1622-1633. <https://onlinelibrary.wiley.com/doi/pdfdirect/10.1111/j.1742-1241.2009.02167.x?download=true>.

270. Lee MS, Shin BC, Suen LK, Park TY, Ernst E. Auricular acupuncture for insomnia: a systematic review.62(11):1744-1752. <https://onlinelibrary.wiley.com/doi/10.1111/j.1742-1241.2008.01876.x>.

271. Lee SH, Lim SM. Acupuncture for insomnia after stroke: a systematic review and meta-analysis.16:228. <https://www.ncbi.nlm.nih.gov/pmc/articles/PMC4950252/pdf/12906_2016_Article_1220.pdf>.

272. Lee SH, Lim SM. Acupuncture for Poststroke Shoulder Pain: A Systematic Review and Meta-Analysis. 2016;2016:3549878. <https://www.ncbi.nlm.nih.gov/pmc/articles/PMC4983325/pdf/ECAM2016-3549878.pdf>.

273. Lee SJ, Shin BC, Lee MS, Han CH, Kim JI. Scalp acupuncture for stroke recovery: A systematic review and meta-analysis of randomized controlled trials.5(2):87-99.

274. Lew J, Kim J, Nair P. Comparison of dry needling and trigger point manual therapy in patients with neck and upper back myofascial pain syndrome: a systematic review and meta-analysis.29(3):136-146. <https://www.ncbi.nlm.nih.gov/pmc/articles/PMC8183542/pdf/YJMT_29_1822618.pdf>.

275. Li D, Mo ZM, Zhang RW, Chang MM, Yang BB, Tang SJ. Is fire needle superior to Western medication for herpes zoster? A systematic review and meta-analysis.17(5):312-320.

276. Li DZ, Zhou Y, Yang YN, et al. Acupuncture for Essential Hypertension: A Meta-Analysis of Randomized Sham-Controlled Clinical Trials. 2014;2014.

277. Li HJ, Schlaeger JM, Jang MK, et al. Acupuncture Improves Multiple Treatment-Related Symptoms in Breast Cancer Survivors: A Systematic Review and Meta-Analysis.27(12):1084-1097. <https://www.liebertpub.com/doi/10.1089/acm.2021.0133?url_ver=Z39.88-2003&rfr_id=ori:rid:crossref.org&rfr_dat=cr_pub%3dpubmed>

https://www.liebertpub.com/doi/pdf/10.1089/acm.2021.0133?download=true.

278. Li J, Yang J, Wu S, Wang MR, Zhu JM. EFFECTS OF ACUPUNCTURE ON RHEUMATOID ARTHRITIS: A SYSTEMATIC REVIEW AND META-ANALYSIS. 2016;13(2):61-71.

279. Li JL, Rong S, Zhou Z, et al. The Efficacy and Safety of Acupuncture for Treating Osteoporotic Vertebral Compression Fracture- (OVCF-) Induced Pain: A Systematic Review and Meta-Analysis of Randomized Clinical Trials. 2021;2021:8574621. <https://www.ncbi.nlm.nih.gov/pmc/articles/PMC8494589/pdf/ECAM2021-8574621.pdf>.

280. Li K, Giustini D, Seely D. A systematic review of acupuncture for chemotherapy-induced peripheral neuropathy.26(2):e147-e154. <https://www.ncbi.nlm.nih.gov/pmc/articles/PMC6476456/pdf/conc-26-e147.pdf>.

281. Li L, Zhang H, Meng SQ, Qian HZ. An updated meta-analysis of the efficacy and safety of acupuncture treatment for cerebral infarction. 2014;9(12):e114057. <https://www.ncbi.nlm.nih.gov/pmc/articles/PMC4250085/pdf/pone.0114057.pdf>.

282. Li LX, Zhang MM, Zhang Y, He J. Acupuncture for cerebral palsy: A meta-analysis of randomized controlled trials.13(6):1107-1117. <https://www.ncbi.nlm.nih.gov/pmc/articles/PMC6022466/pdf/NRR-13-1107.pdf>.

283. Li M, Liu Y, Wang H, Zheng S, Deng Y, Li Y. The Effects of Acupuncture on Pregnancy Outcomes of Recurrent Implantation Failure: A Systematic Review and Meta-Analysis. 2021;2021:6661235. <https://www.ncbi.nlm.nih.gov/pmc/articles/PMC7878089/pdf/ECAM2021-6661235.pdf>.

284. Li P, Qiu T, Qin C. Efficacy of Acupuncture for Bell's Palsy: A Systematic Review and Meta-Analysis of Randomized Controlled Trials. 2015;10(5):e0121880. <https://www.ncbi.nlm.nih.gov/pmc/articles/PMC4431843/pdf/pone.0121880.pdf>.

285. Li S, Zhong W, Peng W, Jiang G. Effectiveness of acupuncture in postpartum depression: a systematic review and meta-analysis.36(5):295-301.

286. Li SQ, Chen JR, Liu ML, Wang YP, Zhou X, Sun X. Effect and Safety of Acupuncture for Type 2 Diabetes Mellitus: A Systematic Review and Meta-analysis of 21 Randomised Controlled Trials %J CHINESE JOURNAL OF INTEGRATIVE MEDICINE.

287. Li W, Li Z, Zhang H, Wang Y, Chen H, Xiong L. Acupoint Catgut Embedding for Insomnia: A Meta-Analysis of Randomized Controlled Trials. 2020;2020:5450824. <https://www.ncbi.nlm.nih.gov/pmc/articles/PMC7665919/pdf/ECAM2020-5450824.pdf>.

288. Li X, Jia HX, Yin DQ, Zhang ZJ. Acupuncture for metabolic syndrome: systematic review and meta-analysis.39(4):253-263.

289. Li XR, Zhang QX, Liu M, et al. Catgut implantation at acupoints for allergic rhinitis: a systematic review.20(3):235-240. <https://link.springer.com/content/pdf/10.1007/s11655-014-1748-z.pdf>.

290. Lian WL, Pan MQ, Zhou DH, Zhang ZJ. Effectiveness of acupuncture for palliative care in cancer patients: a systematic review.20(2):136-147. <https://link.springer.com/content/pdf/10.1007/s11655-013-1439-1.pdf>.

291. Liang S, Huang KY, Xu YT, Sun YN. Acupuncture for chloasma: A systematic review and meta-analysis of randomized controlled trials.14:37-45.

292. Lim B, Manheimer E, Lao L, et al. Acupuncture for treatment of irritable bowel syndrome. (4):Cd005111.

293. Lim CED, Ng RWC, Cheng NCL, Zhang GS, Chen H. Acupuncture for polycystic ovarian syndrome. 2019(7). <http://dx.doi.org/10.1002/14651858.CD007689.pub4>.

294. Lin JG, Chan YY, Chen YH. Acupuncture for the treatment of opiate addiction. 2012;2012. <https://www.embase.com/search/results?subaction=viewrecord&id=L364431642&from=export> <http://dx.doi.org/10.1155/2012/739045>.

295. Liu AF, Gong SW, Chen JX, Zhai JB. Efficacy and Safety of Acupuncture Therapy for Patients with Acute Ankle Sprain: A Systematic Review and Meta-Analysis of Randomized Controlled Trials. 2020;2020:9109531. <https://www.ncbi.nlm.nih.gov/pmc/articles/PMC7585670/pdf/ECAM2020-9109531.pdf>.

296. Liu AJ, Li JH, Li HQ, et al. Electroacupuncture for Acute Ischemic Stroke: A Meta-Analysis of Randomized Controlled Trials. 2015;43(8):1541-1566. <https://www.worldscientific.com/doi/10.1142/S0192415X15500883?url_ver=Z39.88-2003&rfr_id=ori:rid:crossref.org&rfr_dat=cr_pub%3dpubmed>.

297. Liu C, Li T, Wang Z, Zhou R, Zhuang L. Scalp acupuncture treatment for children's autism spectrum disorders: A systematic review and meta-analysis.98(13):e14880. <https://www.ncbi.nlm.nih.gov/pmc/articles/PMC6456017/pdf/medi-98-e14880.pdf>.

298. Liu F, Han X, Li Y, Yu S. Acupuncture in the treatment of tinnitus: a systematic review and meta-analysis.273(2):285-294. <https://link.springer.com/content/pdf/10.1007/s00405-014-3341-7.pdf>.

299. Liu L, Pan L, Tian M, et al. Comparison of Efficacy between Acupuncture Therapies in Improving Sacroiliac Joint Malposition: A Systematic Review and Meta-Analysis. 2022;2022:9485056. <https://www.ncbi.nlm.nih.gov/pmc/articles/PMC8766180/pdf/BMRI2022-9485056.pdf>.

300. Liu P, Deng L, Lv Y, Xu H, Zhao H, Chen X. Single acupuncture treatment can reduce the level of uric acid and alleviating pain in gouty arthritis, a meta-analysis. 2021;46(2):147-158. <https://www.embase.com/search/results?subaction=viewrecord&id=L2011936688&from=export> <http://dx.doi.org/10.3727/036012921X16164310686815>.

301. Liu SN, Zhang CS, Cai YY, et al. Acupuncture for Post-stroke Shoulder-Hand Syndrome: A Systematic Review and Meta-Analysis.10.

302. Liu W, Rao C, Du Y, Zhang L, Yang J. The effectiveness and safety of manual acupuncture therapy in patients with poststroke cognitive impairment: A Meta-analysis. 2020;2020. <https://www.embase.com/search/results?subaction=viewrecord&id=L2010321427&from=export> <http://dx.doi.org/10.1155/2020/8890521>.

303. Liu X, Lu J, Wang G, et al. Acupuncture for Arthralgia Induced by Aromatase Inhibitors in Patients with Breast Cancer: A Systematic Review and Meta-analysis.20:1534735420980811. <https://www.ncbi.nlm.nih.gov/pmc/articles/PMC7883140/pdf/10.1177_1534735420980811.pdf>.

304. Liu XJ, Shi WY, Liu ZF, et al. Effects of acupuncture on Luteinized Unruptured Follicle Syndrome: A meta-analysis of randomized controlled trials.49.

305. Liu XL, Tan JY, Molassiotis A, Suen LKP, Shi Y. Acupuncture-Point Stimulation for Postoperative Pain Control: A Systematic Review and Meta-Analysis of Randomized Controlled Trials. 2015;2015.

306. Liu Y, Fan HY, Hu JQ, Chen M, Chen J. Effectiveness and safety of acupuncture for insulin resistance in women with polycystic ovary syndrome: A systematic review and meta-analysis. In:2022.

307. Liu Y, Meng HY, Khurwolah MR, et al. Acupuncture therapy for the treatment of stable angina pectoris: An updated meta-analysis of randomized controlled trials.34:247-253. <https://www.sciencedirect.com/science/article/pii/S1744388118302780?via%3Dihub>.

308. Liu Y, Wu LQ, Yao SQ, Wu CX, Lu LM, Yi W. Acupuncture for infertile women without undergoing assisted reproductive techniques (ART) A systematic review and meta-analysis.98(29).

309. Liu YH, Dong GT, Ye Y, et al. Effectiveness of Acupuncture for Early Recovery of Bowel Function in Cancer: A Systematic Review and Meta-Analysis. 2017;2017:2504021. <https://www.ncbi.nlm.nih.gov/pmc/articles/PMC5750515/pdf/ECAM2017-2504021.pdf>.

310. Llurda-Almuzara L, Labata-Lezaun N, Meca-Rivera T, et al. Is Dry Needling Effective for the Management of Plantar Heel Pain or Plantar Fasciitis? An Updated Systematic Review and Meta-Analysis.22(7):1630-1641. <https://academic.oup.com/painmedicine/article-abstract/22/7/1630/6184874?redirectedFrom=fulltext>

311. Long Z, Chen H, Yu S, Wang X, Liu Z. Effect of Acupuncture for Mixed Urinary Incontinence in Women: A Systematic Review. 2022;10:827853. <https://www.ncbi.nlm.nih.gov/pmc/articles/PMC8971660/pdf/fpubh-10-827853.pdf>.

312. Lu H, Zheng C, Zhong Y, Cheng L, Zhou Y. Effectiveness of Acupuncture in the Treatment of Hyperemesis Gravidarum: A Systematic Review and Meta-Analysis. 2021;2021:2731446. <https://www.ncbi.nlm.nih.gov/pmc/articles/PMC8337134/pdf/ECAM2021-2731446.pdf>.

313. Lu HL, Chang CM, Hsieh PC, Wang JC, Kung YY. The effects of acupuncture and related techniques on patients with rheumatoid arthritis: A systematic review and meta-analysis.85(3):388-400.

314. Lu W, Hu D, Dean-Clower E, et al. Acupuncture for chemotherapy-induced leukopenia: exploratory meta-analysis of randomized controlled trials.5(1):1-10.

315. Lv ZT, Jiang WX, Huang JM, Zhang JM, Chen AM. The Clinical Effect of Acupuncture in the Treatment of Obstructive Sleep Apnea: A Systematic Review and Meta-Analysis of Randomized Controlled Trials. 2016;2016:8792167. <https://www.ncbi.nlm.nih.gov/pmc/articles/PMC4834396/pdf/ECAM2016-8792167.pdf>.

316. Lv ZT, Li ZQ, Zhou X, Ma WW, Zhang JM, Chen AM. Acupuncture Versus Non-Steroidal Anti-Inflammatory Drugs for Treatment of Chondromalacia Patellae: A Systematic Review and Meta-Analysis of Randomized Controlled Trials. 2016;23(6):344-350.

317. Ma H, Quan XH, Li JW, Wang B, Chen XH, Ieee. Acupuncture for polycystic ovary syndrome: A systematic review and meta-analysis. 2014 %J 2014 IEEE WORKSHOP ON ELECTRONICS, COMPUTER AND APPLICATIONS, 2014.

318. Ma R, Luo D, Liu Y, et al. Acupuncture for Generalized Anxiety Disorder: A Systematic Review. J 2016 8TH INTERNATIONAL CONFERENCE ON INFORMATION TECHNOLOGY IN MEDICINE AND EDUCATION (ITME), 2016.

319. Mak TC, Chen HY, Cho WC. Acupuncture for overactive bladder in adults: a systematic review and meta-analysis.37(6):321-331.

320. ziuk K, Liu Y, Adams D, Vohra S. Acupuncture for cerebral palsy. 2012;17(2):85-90. <https://www.embase.com/search/results?subaction=viewrecord&id=L364818189&from=export> <http://dx.doi.org/10.1111/j.2042-7166.2012.01156.x>.

321. Manheimer E, Cheng K, Linde K, et al. Acupuncture for peripheral joint osteoarthritis. 2010(1). <http://dx.doi.org/10.1002/14651858.CD001977.pub2>.

322. Manheimer E, Cheng K, Wiel, et al. Acupuncture for treatment of irritable bowel syndrome.5(5):Cd005111.

323. Manheimer E, Cheng K, Wiel, et al. Acupuncture for hip osteoarthritis. 2018(5). <http://dx.doi.org/10.1002/14651858.CD013010>.

324. Manheimer E, Linde K, Lao LX, Bouter LM, Berman BM. Meta-analysis: Acupuncture for osteoarthritis of the knee.146(12):868-877.

325. Manheimer E, van der Windt D, Cheng K, et al. The effects of acupuncture on rates of clinical pregnancy among women undergoing in vitro fertilization: a systematic review and meta-analysis.19(6):696-713. <https://www.ncbi.nlm.nih.gov/pmc/articles/PMC3796945/pdf/dmt026.pdf>.

326. Manheimer E, Wiel, LS, et al. Acupuncture for irritable bowel syndrome: systematic review and meta-analysis.107(6):835-847; quiz 848. <https://www.ncbi.nlm.nih.gov/pmc/articles/PMC3671917/pdf/nihms-472358.pdf>.

327. Manheimer E, Zhang G, Udoff L, et al. Effects of acupuncture on rates of pregnancy and live birth among women undergoing in vitro fertilisation: systematic review and meta-analysis.336(7643):545-549. <https://www.ncbi.nlm.nih.gov/pmc/articles/PMC2265327/pdf/bmj-336-7643-res-00545-el.pdf>.

328. Mansu SSY, Liang HY, Parker S, et al. Acupuncture for Acne Vulgaris: A Systematic Review and Meta-Analysis. 2018;2018.

329. Mao X, Guo S, Ni W, et al. Electroacupuncture for the treatment of functional dyspepsia: A systematic review and meta-analysis.99(45):e23014. <https://www.ncbi.nlm.nih.gov/pmc/articles/PMC7647594/pdf/medi-99-e23014.pdf>.

330. Mills EJ, Wu P, Gagnier J, Ebbert JO. Efficacy of acupuncture for cocaine dependence: A systematic review and meta-analysis. 2005;2. <https://www.embase.com/search/results?subaction=viewrecord&id=L40472010&from=export> <http://dx.doi.org/10.1186/1477-7517-2-4>.

331. Moon TW, Posadzki P, Choi TY, et al. Acupuncture for treating whiplash associated disorder: a systematic review of randomised clinical trials. 2014;2014:870271. <https://www.ncbi.nlm.nih.gov/pmc/articles/PMC4034516/pdf/ECAM2014-870271.pdf>.

332. Murakami M, Fox L, Dijkers MP. Ear Acupuncture for Immediate Pain Relief-A Systematic Review and Meta-Analysis of Randomized Controlled Trials.18(3):551-564. <https://watermark.silverchair.com/pnw215.pdf?token=AQECAHi208BE49Ooan9kkhW_Ercy7Dm3ZL_9Cf3qfKAc485ysgAAAt8wggLbBgkqhkiG9w0BBwagggLMMIICyAIBADCCAsEGCSqGSIb3DQEHATAeBglghkgBZQMEAS4wEQQMCOj3HzhAcgT1rIm5AgEQgIICkp2s3QnSYRXCoJG1skxHLM0fXXiF4Yg6Ap7nW-nBHbGntiIg-Ph1MC_RkLLwNG-4UklZ3zbISLDA7WjJlRFvcBu9waq9s2uzSni_QQD6QJd502jZQa0ucq_bAVoQnaNr376umiLcizSq5j5HbMfNF2F1cYauQTbw-RCAQrPcHM7ujR4HRP35W_6Nz4ko3aWnP8WTxJKC2BIMOk822IPmLfHoYx6pD6W1yfGln5KDOwE-gt-16HtxVO9HfVDSGkSUdyP1Wd7i09oG4XkVVeP3oKhSHVlvjeJA9QoiCfTfHYoDhGdglGMQ5DeqeTMxZqAtiRn-LKLLllswWb3i8tVKAFBhM3gxMlTBu991daWVI0t7Y3w9ZISH4BMye9bOd4WeDWKj7K84TEegMGK3CWbLwzoqTqBLZohogJ72p-xVy9tc6QbkuJL413mNg5zn2N_hoCSxiCZCqtgDFmYFjZ7ss70_FELZ6_rtBwZdCc-HnRr3mmuxR-GIuhzRMr9x6Fxy-RSeoNyEn6sSxyKV9fd9XEvRwmBrmj3_n9aWuhXIq3tavzwQXUBYnXK-_Ko-EjUiwvKfSf0M7W6fPL2Zb4pXYtKw8zrv5tDW6Vm46w3R0RgIppajzUwldQPUMtMyxC4G4bwqjlMZDTxHIrqs034y5xGFHniU7T6PqIgG0gTDSqjVZzTdykjV_YguPS2OHF8NEmKhYdXZQrGyVnmquo9-JXIn3cf4bb_JLUds1Sz2Df23PuYJGqGcNxcPJPlajNvLUDgqLuCZmWq9b6-qOK2cLmjJQFUL984k8N3leR4Bz6NoFCN4Lxt5wONCQ_UA6Y7JqYFPkPExQQOMC9YUcqOpSWo9J5cb2xS3DXiplAuQRflh8OU>.

333. Naguit N, Laeeq S, Jakkoju R, et al. Is Acupuncture Safe and Effective Treatment for Migraine? A Systematic Review of Randomized Controlled Trials.14(1).

334. Namazi N, Khodamoradi K, Larijani B, Ayati MH. Is laser acupuncture an effective complementary therapy for obesity management? A systematic review of clinical trials.35(6):452-459.

335. Navarro-Santana MJ, Sanchez-Infante J, Fernández-de-Las-Peñas C, et al. Effectiveness of Dry Needling for Myofascial Trigger Points Associated with Neck Pain Symptoms: An Updated Systematic Review and Meta-Analysis.9(10).

336. Navarro-Santana MJ, Sanchez-Infante J, Gómez-Chiguano GF, et al. Dry Needling Versus Trigger Point Injection for Neck Pain Symptoms Associated with Myofascial Trigger Points: A Systematic Review and Meta-Analysis.23(3):515-525. <https://watermark.silverchair.com/pnab188.pdf?token=AQECAHi208BE49Ooan9kkhW_Ercy7Dm3ZL_9Cf3qfKAc485ysgAAAuEwggLdBgkqhkiG9w0BBwagggLOMIICygIBADCCAsMGCSqGSIb3DQEHATAeBglghkgBZQMEAS4wEQQMKr9e2udYIyQv8MznAgEQgIIClMhzNbKiJBzg0hEUhf_dF7iyYf0bTdFhsZklZdZ-dG4N9cfi4yMnAMfawqc44APbzRNdBj4uJR9sS7nzZRqwPbGSW_yZrjjpHP3QqLdms_oYw1oRQzuC32sN5s-rmmhVth23Tr76rnFJn0vhYf50lHgEpq_yEnfXj9vKIisbrqfTvGEUPMCvsQPZotn5XK86Z2loquoP7t6kxF9VT7fK83CKDPWMWnav3HKL7_Z2V7PbQow3mV6cVdL1KWUsGS1pHF3bwY98PLzyjnZhblKlflCOzoxoG-XDTiY5JSp73SqOqvf4Hre5c6X0jfylSWla9X2ngORFQhHrzh_fnuq-nL_YzleTw1N_YSSZGHhmyirnBPbKZzcvWcgQtmFZi1M8skoCLJtrfRgmpH03dkC8YCr-Rh3w11brCgO-0UdaFbjsp8mxIf2qZ2bQDldKcMUfmDSucUWMDQoeyh0cS73oj93fNinFb1g28CSlAG6GWrmC1ree9-j1zlrWH0msEHL0e3wFCX5ksOvzM64w96TLe-NvFhZhtHMhQamBb2pjLaWDRR_eWzwy-8rUUj6o4mBSkxP0oiTbpmlau2D4su1-i5TGYe6rcMet2TWxpXudh0a6H6qgcTjiAjR3Kxe1ZkgmxMABMRRxBdsDrlwh9dOctiMtvMasyMseoCHvoZkjmrGTCUkXP2oe01DBSG3OiybMTRlwCuf_dwnanmJOYt9byuH8bS6xIBgtKqzAi58xMD-ERIFeMp_twkuAln3gUJcON9vps40ggrmsjVVBYszJptqKjShR3S7OQ7I2Pm6CsjVbeqE42MFlbivjWTc1BZ7oKqm-GWmtgqoR4es-JFB4hGV1xfK6LFus3nmA6-0Vqi3s0Hwe3w>.

337. Navarro-Santana MJ, Sanchez-Infante J, Gómez-Chiguano GF, et al. Effects of trigger point dry needling on lateral epicondylalgia of musculoskeletal origin: a systematic review and meta-analysis.34(11):1327-1340.

338. Ni XX, Tian T, Chen D, et al. Acupuncture for Radiation-Induced Xerostomia in Cancer Patients: A Systematic Review and Meta-Analysis.19.

339. Noh H, Kwon S, Cho SY, et al. Effectiveness and safety of acupuncture in the treatment of Parkinson's disease: A systematic review and meta-analysis of randomized controlled trials.34:86-103. <https://www.sciencedirect.com/science/article/pii/S0965229917303357?via%3Dihub>.

340. O'Sullivan EM, Higginson IJ. Clinical effectiveness and safety of acupuncture in the treatment of irradiation-induced xerostomia in patients with head and neck cancer: a systematic review.28(4):191-199.

341. Ou L, Shen Z, Zhang T, et al. Electroacupuncture for the Prevention of Postoperative Cognitive Dysfunction Among Older Adults Undergoing Hip and Knee Arthroplasty: A Systematic Review and Meta-Analysis of Randomized Controlled Trials. 2021;8:778474. <https://www.ncbi.nlm.nih.gov/pmc/articles/PMC8764307/pdf/fmed-08-778474.pdf>.

342. Paley CA, Tashani OA, Bagnall AM, Johnson MI. A Cochrane systematic review of acupuncture for cancer pain in adults.1(1):51-55. <https://spcare.bmj.com/content/bmjspcare/1/1/51.full.pdf>.

343. Pan H, Jin R, Li M, Liu Z, Xie Q, Wang P. The Effectiveness of Acupuncture for Osteoporosis: A Systematic Review and Meta-Analysis. 2018;46(3):489-513. <https://www.worldscientific.com/doi/10.1142/S0192415X18500258?url_ver=Z39.88-2003&rfr_id=ori:rid:crossref.org&rfr_dat=cr_pub%3dpubmed>.

344. Pan YQ, Yang KH, Shi X, et al. Clinical Benefits of Acupuncture for the Reduction of Hormone Therapy-Related Side Effects in Breast Cancer Patients: A Systematic Review.17(3):602-618. <https://www.ncbi.nlm.nih.gov/pmc/articles/PMC6142070/pdf/10.1177_1534735418786801.pdf>.

345. Pang B, Jiang T, Du YH, et al. Acupuncture for Functional Dyspepsia: What Strength Does It Have? A Systematic Review and Meta-Analysis of Randomized Controlled Trials. 2016;2016:3862916. <https://www.ncbi.nlm.nih.gov/pmc/articles/PMC5227170/pdf/ECAM2016-3862916.pdf>.

346. Park J, Hahn S, Park JY, Park HJ, Lee H. Acupuncture for ankle sprain: systematic review and meta-analysis.13:55. <https://www.ncbi.nlm.nih.gov/pmc/articles/PMC3606608/pdf/1472-6882-13-55.pdf>.

347. Park J, Hopwood V, White AR, Ernst E. Effectiveness of acupuncture for stroke: a systematic review.248(7):558-563. <https://link.springer.com/content/pdf/10.1007/s004150170132.pdf>.

348. Park J, Sohn Y, White AR, Lee H. The safety of acupuncture during pregnancy: a systematic review.32(3):257-266. <https://www.ncbi.nlm.nih.gov/pmc/articles/PMC4112450/pdf/acupmed-2013-010480.pdf>.

349. Park JY, Kim YK, Kim SY, et al. Acupuncture modulates brain neural activity in patients: a systematic review and meta-analysis.17(2):111-126.

350. Park KS, Park KI, Suh HS, Hwang DS, Jang JB, Lee JM. The efficacy and safety of acupuncture on serum leptin levels in obese patients: A systematic review and meta-analysis.11:45-52.

351. Park S, Lyu YR, Park SJ, Oh MS, Jung IC, Lee EJ. Electroacupuncture for post-thoracotomy pain: A systematic review and meta-analysis. 2021;16(7):e0254093. <https://www.ncbi.nlm.nih.gov/pmc/articles/PMC8263274/pdf/pone.0254093.pdf>.

352. Park YJ, Lee JM. Effect of Acupuncture Intervention and Manipulation Types on Poststroke Dysarthria: A Systematic Review and Meta-Analysis. 2020;2020:4981945. <https://www.ncbi.nlm.nih.gov/pmc/articles/PMC7509576/pdf/ECAM2020-4981945.pdf>.

353. Posadzki P, Moon TW, Choi TY, Park TY, Lee MS, Ernst E. Acupuncture for cancer-related fatigue: a systematic review of randomized clinical trials.21(7):2067-2073. <https://link.springer.com/content/pdf/10.1007/s00520-013-1765-z.pdf>.

354. Posadzki P, Zhang JH, Lee MS, Ernst E. Acupuncture for Chronic Nonbacterial Prostatitis/Chronic Pelvic Pain Syndrome: A Systematic Review.33(1):15-21.

355. Pourahmadi M, Dommerholt J, Fernández-de-Las-Peñas C, et al. Dry Needling for the Treatment of Tension-Type, Cervicogenic, or Migraine Headaches: A Systematic Review and Meta-Analysis.101(5).

356. Qin X, Coyle ME, Yang L, et al. Acupuncture for recurrent urinary tract infection in women: a systematic review and meta-analysis.127(12):1459-1468. <https://obgyn.onlinelibrary.wiley.com/doi/10.1111/1471-0528.16315>.

357. Qin Z, Liu X, Wu J, Zhai Y, Liu Z. Effectiveness of Acupuncture for Treating Sciatica: A Systematic Review and Meta-Analysis. 2015;2015:425108. <https://www.ncbi.nlm.nih.gov/pmc/articles/PMC4631886/pdf/ECAM2015-425108.pdf>.

358. Qin ZS, Wu JN, Zhou J, Liu ZS. Systematic Review of Acupuncture for Chronic Prostatitis/Chronic Pelvic Pain Syndrome.95(11).

359. Qiu X, Gao YC, Zhang ZX, Cheng SJ, Zhang SM. Fire Acupuncture versus conventional acupuncture to treat spasticity after stroke: A systematic review and meta-analysis.16(4).

360. Qu F, Wu Y, Hu XY, et al. The effects of acupuncture on polycystic ovary syndrome: A systematic review and meta-analysis.8(1):12-18.

361. Rahou-El-Bachiri Y, Navarro-Santana MJ, Gómez-Chiguano GF, et al. Effects of Trigger Point Dry Needling for the Management of Knee Pain Syndromes: A Systematic Review and Meta-Analysis.9(7).

362. Rathbone J, Xia J. Acupuncture for schizophrenia. 2005(4).

363. Roberts J, Huissoon A, Dretzke J, Wang D, Hyde C. A systematic review of the clinical effectiveness of acupuncture for allergic rhinitis.8:13. <https://www.ncbi.nlm.nih.gov/pmc/articles/PMC2386775/pdf/1472-6882-8-13.pdf>.

364. Seo SY, Lee KB, Shin JS, et al. Effectiveness of Acupuncture and Electroacupuncture for Chronic Neck Pain: A Systematic Review and Meta-Analysis. 2017;45(8):1573-1595. <https://www.worldscientific.com/doi/10.1142/S0192415X17500859?url_ver=Z39.88-2003&rfr_id=ori:rid:crossref.org&rfr_dat=cr_pub%3dpubmed>.

365. Shen FJ, Xu J, Zhan YJ, Fu QH, Pei J. Acupuncture for migraine: A systematic review and meta-analysis.29(1):7-14.

366. Shen L, Lee JH, Joo JC, Park SJ, Song YS. Bee Venom Acupuncture for Shoulder Pain: A Systematic Review and Meta-analysis of Randomized Controlled Trials.23(2):44-53. <https://www.ncbi.nlm.nih.gov/pmc/articles/PMC7338706/pdf/2093-6966-v23-n02-044.pdf>.

367. Sheng J, Jin X, Zhu J, Chen Y, Liu X. The Effectiveness of Acupoint Catgut Embedding Therapy for Abdominal Obesity: A Systematic Review and Meta-Analysis. 2019;2019:9714313. <https://www.ncbi.nlm.nih.gov/pmc/articles/PMC6612388/pdf/ECAM2019-9714313.pdf>.

368. Shergis JL, Ni X, Jackson ML, et al. A systematic review of acupuncture for sleep quality in people with insomnia.26:11-20. <https://www.sciencedirect.com/science/article/pii/S0965229916300164?via%3Dihub>.

369. Sim H, Shin BC, Lee MS, Jung A, Lee H, Ernst E. Acupuncture for carpal tunnel syndrome: a systematic review of randomized controlled trials.12(3):307-314.

370. Skjeie H, Skonnord T, Brekke M, et al. Acupuncture treatments for infantile colic: a systematic review and individual patient data meta-analysis of blinding test validated randomised controlled trials.36(1):56-69. <https://www.ncbi.nlm.nih.gov/pmc/articles/PMC5901442/pdf/ipri-36-56.pdf>.

371. Smith CA, Zhu X, He L, Song J. Acupuncture for primary dysmenorrhoea. (1):Cd007854.

372. Sniezek DP, Siddiqui IJ. Acupuncture for Treating Anxiety and Depression in Women: A Clinical Systematic Review.25(3):164-172. <https://www.ncbi.nlm.nih.gov/pmc/articles/PMC3689180/pdf/acu.2012.0900.pdf>.

373. Sorbero ME, Reynolds K, Colaiaco B, et al. Acupuncture for Major Depressive Disorder: A Systematic Review.5(4):7.

374. Southern C, Lloyd C, Liu J, et al. Acupuncture as an intervention to reduce alcohol dependency: a systematic review and meta-analysis. 2016;11:49. <https://www.ncbi.nlm.nih.gov/pmc/articles/PMC5160025/pdf/13020_2016_Article_119.pdf>.

375. Stub T, Alraek T, Liu JP. Acupuncture treatment for depression-A systematic review and meta-analysis.3(4):E253-E264.

376. Su IJ, Liao L, Huang F. A Meta-Analysis of the Effect of Abdominal Acupuncture on Post-Stroke Depression.33(4):269-277.

377. Su X, Qian H, Chen BY, et al. Acupuncture for acute low back pain: a systematic review and meta-analysis.10(4):3924-+. <https://apm.amegroups.com/article/viewFile/66741/pdf>.

378. Sun Y, Gan TJ. Acupuncture for the management of chronic headache: a systematic review.107(6):2038-2047.

379. Sun Y, Gan TJ, Dubose JW, Habib AS. Acupuncture and related techniques for postoperative pain: a systematic review of randomized controlled trials.101(2):151-160. <https://www.sciencedirect.com/science/article/pii/S0007091217342265?via%3Dihub>.

380. Sung SH, Kim D, Park M, et al. Electroacupuncture for Temporomandibular Disorders: A Systematic Review of Randomized Controlled Trials.9(11).

381. Sung SH, Sung ADM, Sung HK, An TEB, Kim KH, Park JK. Acupuncture Treatment for Chronic Pelvic Pain in Women: A Systematic Review and Meta-Analysis of Randomized Controlled Trials. 2018;2018.

382. Sung WS, Goo BH, Kim EJ, et al. Efficacy and safety of thread-embedding acupuncture for lumbar herniated intervertebral disc: A systematic review and meta-analysis.39.

383. Tan J, Meng F, Zhang B, et al. Electroacupuncture for Spinal Cord Injury: A Systematic Review and Meta-Analysis of Randomised Controlled Trials. 2022;2022:8040555. <https://www.ncbi.nlm.nih.gov/pmc/articles/PMC8916891/pdf/ECAM2022-8040555.pdf>.

384. Tang ECH, Hung C, Lo SHS, Chau JPC, Mok VCT, Lau AYL. Acupuncture on vascular cognitive impairment associated with cerebral small vessel disease: A systematic review and meta-analysis of randomized and non-randomized controlled trials. 2022;49. <https://www.embase.com/search/results?subaction=viewrecord&id=L2015735662&from=export> <http://dx.doi.org/10.1016/j.eujim.2021.101403>.

385. Tang HZ, Fan HY, Chen J, et al. Acupuncture for Lateral Epicondylitis: A Systematic Review. 2015;2015.

386. Tang S, Mo Z, Zhang R. Acupuncture for lumbar disc herniation: a systematic review and meta-analysis.36(2):62-70.

387. Thiagarajah AG. How effective is acupuncture for reducing pain due to plantar fasciitis? ;58(2):92-97. <https://www.ncbi.nlm.nih.gov/pmc/articles/PMC5311890/pdf/SMJ-58-92.pdf>.

388. Tong QY, Liu R, Zhang K, Gao Y, Cui GW, Shen WD. Can acupuncture therapy reduce preoperative anxiety? A systematic review and meta-analysis.19(1):20-28. <https://www.sciencedirect.com/science/article/pii/S2095496420301254?via%3Dihub>.

389. Trigkilidas D. Acupuncture therapy for chronic lower back pain: a systematic review.92(7):595-598. <https://www.ncbi.nlm.nih.gov/pmc/articles/PMC3229352/pdf/rcse9207-595.pdf>.

390. Trinh K, Belski N, Zhou FW, Kuhad A, Luk D, Youn E. The Efficacy of Acupuncture on Foot and Ankle for Pain Intensity, Functional Status, and General Quality of Life in Adults: A Systematic Review.33(6):386-395.

391. Trinh K, Zhou F, Belski N, Deng J, Wong CY. The Effect of Acupuncture on Hand and Wrist Pain Intensity, Functional Status, and Quality of Life in Adults: A Systematic Review.34(1):34-48.

392. Trinh KV, Graham N, Gross AR, et al. Acupuncture for neck disorders. (3):Cd004870.

393. Tu M, Jiang Y, Yu J, et al. Acupuncture for treating chronic stable angina pectoris associated anxiety and depression: A systematic review and meta-analysis.45:101484. <https://www.sciencedirect.com/science/article/pii/S1744388121001833?via%3Dihub>.

394. Tu Y, Peng W, Wang J, et al. Acupuncture Therapy on Patients with Flaccid Hemiplegia after Stroke: A Systematic Review and Meta-Analysis.2022.

395. Urroz P, Colagiuri B, Smith CA, Cheema BS. Effect of acute acupuncture treatment on exercise performance and postexercise recovery: a systematic review.19(1):9-16. <https://www.liebertpub.com/doi/pdf/10.1089/acm.2011.0727?download=true>.

396. Valencia-Chulián R, Heredia-Rizo AM, Moral-Munoz JA, Lucena-Anton D, Luque-Moreno C. Dry needling for the management of spasticity, pain, and range of movement in adults after stroke: A systematic review.52:102515. <https://www.sciencedirect.com/science/article/pii/S0965229920305586?via%3Dihub>.

397. Van den Heuvel E, Goossens M, erhaegen H, Sun HX, Buntinx F. Effect of acustimulation on nausea and vomiting and on hyperemesis in pregnancy: a systematic review of Western and Chinese literature.16:13. <https://www.ncbi.nlm.nih.gov/pmc/articles/PMC4711053/pdf/12906_2016_Article_985.pdf>.

398. van den Noort M, Yeo S, Lim S, Lee SH, Staudte H, Bosch P. Acupuncture as Add-On Treatment of the Positive, Negative, and Cognitive Symptoms of Patients with Schizophrenia: A Systematic Review.5(2).

399. von Trott P, Oei SL, Ramsenthaler C. Acupuncture for Breathlessness in Advanced Diseases: A Systematic Review and Meta-analysis.59(2):327-338.e323. <https://www.sciencedirect.com/science/article/pii/S0885392419305275?via%3Dihub>.

400. Wang J, Li J, Yu X, Xie Y. Acupuncture Therapy for Functional Effects and Quality of Life in COPD Patients: A Systematic Review and Meta-Analysis. 2018;2018:3026726. <https://www.ncbi.nlm.nih.gov/pmc/articles/PMC5985111/pdf/BMRI2018-3026726.pdf>.

401. Wang J, Xiong X, Liu W. Acupuncture for essential hypertension.169(5):317-326. <https://www.sciencedirect.com/science/article/pii/S0167527313017282?via%3Dihub>.

402. Wang J, Zhai YB, Wu JN, Zhao ST, Zhou J, Liu ZS. Acupuncture for Chronic Urinary Retention due to Spinal Cord Injury: A Systematic Review. 2016;2016.

403. Wang JH, van Haselen R, Wang M, et al. Acupuncture for smoking cessation: A systematic review and meta-analysis of 24 randomized controlled trials. 2019;17:48. <https://www.ncbi.nlm.nih.gov/pmc/articles/PMC6662782/pdf/TID-17-48.pdf>.

404. Wang L, Peng JL, Qiao FQ, et al. Clinical Randomized Controlled Study of Acupuncture Treatment on Children with Autism Spectrum Disorder (ASD): A Systematic Review and Meta-Analysis. 2021;2021:5549849. <https://www.ncbi.nlm.nih.gov/pmc/articles/PMC8328702/pdf/ECAM2021-5549849.pdf>.

405. Wang L, Xu J, Zhan Y, Pei J. Acupuncture for Obstructive Sleep Apnea (OSA) in Adults: A Systematic Review and Meta-Analysis. 2020;2020:6972327. <https://www.ncbi.nlm.nih.gov/pmc/articles/PMC7079261/pdf/BMRI2020-6972327.pdf>.

406. Wang LQ, Chen Z, Zhang K, et al. Zusanli (ST36) Acupoint Injection for Diabetic Peripheral Neuropathy: A Systematic Review of Randomized Controlled Trials.24(12):1138-1149. <https://www.liebertpub.com/doi/pdf/10.1089/acm.2018.0053?download=true>.

407. Wang M, Gao YH, Xu J, et al. Zusanli (ST36) acupoint injection for preventing postoperative ileus: A systematic review and meta-analysis of randomized clinical trials.23(3):469-483. <https://www.ncbi.nlm.nih.gov/pmc/articles/PMC4909358/pdf/nihms-789296.pdf>.

408. Wang P, Ma X, Huang J, et al. Effect of acupuncture treatment on dysphagia caused by pseudobulbar paralysis after stroke: a systematic review and meta-analysis.

409. Wang R, Li X, Zhou S, Zhang X, Yang K, Li X. Manual acupuncture for myofascial pain syndrome: a systematic review and meta-analysis.35(4):241-250.

410. Wang TT, Liu Y, Ning ZY, Qi R. Efficacy and safety of acupuncture for the treatment of knee osteoarthritis: a systematic review and meta-analysis.18(3):180-190.

411. Wang WH, Jiang RW, Liu NC. Electroacupuncture Is Effective for Peripheral Facial Paralysis: A Meta-Analysis. 2020;2020:5419407. <https://www.ncbi.nlm.nih.gov/pmc/articles/PMC7150689/pdf/ECAM2020-5419407.pdf>.

412. Wang XM, Gong J, Li SC, Han M. Acupuncture Compared with Intramuscular Injection of Neostigmine for Postpartum Urinary Retention: A Systematic Review and Meta-Analysis of Randomized Controlled Trials. 2018;2018:2072091. <https://www.ncbi.nlm.nih.gov/pmc/articles/PMC5976954/pdf/ECAM2018-2072091.pdf>.

413. Wang XP, Zhang DJ, Wei XD, Wang JP, Zhang DZ. Acupuncture for the relief of hot flashes in breast cancer patients: A systematic review and meta-analysis of randomized controlled trials and observational studies.14:S600-s608. <https://www.cancerjournal.net/article.asp?issn=0973-1482;year=2018;volume=14;issue=10;spage=600;epage=608;aulast=Wang>.

414. Wang Y, Li W, Peng W, Zhou J, Liu Z. Acupuncture for postherpetic neuralgia: Systematic review and meta-analysis.97(34):e11986. <https://www.ncbi.nlm.nih.gov/pmc/articles/PMC6113033/pdf/medi-97-e11986.pdf>.

415. Wang Y, Shen J, Wang XM, et al. Scalp acupuncture for acute ischemic stroke: A meta-analysis of randomized controlled trials. 2012;2012. <https://www.embase.com/search/results?subaction=viewrecord&id=L366306962&from=export> <http://dx.doi.org/10.1155/2012/480950>.

416. Wang Y, Zhishun L, Peng W, Zhao J, Liu B. Acupuncture for stress urinary incontinence in adults. 2013(7). <http://dx.doi.org/10.1002/14651858.CD009408.pub2>.

417. Wei X, He L, Liu J, et al. Electroacupuncture for Reflex Sympathetic Dystrophy after Stroke: A Meta-Analysis.28(5):1388-1399. <https://www.sciencedirect.com/science/article/pii/S1052305719300540?via%3Dihub>.

418. Wen X, Li K, Wen H, et al. Acupuncture-Related Therapies for Parkinson's Disease: A Meta-Analysis and Qualitative Review. 2021;13:676827. <https://www.ncbi.nlm.nih.gov/pmc/articles/PMC8282198/pdf/fnagi-13-676827.pdf>.

419. Wong ISY, Ng KF, Tsang HWH. Acupuncture for dysphagia following stroke: A systematic review.4(2):E141-E150.

420. Wong V, Cheuk DKL, Lee S, Chu V. Acupuncture for acute management and rehabilitation of traumatic brain injury. 2011;2011(5). <https://www.embase.com/search/results?subaction=viewrecord&id=L620561987&from=export> <http://dx.doi.org/10.1002/14651858.CD007700.pub2>.

421. Woo HL, Ji HR, Pak YK, et al. The efficacy and safety of acupuncture in women with primary dysmenorrhea: A systematic review and meta-analysis.97(23):e11007. <https://www.ncbi.nlm.nih.gov/pmc/articles/PMC5999465/pdf/medi-97-e11007.pdf>.

422. Wu HM, Tang JL, Lin XP, et al. Acupuncture for stroke rehabilitation. 2006;3:CD004131. <https://www.embase.com/search/results?subaction=viewrecord&id=L44588012&from=export>.

423. Wu IX, Lam VC, Ho RS, et al. Acupuncture and related interventions for carpal tunnel syndrome: systematic review.34(1):34-44.

424. Wu LQ, Chen XK, Liu Y, et al. Role of acupuncture in the treatment of insulin resistance: A systematic review and meta-analysis.37:11-22. <https://www.sciencedirect.com/science/article/pii/S1744388119301288?via%3Dihub>.

425. Xiang A, Cheng K, Shen X, Xu P, Liu S. The Immediate Analgesic Effect of Acupuncture for Pain: A Systematic Review and Meta-Analysis. 2017;2017:3837194. <https://www.ncbi.nlm.nih.gov/pmc/articles/PMC5676441/pdf/ECAM2017-3837194.pdf>.

426. Xiang Y, He JY, Tian HH, Cao BY, Li R. Evidence of efficacy of acupuncture in the management of low back pain: a systematic review and meta-analysis of randomised placebo- or sham-controlled trials.38(1):15-24.

427. Xiao X, Li S, Cao Z, Wang Y. Acupuncture in the treatment of stable chronic obstructive pulmonary disease: A systematic review of randomised controlled trials.32(8):3237-3248.

428. Xie G, Wang T, Tang X, et al. Acupoint Injection for Nonspecific Chronic Low Back Pain: A Systematic Review and Meta-Analysis of Randomized Controlled Studies. 2020;2020:3976068. <https://www.ncbi.nlm.nih.gov/pmc/articles/PMC7641697/pdf/ECAM2020-3976068.pdf>.

429. Xing M, Yan X, Sun X, et al. Fire needle therapy for moderate-severe acne: A PRISMA systematic review and meta-analysis of randomized controlled trials.44:253-260. <https://www.sciencedirect.com/science/article/pii/S0965229918309075?via%3Dihub>.

430. Xiong J, Qi WC, Yang H, et al. Acupuncture Treatment for Cough-Variant Asthma: A Meta-Analysis.2021.

431. Xiong W, Feng X, Liu J, Chen W. Electroacupuncture for treatment of diabetic peripheral neuropathy: A systematic review of randomized controlled trials. 2016;3(1):9-21. <https://www.embase.com/search/results?subaction=viewrecord&id=L612580067&from=export> <http://dx.doi.org/10.1016/j.jtcms.2016.06.002>.

432. Xu G, Lei H, Zhou Y, et al. Acupuncture for Quality of Life of Patients with Defecation Dysfunction after Sphincter Preserving Surgery for Rectal Cancer: A Systematic Review. 2021;2021:7858252. <https://www.ncbi.nlm.nih.gov/pmc/articles/PMC8694980/pdf/ECAM2021-7858252.pdf>.

433. Xuan Y, Zhang H, Liu D, et al. The efficacy and safety of simple-needling for the treatment of primary dysmenorrhea compared with ibuprofen: A systematic review and meta-analysis.101(7):e28919. <https://docserver.ingentaconnect.com/deliver/connect/wk/00257974/v101n7/s58.pdf?expires=1651225498&id=0000&titleid=99002871&checksum=256199FDA0E2110A4CC8D153D3D2B3F9&host=https://www.ingentaconnect.com>.

434. Xuan YC, Huang H, Huang YY, Liu DY, Hu XW, Geng LL. The Efficacy and Safety of Simple-Needling Therapy for Treating Ankylosing Spondylitis: A Systematic Review and Meta-Analysis of Randomized Controlled Trials.2020.

435. Xue P, Wang L, Han M, et al. Acupoint injection for asthma: Systematic review of randomized controlled trials. 2016;3(1):22-36. <https://www.embase.com/search/results?subaction=viewrecord&id=L612580048&from=export> <http://dx.doi.org/10.1016/j.jtcms.2016.03.005>.

436. Yan B, Zhu S, Wang Y, Da G, Tian G. Effect of Acupuncture on Chronic Pain with Depression: A Systematic Review. 2020;2020:7479459. <https://www.ncbi.nlm.nih.gov/pmc/articles/PMC7334776/pdf/ECAM2020-7479459.pdf>.

437. Yang B, Yi G, Hong W, et al. Efficacy of acupuncture on fibromyalgia syndrome: a meta-analysis.34(4):381-391.

438. Yang C, Lv T, Yu TY, Wong S, Lu MQ, Li YZ. Acupuncture at Tiaokou (ST38) for Shoulder Adhesive Capsulitis: What Strengths Does It Have? A Systematic Review and Meta-Analysis of Randomized Controlled Trials. 2018;2018. <https://www.embase.com/search/results?subaction=viewrecord&id=L622060455&from=export> <http://dx.doi.org/10.1155/2018/4197659>.

439. Yang J. Acupuncture treatment for post-stroke insomnia: A systematic review and meta-analysis of randomized controlled trials.44:101396. <https://www.sciencedirect.com/science/article/pii/S1744388121000955?via%3Dihub>.

440. Yang J, Chen J, Yang M, et al. Acupuncture for hypertension. 2018(11). <http://dx.doi.org/10.1002/14651858.CD008821.pub2>.

441. Yang J, Ganesh R, Wu Q, et al. Battlefield Acupuncture for Adult Pain: A Systematic Review and Meta-Analysis of Randomized Controlled Trials. 2021;49(1):25-40. <https://www.worldscientific.com/doi/pdf/10.1142/S0192415X21500026?download=true>.

442. Yang L, Yang Z, Yu H, Song H. Acupuncture therapy is more effective than artificial tears for dry eye syndrome: evidence based on a meta-analysis. 2015;2015:143858. <https://www.ncbi.nlm.nih.gov/pmc/articles/PMC4415668/pdf/ECAM2015-143858.pdf>.

443. Yang M, Du T, Long H, Sun M, Liang F, Lao L. Acupuncture for menstrual migraine: a systematic review.

444. Yang MX, Li XM, Liu SH, et al. Meta-analysis of acupuncture for relieving non-organic dyspeptic symptoms suggestive of diabetic gastroparesis.13.

445. Yang T, Zhao J, Guo Q, Wang Y, Si G. Acupoint injection treatment for non-dialysis dependent chronic kidney disease: A meta-analysis of randomized controlled trials.99(51):e23306. <https://www.ncbi.nlm.nih.gov/pmc/articles/PMC7748216/pdf/medi-99-e23306.pdf>.

446. Yang XY, Yang NB, Huang FF, Ren S, Li ZJ. Effectiveness of acupuncture on anxiety disorder: a systematic review and meta-analysis of randomised controlled trials.20(1):9. <https://www.ncbi.nlm.nih.gov/pmc/articles/PMC7847562/pdf/12991_2021_Article_327.pdf>.

447. Yang Y, Wen J, Hong J. The Effects of Auricular Therapy for Cancer Pain: A Systematic Review and Meta-Analysis. 2020;2020:1618767. <https://www.ncbi.nlm.nih.gov/pmc/articles/PMC7267873/pdf/ECAM2020-1618767.pdf>.

448. Yao JP, He ZQ, Chen Y, et al. Acupuncture and weight loss in Asians A PRISMA-compliant systematic review and meta-analysis.98(33).

449. Yao Q, Li S, Liu X, Qin Z, Liu Z. The Effectiveness and Safety of Acupuncture for Patients with Chronic Urticaria: A Systematic Review. 2016;2016:5191729. <https://www.ncbi.nlm.nih.gov/pmc/articles/PMC4897793/pdf/BMRI2016-5191729.pdf>.

450. Ye JY, He YJ, Zhan MJ, Qu F. Effects of acupuncture on the relief of anxiety and/or depression during in vitro fertilization: A systematic review and meta-analysis.42.

451. Ye Q, Xie Y, Shi J, Xu Z, Ou A, Xu N. Systematic Review on Acupuncture for Treatment of Dysphagia after Stroke. 2017;2017:6421852. <https://www.ncbi.nlm.nih.gov/pmc/articles/PMC5568619/pdf/ECAM2017-6421852.pdf>.

452. Yeung WF, Chung KF, Leung YK, Zhang SP, Law AC. Traditional needle acupuncture treatment for insomnia: a systematic review of randomized controlled trials.10(7):694-704. <https://www.sciencedirect.com/science/article/pii/S1389945708003651?via%3Dihub>.

453. Yi HM, Han YJ, Li MX, Wang J, Yang LP, Iop. Scalp acupuncture for Autism spectrum disorder: a systematic review. 2020 %J 2019 5TH INTERNATIONAL CONFERENCE ON ENVIRONMENTAL SCIENCE AND MATERIAL APPLICATION, 2020.

454. Yin ZH, Xiao QW, Xu GX, et al. Acupuncture for the Postcholecystectomy Syndrome: A Systematic Review and Meta-Analysis.2020.

455. You F, Ruan L, Zeng L, Zhang Y. Efficacy and safety of acupuncture for the treatment of oligoasthenozoospermia: A systematic review. 2019:e13415. <https://www.embase.com/search/results?subaction=viewrecord&id=L629727217&from=export> <http://dx.doi.org/10.1111/and.13415>.

456. You YN, Song MY, Park GC, et al. Meta-analysis on randomized controlled trials for scalp acupuncture treatment of stroke: A systematic review.38(4):465-479.

457. Yu C, Ji K, Cao H, et al. Effectiveness of acupuncture for angina pectoris: a systematic review of randomized controlled trials.15:90. <https://www.ncbi.nlm.nih.gov/pmc/articles/PMC4426772/pdf/12906_2015_Article_586.pdf>.

458. Yu CC, Ma CY, Xiong Y, et al. Effectiveness of acupoint catgut embedding therapy for polycystic ovary syndrome: a systematic review and meta-analysis.27(4):41-51.

459. Yu J, Ye Y, Liu J, Wang Y, Peng W, Liu Z. Acupuncture for Tourette Syndrome: A Systematic Review. 2016;2016:1834646. <https://www.ncbi.nlm.nih.gov/pmc/articles/PMC5048029/pdf/ECAM2016-1834646.pdf>.

460. Yu S, Zhu L, Xie P, et al. Effects of acupuncture on breast cancer-related lymphoedema: A systematic review and meta-analysis.16(2):97-102. <https://www.sciencedirect.com/science/article/pii/S1550830719300771?via%3Dihub>.

461. Yuan J, Purepong N, Kerr DP, Park J, Bradbury I, McDonough S. Effectiveness of acupuncture for low back pain: a systematic review.33(23):E887-900.

462. Yuan QL, Wang P, Liu L, et al. Acupuncture for musculoskeletal pain: A meta-analysis and meta-regression of sham-controlled randomized clinical trials.6:30675. <https://www.ncbi.nlm.nih.gov/pmc/articles/PMC4965798/pdf/srep30675.pdf>.

463. Yuan XX, Wang BY, Yang L, Li DD, Liang H, Zhang YL. Efficacy of manual acupuncture on functional dyspepsia: A meta-analysis of randomized, controlled trials. 2017;14(6):38-55. <https://www.embase.com/search/results?subaction=viewrecord&id=L619252422&from=export> <http://dx.doi.org/10.21010/ajtcam.v14i6.5>.

464. Yue J, Liu M, Li J, et al. Acupuncture for the treatment of hiccups following stroke: a systematic review and meta-analysis.35(1):2-8.

465. Yun JM, Lee SH, Cho JH, Kim KW, Ha IH. The effects of acupuncture on occipital neuralgia: a systematic review and meta-analysis.20(1):171. <https://www.ncbi.nlm.nih.gov/pmc/articles/PMC7268636/pdf/12906_2020_Article_2955.pdf>.

466. Zeng YC, Luo TZ, Finnegan-John J, Cheng ASK. Meta-Analysis of Randomized Controlled Trials of Acupuncture for Cancer-Related Fatigue.13(3):193-200.

467. Zhan J, Pan RH, Zhou MC, et al. Electroacupuncture as an adjunctive therapy for motor dysfunction in acute stroke survivors: a systematic review and meta-analyses.8(1).

468. Zhan J, Xiong B, Zhang P, et al. Abdominal Acupuncture as an Adjunctive Therapy for the Recovery of Motor Function After Stroke: A Systematic Review and Meta-Analysis of Randomized Controlled Trials. 2021;12:705771. <https://www.ncbi.nlm.nih.gov/pmc/articles/PMC8505526/pdf/fneur-12-705771.pdf>.

469. Zhang BL, Han Y, Huang X, et al. Acupuncture is effective in improving functional communication in post-stroke aphasia: Asystematic review and meta-analysis of randomized controlled trials.131(9):221-232.

470. Zhang GC, Fu WB, Xu NG, et al. Meta analysis of the curative effect of acupuncture on post-stroke depression.32(1):6-11.

471. Zhang J, Cao L, Wang Y, Jin Y, Xiao X, Zhang Q. Acupuncture for Premenstrual Syndrome at Different Intervention Time: A Systemic Review and Meta-Analysis. 2019;2019:6246285. <https://www.ncbi.nlm.nih.gov/pmc/articles/PMC6614973/pdf/ECAM2019-6246285.pdf>.

472. Zhang J, Chen J, Chen J, et al. Early filiform needle acupuncture for poststroke depression: a meta-analysis of 17 randomized controlled clinical trials.9(7):773-784. <https://www.ncbi.nlm.nih.gov/pmc/articles/PMC4146280/pdf/NRR-9-773.pdf>.

473. Zhang JH, Li XM, Xu J, Ernst E. Laser Acupuncture for the Treatment of Asthma in Children: A Systematic Review of Randomized Controlled Trials.49(7):773-777. <https://www.tandfonline.com/doi/full/10.3109/02770903.2012.691194>.

474. Zhang K, Gao C, Li C, et al. Acupuncture for Acute Pancreatitis: A Systematic Review and Meta-analysis.48(9):1136-1147.

475. Zhang K, Zhou S, Wang C, Xu H, Zhang L. Acupuncture on Obesity: Clinical Evidence and Possible Neuroendocrine Mechanisms. 2018;2018:6409389. <https://www.ncbi.nlm.nih.gov/pmc/articles/PMC6022277/pdf/ECAM2018-6409389.pdf>.

476. Zhang N, Hou L, Yan P, et al. Electro-acupuncture vs. sham electro-acupuncture for chronic severe functional constipation: A systematic review and meta-analysis.54:102521. <https://www.sciencedirect.com/science/article/pii/S096522992030042X?via%3Dihub>.

477. Zhang N, Houle T, Hindiyeh N, Aurora SK. Systematic Review: Acupuncture vs Standard Pharmacological Therapy for Migraine Prevention.60(2):309-317. <https://headachejournal.onlinelibrary.wiley.com/doi/10.1111/head.13723>.

478. Zhang Q, Gong J, Dong HX, Xu SB, Wang W, Huang GY. Acupuncture for chronic fatigue syndrome: a systematic review and meta-analysis.37(4):211-222.

479. Zhang RC, Wu T, Wang RH, Wang D, Liu Q. Compare the efficacy of acupuncture with drugs in the treatment of Bell's palsy A systematic review and meta-analysis of RCTs.98(19).

480. Zhang T, Chon TY, Liu B, et al. Efficacy of acupuncture for chronic constipation: a systematic review. 2013;41(4):717-742. <https://www.worldscientific.com/doi/10.1142/S0192415X13500493?url_ver=Z39.88-2003&rfr_id=ori:rid:crossref.org&rfr_dat=cr_pub%3dpubmed>.

481. Zhang W, Ma L, Bauer BA, Liu Z, Lu Y. Acupuncture for benign prostatic hyperplasia: A systematic review and meta-analysis. 2017;12(4):e0174586. <https://www.ncbi.nlm.nih.gov/pmc/articles/PMC5380320/pdf/pone.0174586.pdf>.

482. Zhang X, Ding WT, Wang Z, Gu XL, Zhu WZ. The effectiveness and safety of acupuncture for the treatment of myasthenia gravis: a systematic review and meta-analysis of randomized controlled trials.8(5):576-585. <https://apm.amegroups.com/article/viewFile/32183/26660>.

483. Zhang X, Lee MS, Smith CA, et al. Effects of acupuncture during in vitro fertilization or intracytoplasmic sperm injection: An updated systematic review and meta-analysis.23:14-25.

484. Zhang X, Wang X, Zhang B, Yang S, Liu D. Effects of acupuncture on breast cancer-related lymphoedema: a systematic review and meta-analysis of randomised controlled trials.37(1):16-24.

485. Zhang XC, Chen H, Xu WT, Song YY, Gu YH, Ni GX. Acupuncture therapy for fibromyalgia: a systematic review and meta-analysis of randomized controlled trials. 2019;12:527-542. <https://www.dovepress.com/getfile.php?fileID=47779>.

486. Zhang XC, Xu XP, Xu WT, et al. Acupuncture therapy for sudden sensorineural hearing loss: a systematic review and meta-analysis of randomized controlled trials. 2015;10(4):e0125240. <https://www.ncbi.nlm.nih.gov/pmc/articles/PMC4412536/pdf/pone.0125240.pdf>.

487. Zhang XW, Wang FM, Yu SS, Zhou QH. The effect of acupuncture on Bell's palsy: an overall and cumulative meta-analysis of randomized controlled trials. 2018;11(4):3309-+.

488. Zhang XY, Li YX, Liu DL, Zhang BY, Chen DM. The effectiveness of acupuncture therapy in patients with post-stroke depression: An updated meta-analysis of randomized controlled trials.98(22):e15894. <https://www.ncbi.nlm.nih.gov/pmc/articles/PMC6708961/pdf/medi-98-e15894.pdf>.

489. Zhang Y, Lin L, Li HL, Hu Y, Tian L. Effects of acupuncture on cancer-related fatigue: a meta-analysis.26(2):415-425. <https://link.springer.com/content/pdf/10.1007/s00520-017-3955-6.pdf>.

490. Zhang Y, Sun Y, Li D, et al. Acupuncture for Breast Cancer: A Systematic Review and Meta-Analysis of Patient-Reported Outcomes. 2021;11:646315. <https://www.ncbi.nlm.nih.gov/pmc/articles/PMC8222976/pdf/fonc-11-646315.pdf>.

491. Zhang Y, Wang ZJ, Jiang XD, Lv ZM, Wang L, Lu LM. Effectiveness of Acupuncture for Poststroke Aphasia: A Systematic Review and Meta-Analysis of Randomized Controlled Trials %J COMPLEMENTARY MEDICINE RESEARCH.

492. Zhang Z, Chen M, Zhang L, et al. Meta-analysis of acupuncture therapy for the treatment of stable angina pectoris. 2015;8(4):5112-5120. <https://www.ncbi.nlm.nih.gov/pmc/articles/PMC4483941/pdf/ijcem0008-5112.pdf>.

493. Zhao FY, Fu QQ, Kennedy GA, et al. Comparative Utility of Acupuncture and Western Medication in the Management of Perimenopausal Insomnia: A Systematic Review and Meta-Analysis. 2021;2021:5566742. <https://www.ncbi.nlm.nih.gov/pmc/articles/PMC8093060/pdf/ECAM2021-5566742.pdf>.

494. Zhao FY, Fu QQ, Kennedy GA, et al. Can acupuncture improve objective sleep indices in patients with primary insomnia? A systematic review and meta-analysis.80:244-259. <https://www.sciencedirect.com/science/article/pii/S1389945721000708?via%3Dihub>.

495. Zhao L, Guo Y, Wang W, Yan LJ. Systematic review on randomized controlled clinical trials of acupuncture therapy for neurovascular headache.17(8):580-586. <https://link.springer.com/content/pdf/10.1007/s11655-011-0709-z.pdf>.

496. Zhao QY, Yan CC, Dan M, Jia HL. Efficacy and safety of acupuncture for urinary retention after hysterectomy A systematic review and meta-analysis.100(22).

497. Zhao XF, Hu HT, Li JS, et al. Is Acupuncture Effective for Hypertension? A Systematic Review and Meta-Analysis. 2015;10(7):e0127019. <https://www.ncbi.nlm.nih.gov/pmc/articles/PMC4514875/pdf/pone.0127019.pdf>.

498. Zhao YW, Zhou J, Mo Q, Wang Y, Yu JN, Liu ZS. Acupuncture for adults with overactive bladder A systematic review and meta-analysis of randomized controlled trials.97(8).

499. Zheng CH, Huang GY, Zhang MM, Wang W. Effects of acupuncture on pregnancy rates in women undergoing in vitro fertilization: a systematic review and meta-analysis.97(3):599-611.

500. Zheng GQ, Zhao ZM, Wang Y, et al. Meta-analysis of scalp acupuncture for acute hypertensive intracerebral hemorrhage.17(4):293-299. <https://www.liebertpub.com/doi/pdf/10.1089/acm.2010.0156?download=true>.

501. Zheng H, Chen R, Zhao X, et al. Comparison between the Effects of Acupuncture Relative to Other Controls on Irritable Bowel Syndrome: A Meta-Analysis. 2019;2019. <https://www.embase.com/search/results?subaction=viewrecord&id=L2004025217&from=export> <http://dx.doi.org/10.1155/2019/2871505>.

502. Zheng RQ, Qing P, Han M, et al. The Effect of Acupuncture on Glucose Metabolism and Lipid Profiles in Patients with PCOS: A Systematic Review and Meta-Analysis of Randomized Controlled Trials.2021.

503. Zheng XZ, Xiong QJ, Liu D, Wei K, Lai Y. Effectiveness of Acupuncture Therapy on Postoperative Nausea and Vomiting After Gynecologic Surgery: A Meta-Analysis and Systematic Review.36(5):564-572. <https://www.sciencedirect.com/science/article/pii/S1089947220303828?via%3Dihub>.

504. Zhi FY, Liu J, Ma XP, et al. Manual Acupuncture for Optic Atrophy: A Systematic Review and Meta-Analysis. 2019;2019:1735967. <https://www.ncbi.nlm.nih.gov/pmc/articles/PMC6332962/pdf/ECAM2019-1735967.pdf>.

505. Zhong L, Wang J, Li F, Bao X, Liu H, Wang P. The Effectiveness of Acupuncture for Dysphagia after Stroke: A Systematic Review and Meta-Analysis. 2021;2021:8837625. <https://www.ncbi.nlm.nih.gov/pmc/articles/PMC7837771/pdf/ECAM2021-8837625.pdf>.

506. Zhong Y, Zeng F, Li J, Yang Y, Zhong S, Song Y. Electroacupuncture for Postoperative Urinary Retention: A Systematic Review and Meta-Analysis. 2018;2018:7612618. <https://www.ncbi.nlm.nih.gov/pmc/articles/PMC6083550/pdf/ECAM2018-7612618.pdf>.

507. Zhong Y, Zeng F, Liu W, Ma J, Guan Y, Song Y. Acupuncture in improving endometrial receptivity: a systematic review and meta-analysis.19(1):61. <https://www.ncbi.nlm.nih.gov/pmc/articles/PMC6417024/pdf/12906_2019_Article_2472.pdf>.

508. Zhong YJ, Song Y, Zeng FZ, Zhao Y, Beth L, Guan YG. Effectiveness of electroacupuncture for female stress urinary incontinence: a systematic review and Meta-analysis.40(5):707-720.

509. Zhong YM, Luo XC, Chen Y, et al. Acupuncture versus sham acupuncture for simple obesity: a systematic review and meta-analysis.96(1134):221-227. <https://www.ncbi.nlm.nih.gov/pmc/articles/PMC7146934/pdf/postgradmedj-2019-137221.pdf>.

510. Zhou J, Peng W, Xu M, Li W, Liu Z. The effectiveness and safety of acupuncture for patients with Alzheimer disease: a systematic review and meta-analysis of randomized controlled trials.94(22):e933. <https://www.ncbi.nlm.nih.gov/pmc/articles/PMC4616366/pdf/medi-94-e933.pdf>.

511. Zhou L, Wang Y, Qiao J, Wang QM, Luo X. Acupuncture for Improving Cognitive Impairment After Stroke: A Meta-Analysis of Randomized Controlled Trials. 2020;11:549265. <https://www.ncbi.nlm.nih.gov/pmc/articles/PMC7793937/pdf/fpsyg-11-549265.pdf>.

512. Zhou M, He L, Zhou D, et al. Acupuncture for bell's palsy. 2009;15(7):759-764. <https://www.embase.com/search/results?subaction=viewrecord&id=L355004052&from=export> <http://dx.doi.org/10.1089/acm.2008.0179>

https://www.liebertpub.com/doi/pdf/10.1089/acm.2008.0179?download=true.

513. Zhou WM, Su JW, Zhang HJ. Efficacy and Safety of Acupuncture for the Treatment of Functional Dyspepsia: Meta-Analysis.22(5):380-389. https://www.liebertpub.com/doi/pdf/10.1089/acm.2014.0400?download=true.

514. Zhu F, Yin S, Zhu X, et al. Acupuncture for Relieving Abdominal Pain and Distension in Acute Pancreatitis: A Systematic Review and Meta-Analysis. 2021;12:786401. <https://www.ncbi.nlm.nih.gov/pmc/articles/PMC8678533/pdf/fpsyt-12-786401.pdf>.

515. Zhu J, Guo Y, Liu S, et al. Acupuncture for the treatment of gastro-oesophageal reflux disease: a systematic review and meta-analysis.35(5):316-323.

516. Zhu X, Hamilton KD, McNicol ED. Acupuncture for pain in endometriosis. (9):Cd007864.

517. Zhuang L, Yang Z, Zeng X, et al. The preventive and therapeutic effect of acupuncture for radiation-induced xerostomia in patients with head and neck cancer: a systematic review.12(3):197-205.

518. Zheng J, Lai X, Zhu W, Huang Y, Chen C, Chen J. Effects of Acupuncture Combined with Rehabilitation on Chronic Pelvic Pain Syndrome in Females: A Meta-Analysis Running Head-Acupuncture Combined with Rehabilitation on Chronic Pelvic Pain. *J Healthc Eng.* 2022;2022:8770510.<http://doi.org/10.1155/2022/8770510>

519. Deng K-f, Li L-h, Pan T-z, et al. Meta-analysis and trial sequential analysis on blood uric acid and joint function in gouty arthritis treated with fire needling therapy in comparison with western medication☆. *World Journal of Acupuncture - Moxibustion.* 2022;32(1):49-60.<http://doi.org/https://doi.org/10.1016/j.wjam.2021.08.010>

520. Hou Y, Liu Y, Li M, Ning B, Wen Z, Fu W. Acupuncture plus Rehabilitation for Unilateral Neglect after Stroke: A Systematic Review and Meta-Analysis. *Evid Based Complement Alternat Med.* 2020;2020:5301568.<http://doi.org/10.1155/2020/5301568>

521. Li LX, Deng K. Acupuncture combined with swallowing training for poststroke dysphagia: a meta-analysis of randomised controlled trials. *Acupunct Med.* 2019;37(2):81-90.<http://doi.org/10.1136/acupmed-2016-011305>

522. Huang T, Shu X, Huang YS, Cheuk DK. Complementary and miscellaneous interventions for nocturnal enuresis in children. *Cochrane Database Syst Rev.* 2011(12):Cd005230.<http://doi.org/10.1002/14651858.CD005230.pub2>

523. Lee SW, Nam MH, Lee BC. Herbal acupuncture for type 2 diabetes: A meta-analysis. *Exp Ther Med.* 2017;13(6):3249-3256.<http://doi.org/10.3892/etm.2017.4379>

524. Chan YY, Lo WY, Yang SN, Chen YH, Lin JG. The benefit of combined acupuncture and antidepressant medication for depression: A systematic review and meta-analysis. *J Affect Disord.* 2015;176:106-117.<http://doi.org/10.1016/j.jad.2015.01.048>

525. Jiang HL, Jia P, Fan YH, et al. Manual Acupuncture or Combination with Vitamin B to Treat Diabetic Peripheral Neuropathy: A Systematic Review and Meta-Analysis of Randomized Controlled Trials. *Biomed Res Int.* 2020;2020:4809125.<http://doi.org/10.1155/2020/4809125>

526. Liu TT, Shi J, Epstein DH, Bao YP, Lu L. A meta-analysis of acupuncture combined with opioid receptor agonists for treatment of opiate-withdrawal symptoms. *Cell Mol Neurobiol.* 2009;29(4):449-454.<http://doi.org/10.1007/s10571-008-9336-4>

527. Li Y, Barajas-Martinez H, Li B, et al. Comparative Effectiveness of Acupuncture and Antiarrhythmic Drugs for the Prevention of Cardiac Arrhythmias: A Systematic Review and Meta-analysis of Randomized Controlled Trials. *Front Physiol.* 2017;8:358.<http://doi.org/10.3389/fphys.2017.00358>

528. Yang L, Di YM, Shergis JL, et al. A systematic review of acupuncture and Chinese herbal medicine for postpartum depression. *Complement Ther Clin Pract.* 2018;33:85-92.<http://doi.org/10.1016/j.ctcp.2018.08.006>

529. Xie Q, Chen X, Xiao J, et al. Acupuncture combined with speech rehabilitation training for post-stroke dysarthria: A systematic review and meta-analysis of randomized controlled trials. *Integr Med Res.* 2020;9(4):100431.<http://doi.org/10.1016/j.imr.2020.100431>

530. Wong V, Cheuk DK, Lee S, Chu V. Acupuncture for acute management and rehabilitation of traumatic brain injury. *Cochrane Database Syst Rev.* 2013(3):Cd007700.<http://doi.org/10.1002/14651858.CD007700.pub3>

531. Wang X, Xiong J, Yang J, et al. Meta-analysis of the clinical effectiveness of combined acupuncture and Western Medicine to treat post-stroke depression. *J Tradit Chin Med.* 2021;41(1):6-16.<http://doi.org/10.19852/j.cnki.jtcm.2021.01.002>

532. Wen X, Li K, Wen H, et al. Acupuncture-Related Therapies for Parkinson's Disease: A Meta-Analysis and Qualitative Review. *Front Aging Neurosci.* 2021;13:676827.<http://doi.org/10.3389/fnagi.2021.676827>

533. Zhang K, Cui G, Gao Y, Shen W. Does acupuncture combined with antidepressants have a better therapeutic effect on post-stroke depression? A systematic review and meta-analysis. *Acupunct Med.* 2021;39(5):432-440.<http://doi.org/10.1177/0964528420967675>

534. Yang J, Li X, Li C, et al. Comparative efficacy and safety of acupuncture and Western medicine for poststroke thalamic pain. *Anat Rec (Hoboken).* 2022.<http://doi.org/10.1002/ar.24902>

# Table S1: Basic characteristics of the included studies

| Author, year | Country | No of studies | Participants | Intervention | Control | Adverse event |
| --- | --- | --- | --- | --- | --- | --- |
| Abdi F, 2021 | Iran | 13 | men and women with sexual dysfunction | electroacupuncture warm needling therapy manual acupuncture | no control other therapy | tiredness bleeding or bruising discomfort pain |
| Amorim D, 2018 | Portugal | 13 | patients with anxiety | acupuncture electroacupuncture | western medicine sham acupuncture no intervention | NA |
| Ang L, 2020 | Korea | 6 | patients with diabetic retinopathy | acupuncture + oral calcium dobesilate | oral calcium dobesilate | NR |
| Ang L, 2021 | Korea | 12 | children with nocturnal crying | acupuncture | No control | NR |
| Ba J, 2013 | China | 11 | patients with dry eye | acupuncture | artificial tears | pain hematoma |
| Bae K, 2015 | Australia | 4 | patients with arthralgia | electroacupuncture manual acupuncture | sham acupuncture sham electroacupuncture waitlist control usual care | bleeding or bruising pain |
| Yang B, 2014 | China | 9 | patients with fibromyalgia syndrome | acupuncture electroacupuncture | sham acupuncture western medicine rehabilitation training | mild bruising and sorenes mild vasovagal symptoms |
| Bai ZH, 2015 | China | 16 | patients with stroke | eye acupuncture | usual care | NR |
| Park SW, 2014 | Korea | 8 | patients with stroke | electroacupuncture manual acupuncture | sham acupuncture no acupuncture | NR |
| Baviera AF,2019 | Brazil | 5 | adults with cancer | acupuncture (without electrical, laser or auricular stimulation) | other therapy | NR |
| Befus D, 2018 | USA | 3 | perimenopausal and postmenopausal women with bothersome vasomotor symptoms | acupuncture | sham acupuncture | bleeding or bruising pain dizziness aggravation of symptoms minor swelling pruritus skin allergy reaction heat or sweating psychological disorder motor disorders |
| Ben-Arie E, 2020 | China | 13 | patients with primary adhesive capsulitis patients | electroacupuncture manual acupuncture | western medicine sham acupuncture rehabilitation training | NR |
| Blanco-Díaz M, 2022 | Spain | 9 | adults with unilateral shoulder pain of non-traumatic origin | dry needling | sham acupuncture usual care no intervention | pain  NA |
| Bonomo P, 2022 | Italy | 5 | patients with radiation ‑induced toxicity in head and neck squamous cell carcinoma | acupuncture | sham acupuncture usual care | discomfort bleeding or bruising |
| Bower WF, 2005 | Hong Kong, China | 11 | children (<18 years) with nocturnal enuresis | acupuncture | other therapy | NR |
| Cai YY, 2017 | China | 22 | patients with stroke | electroacupuncture+ rehabilitation training | rehabilitation training | NR |
| Cao HJ, 2013 | China | 12 | participants (average age 61 years old) with vascular mild cognitive impairment | body acupuncture scalp acupuncture | western medicine rehabilitation training | NR |
| Cao JP, 2018 | China | 23 | patients with primary peripheral facial paralysis | Warm needling therapy | other therapy | NR |
| Cao L, 2012 | China | 14 | patients with knee osteoarthritis | acupuncture | sham acupuncture | NR |
| Casimiro L, 2010 | Canada | 2 | adult patients with rheumatoid arthritis | acupuncture electroacupuncture | sham acupuncture other therapy | NR |
| Chai QYC, 2015 | China | 8 | women with melasma | manual acupuncture manual acupuncture + other therapy | sham acupuncture other therapy  no treatment | NR |
| Chan YT, 2021 | China | 16 | participants with breast cancer | acupuncture | sham acupuncture no treatment waitlist control | bleeding or bruising discomfort subcutaneous nodulations dizziness pruritus  psychological disorder pain |
| Chang H, 2020 | Korea | 7 | patients with humeral fractures | manual acupuncture | rehabilitation training | pain |
| Chang SC, 2016 | Taiwan, China | 7 | participants with chronic prostatitis/chronic pelvic pain syndrome | acupuncture | sham acupuncture western medicine | hematoma pain NA |
| Chao GQ, 2014 | China | 6 | patients with irritable bowel syndrome | acupuncture | usual care sham acupuncture western medicine | NR |
| Chau JPC, 2018 | Hong Kong, China | 29 | adults with stroke | acupuncture | rehabilitation training | NR |
| Chen C, 2019 | China | 21 | patients with type 2 diabetes mellitus | acupuncture acupuncture + usual care | sham acupuncture usual care no treatment | NA |
| Chen H, 2020 | China | 7 | patients with urinary incontinence | acupuncture | western medicine | NR |
| Chen H, 2022 | China | 4 | patients with post-hemorrhoidectomy pain | acupuncture | sham acupuncture western medicine | dizziness urination disorder digestive system symptoms NA |
| Chen HY, 2007 | Hong Kong, China | 6 | patients with Insomnia | auricular acupuncture | sham auricular acupuncture  western medicine no treatment | NR |
| Chen JR, 2018 | China | 33 | patients with overweight and obesity | manual acupuncture electroacupuncture laser acupuncture | sham acupuncture usual care | erythema discomfort bleeding or bruising pain inflammation dizziness NA |
| Chen JF, 2021 | China | 17 | patients diagnosed as myelosuppression after chemotherapy | acupoint injection | western medicine | swelling pain |
| Chen J, 2012 | China | 21 | participants with angina pectoris | acupuncture acupuncture + western medicine | western medicine | NR |
| Chen LC, 2017 | Taiwan, China | 5 | patients with breast cancer | acupuncture | sham acupuncture | pain numbness |
| Chen N, 2017 | China | 11 | patients with knee osteoarthritis | electroacupuncture | pharmacological treatment | dizziness |
| Chen N, 2010 | China | 6 | participants with Bell's palsy | acupuncture acupuncture + western medication | sham acupuncture other therapy | NR |
| Chen P, 2021 | China | 8 | patients with nonalcoholic fatty liver disease | electroacupuncture warm acupuncture manual acupuncture acupoint thread embedding | western medicine | pain NA |
| Chen SW, 2018 | China | 20 | patients diagnosed with sudden sensorineural hearing loss | electroacupuncture manual acupuncture acupuncture + western medicine | western medicine | NA |
| Chen WJ, 2021 | China | 10 | patients with total knee arthroplasty | electroacupuncture | other therapy  sham acupuncture | NR |
| Chen W, 2013 | China | 25 | patient with diabetic peripheral neuropathy | manual acupuncture | sham acupuncture no intervention | NR |
| Chen XM, 2022 | China | 8 | patients with Alzheimer's disease | warm acupuncture | pharmacotherapy | NR |
| Chen XT, 2022 | China | 15 | patients with acute gastrointestinal injury | acupuncture + western medicine | western medicine | NR |
| Chen YC, 2021 | Taiwan, China | 10 | participants with attention deficit hyperactivity disorder | acupuncture acupuncture + western medicine | western medicine waitlist control | NS NA |
| Chen YP, 2016 | China | 12 | hot flashes in women with breast cancer | electroacupuncture ear acupuncture acupoint injection | sham acupuncture western medicine other therapy | bleeding or bruising tiredness pruritus nausea |
| Chen YP, 2021 | China | 6 | participants with post-stoke fatigue | acupuncture + rehabilitation training | rehabilitation training | NA |
| Chen Z, 2021 | China | 9 | patients with total knee arthroplasty | acupuncture warm acupuncture | sham acupuncture other therapy | pain |
| ChenZH, 2018 | China | 9 | adult patients with opioid use disorder | acupuncture | sham acupuncture other therapy no intervention | NR |
| Cheng K, 2017 | China | 6 | participants with acute hordeola | acupuncture | other therapy | hematoma  infection |
| Cheong KB, 2013 | China | 30 | patients underwent surgery | electroacupuncture | sham acupuncture western medicine usual care | erythema |
| Cheuk DKL, 2014 | China | 17 | people with an epilepsy syndrome | laser acupuncture electroacupuncture acupoint thread embedding | western medicine sham acupuncture no intervention | dizziness tiredness nausea anorexia |
| Cheuk DKL, 2011 | China | 10 | people with autism spectrum disorders | acupuncture | western medicine sham acupuncture no intervention | pain psychological disorder |
| Cheuk DKL, 2009 | China | 7 | people with insomnia | acupuncture | sham acupuncture | pain |
| Chien TJ, 2017 | Taiwan, China | 13 | women with breast cancer | acupuncture | sham acupuncture no treatment | NR |
| Chien TJ, 2015 | Taiwan, China | 5 | participants were postmenopausal women with breast cancer | acupuncture | sham acupuncture | NR |
| Chien TJ, 2019 | Taiwan, China | 6 | participants with breast cancer–related lymphoedema | acupuncture | other therapy  western medicine waitlist control | NR |
| Chien TJ, 2019 | Taiwan, China | 13 | participants with breast cancer-related menopause symptoms | acupuncture electroacupuncture | sham acupuncture western medicine usual care | NR |
| Chiu HY, 2016 | Taiwan, China | 31 | sleep disturbances in perimenopausal and postmenopausal women | manual acupuncture electroacupuncture laser acupuncture auricular acupuncture | sham acupuncture Chinese medicine western medicine | pruritus nausea sensitivity to cold pain pruritus neuromuscular disease bleeding or bruising NA |
| Chiu H, 2016 | Taiwan, China | 29 | adult cancer patients (aged ≥18 years) | acupuncture | waiting list no treatment usual care | digestive system symptoms dizziness heat or sweating numbness pain bleeding or bruising |
| Chiu HY, 2014 | Taiwan, China | 12 | women (40-60 years) with natural menopause | acupuncture electroacupuncture laser acupuncture ear acupuncture | sham acupuncture western medicine usual care | NA |
| ChoKH, 2017 | Korea | 3 | patients with idiopathic Parkinson’s disease | acupoint injection | no intervention western medicine | pruritus |
| Cho SH, 2010 | Korea | 27 | women of reproductive age with primary dysmenorrhoea | electroacupuncture laser acupuncture auricular acupuncture  acupoint injection | waitlist control no treatment usual care western medicine psychological interventions | NS |
| Cho SH, 2010 | Korea | 9 | women (any age) with premenstrual syndrome or premenstrual dysphoric disorder | acupuncture electroacupuncture laser acupuncture acupoint injection | sham acupuncture western medicine other therapy  no treatment | hematoma |
| Cho SH, 2010 | Korea | 10 | women with pain relief in labour | acupuncture electroacupuncture auricular acupuncture | sham acupuncture usual care other therapy | NA |
| Cho SH, 2008 | Korea | 31 | participant with overweight/obese | acupuncture auricular acupuncture electroacupuncture laser acupuncture | sham acupuncture usual care waitlist control western medicine physical activity interventions | NA skin redness pain discomfort bleeding or bruising digestive system symptoms dry mouth headache tiredness blood pressure disorder palpitations dizziness |
| Cho SH, 2009 | Korea | 11 | women with vasomotor menopausal symptoms | acupuncture acupoint catgut | sham acupuncture western medicine | bleeding or bruising discomfort insomnia pain pruritus neuromuscular disease minor swelling NA |
| Cho SH, 2009 | Korea | 11 | all patients with alcohol dependency | acupuncture | sham acupuncture western medicine usual care | tiredness bleeding or bruising pain |
| Cho WC, 2018 | China | 5 | patients with all types of overweight or obesity | acupoint thread embedding | sham acupoint thread embedding | psychological disorder neuromuscular disease bleeding or bruising induration erythema |
| Cho YH, 2014 | Korea | 5 | patients with back surgery | acupuncture | sham acupuncture | NA |
| Choi GH, 2019 | Korea | 12 | adults (≥18years) with carpel tunnel syndrome | acupuncture | sham acupuncture western medicine | NS |
| Choi TY, 2016 | Korea | 6 | participants with a sleep disorder | acupuncture | sham acupuncture western medicine | tiredness pruritus nausea pain bleeding or bruising NA |
| Choi TY, 2012 | Korea | 5 | patients with acute, persistent or intractable hiccups resulting from cancer | acupuncture | western medicine | NR |
| Choi TY, 2012 | Korea 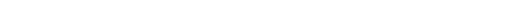 | 15 | participants with liver cancer, stomach cancer, late-stage cancer | acupuncture | sham acupuncture western medicine | NA |
| Chung SY, 2016 | Korea | 19 | patients with tourette syndrome | acupuncture | western medicine | NA |
| Clark RJ, 2015 | UK | 8 | patients with plantar heel pain | acupuncture | usual care | NS |
| Cooper K, 2016 | UK | 10 | patients with premature ejaculation | acupuncture | sham acupuncture | NA |
| Hou XB, 2020 | China | 25 | patients with cancer pain | acupuncture | western medicine | NS |
| Coyle MH, 2015 | Australia | 6 | patients (27-49 year) with spsoriasis vulgaris | acupuncture | sham acupuncture western medicine | NA |
| Coyle MH, 2020 | Australia | 8 | women with vitro fertilisation | acupuncture | sham acupuncture | NS |
| Cui W, 2018 | China | 12 | adults with asthma | acupoint thread embedding acupoint thread embedding + western medicine | western medicine | dizziness skin redness minor swelling NA |
| Cui XM, 2016 | China | 3 | participants (≥ 18 years) with erectile dysfunction | electroacupuncture manual acupuncture | sham acupuncture western medicine | NA |
| Cui Y, 2022 | China | 8 | women (≥ 18 years) with mixed urinary incontinence | electroacupuncture | rehabilitation training | hematoma digestive system symptoms |
| Dai L, 2021 | China | 59 | participants with pain in palliative cancer management | electroacupuncture manual acupuncture laser acupuncture acupuncture + western medicine | sham acupuncture western medicine | tiredness pain |
| Dai L, 2019 | China | 15 | adult patients with nonalcoholic fatty liver disease | acupoint thread embedding | western medicine | pain erythema subcutaneous nodulations fever digestive system symptoms |
| Davis MA, 2008 | Boston | 5 | patients with tension-type headache | acupuncture | sham acupuncture | headache haematoma dizziness |
| Deare JC, 2013 | Australia | 9 | patients with fibromyalgia | acupuncture | sham acupuncture other therapy  western medicine | NS |
| Deng M, 2016 | China | 5 | patients with amnestic mild cognitive impairment | electroacupuncture electroacupuncture + western medicine | western medicine | bleeding or bruising dizziness |
| Dimitrova A, 2017 | Portland | 15 | adults (>18 years) with peripheral neuropathy | acupuncture manual acupuncture electroacupuncture | sham acupuncture western medicine | bleeding or bruising discomfort pain discomfort |
| Dodin S, 2013 | Canada | 8 | women with menopausal hot flushes | acupuncture | sham acupuncture waiting list no intervention | NA bleeding or bruising |
| Dong B, 2017 | China | 18 | adult (>18 years) with depression-related insomnia | acupuncture | sham acupuncture western medicine | hematomas pain |
| Dong Be, 2021 | China | 13 | adult patients (≥18 years) with cancer and suffering from pain | wrist-ankle acupuncture wrist-ankle acupuncture +western medicine | western medicine | bleeding or bruising dizziness |
| Ee CC, 2007 | Australia | 2 | patients with pelvic and back pain in pregnancy | acupuncture | sham acupuncture other therapy  usual care no intervention | pain bleeding or bruising heat or sweating nausea tiredness haematoma |
| Ernst E, 1999 | England | 6 | patients with temporomandibular joint dysfunction | acupuncture | no treatment western medicine | neuromuscular disease |
| Ezzo J, 2001 | USA | 7 | patients with osteoarthritis of the knee | manual acupuncture | waitlist control sham acupuncture rehabilitation training | NS |
| Ezzo J, 2014 | USA | 11 | cancer patients receiving chemotherapy | acupuncture electroacupuncture manual acupuncture | western medicine | erythema pain NA |
| Fan WJ, 2020 | China | 38 | patients with stroke | electroacupuncture manual acupuncture acupuncture + rehabilitation training | rehabilitation training | dizziness needle sticking NA |
| Fang SJ, 2017 | China | 23 | patients with overweight or obesity | acupuncture electroacupuncture acupoint thread embedding | sham acupuncture rehabilitation training | blisters erythema bleeding or bruising induration digestive system symptoms NA |
| Farag AM, 2020 | United States | 4 | patients with persistent regional myofascial head and neck pain | acupuncture dry needling therapy | sham acupuncture no intervention | NA |
| Feng SY, 2015 | China | 13 | patients with allergic rhinitis | electroacupuncture manual acupuncture | sham acupuncture western medicine other therapy | pain erythema pruritus bleeding or bruising dizziness numbness headache tiredness NA |
| Fernandez-de-las-Penas C, 2021 | Spain | 7 | adults (＞18 years) with stroke | dry needling therapy dry needling therapy +rehabilitation training | rehabilitation training sham dry needling therapy | myasthenia or myalgia pain local cutaneous vasodilatation palpitation or sweating blood pressure disorder psychological disorder |
| Fernández-Jané C, 2020 | Spain | 33 | patients with chronic obstructive pulmonary disease | acupuncture ear acupuncture | sham acupuncture western medicine | NA |
| Fern, 2019 | Spain | 28 | patients with chronic obstructive pulmonary disease | filiform needle acupuncture | sham acupuncture western medicine other therapy | pain hematoma NA |
| France S, 2014 | Australia | 3 | patients with cervicogenic or tension-type headache | dry needling therapy | western medicine no intervention | NR |
| Fu LM, 2009 | USA | 14 | patients with neck pain | acupuncture | sham acupuncture waitlist control usual care | NA |
| Furlan AD, 2005 | Canada | 35 | adults (＞18 years) with nonspecific low back pain and myofascial pain syndrome in the low back region | acupuncture dry needling therapy | sham acupuncture no treatment | NA |
| Gao X, 2021 | China | 18 | patients with gastrointestinal function recovery after gynecological surgery | electroacupuncture + usual care/western medicine | usual care western medicine | pain bleeding or bruising |
| Garcia MK, 2016 | USA | 8 | women with breast cancer | acupuncture electroacupuncture | western medicine no treatment | NR |
| Gates S, 2006 | UK | 7 | people with cocaine or crack cocaine dependence | auricular acupuncture | sham acupuncture other therapy | NR |
| Ge SQ, 2020 | China | 30 | participants with illicit drug use disorder and suffering from drug withdrawal syndrome | electroacupuncture manual acupuncture auricular acupuncture | no treatment sham acupuncture western medicine | bleeding or bruising pain myasthenia or myalgia |
| Giovanardi GM, 2020 | Italy | 9 | adult (≥18 years) with migraine | acupuncture | western medicine | NS |
| Grant S, 2017 | USA | 7 | adults with posttraumatic stress disorder | acupuncture | usual care | pain bleeding or bruising hematoma |
| Grant S, 2016 | USA | 41 | participants with alcohol, opioid, stimulant, and/or cannabis use disorder | auricular acupuncture | usual care | convulsions psychological disorder bleeding or bruising |
| Green S, 2013 | Australia | 4 | adults (>16 years) with lateral elbow pain | acupuncture | sham acupuncture other therapy | NR |
| Sally Green, 2008 | Australia | 9 | adults (>16 years) with shoulder pain | acupuncture | other therapy | NS |
| Guo TP, 2015 | China | 43 | children with obesity and abdominal obesity | acupoint thread embedding | sham acupuncture western medicine | subcutaneous indurations red and swollen |
| Hai YC, 2007 | China | 6 | participants with insomnia | auricular acupuncture | no treatment sham auricular acupuncture western medicine | NR |
| Hall ML, 2018 | New Zealand | 11 | patients with shoulder or upper extremity pain or dysfunction | dry needling therapy | sham acupuncture other therapy | mild treatment soreness lost consciousness |
| Han J, 2021 | China | 10 | functional dyspepsia | acupuncture electroacupuncture | sham acupuncture western medication | haematoma dizziness digestive system symptoms |
| Han X, 2021 | China | 9 | adult women (≥ 18 years) with stress urinary incontinence | electroacupuncture | sham electroacupuncture no intervention | subcutaneous hematoma fatigue pain discomfort  palpitation |
| Hao CZ, 2013 | China | 8 | participants with neurogenic bladder after spinal cord injury | acupuncture | other therapy | NR |
| He L, 2004 | China | 3 | patients with Bell’s palsy | acupuncture | sham acupuncture other therapy | NR |
| He L, 2007 | China | 6 | patients with Bell’s palsy | acupuncture | sham acupuncture other therapy | NR |
| He M,2016 | China | 5 | patients with unilateral or bilateral subjective tinnitus | electroacupuncture | western medicine Chinese medicine other therapy | NR |
| Heo I, 2013 | Korea | 16 | patients with spinal cord injury | acupuncture electroacupuncture auricular acupuncture | rehabilitation training sham acupuncture | NS |
| Hong QX, 2016 | China | 17 | patients with climacteric depression | acupuncture | western medicine | pain poor appetite insomnia |
| Hong S, 2020 | Korea | 10 | patients with stroke | acupuncture auricular acupuncture electroacupuncture | western medicine physical therapy sham acupuncture | NA |
| Hou WZ, 2019 | China | 13 | female patients (≥18years) with lymphedema | acupuncture | western medicine rehabilitation training usual care | NA bruises hematoma skin infection |
| Hou Z, 2017 | China | 10 | patients with cervical vertigo | acupuncture | no intervention sham acupuncture western medicine rehabilitation training | mild subcutaneous bruise skin allergy reaction NA |
| Hsieh PC, 2019 | China | 12 | patients with stable chronic obstructive pulmonary disease | body acupuncture therapy | western medicine rehabilitation training | NR |
| Hu CQ, 2016 | China | 20 | adult patients with cancer pain | acupuncture | sham acupuncture western medicine | subcutaneous bruises subcutaneous hemorrhage fainting |
| Hu H, 2019 | China | 33 | patients diagnosed with primary trigeminal neuralgia | manual acupuncture | western medicine | acupuncture syncope dizziness or drowsiness pigmentation near acupoints pain  NA |
| Hu HT, 2017 | China | 16 | adult patients (>18 years old) with low back pain | dry needling therapy | sham dry needling therapy physical therapy other therapy | needle sticking deterioration of symptoms dry needle-sticks |
| Hu XY, 2014 | UK | 2 | patients with phantom limb pain or phantom limb syndrome | acupuncture | western medicine physical therapy sham acupuncture | NR |
| Huang CW, 2020 | China | 6 | participants should be healthy | acupuncture | sham acupuncture no intervention | NR |
| Huang F, 2019 | China | 12 | patients with postmenopausal osteoporosis | acupoint thread embedding | sham acupoint thread embedding western medicine | fainting haematoma formation foreign-body reaction |
| Huang J,2020 | China | 16 | participants had poststroke dysphagia | electroacupuncture+ rehabilitation training | rehabilitation training | pain  hematoma |
| Huang JJ, 2021 | China | 61 | general population | acupoint thread embedding | other therapy | induration bleeding or bruising haematoma fever skin redness minor swelling pain local or systemic infection dizziness catgut sticking menoxenia neuromuscular disease |
| Huang KY, 2020 | China | 8 | patients with tinnitus | manual acupuncture | sham acupuncture | transient sedation vasovagal shock |
| Huang Q, 2019 | China | 18 | patients with diagnosis of Alzheimer’s Disease | acupuncture | sham acupuncture western medicine no intervention physical therapy | spot-bleeding pain |
| Huang W, 2009 | USA | 13 | patients with insomnia | acupuncture acupuncture + usual care | usual care western medicine sham acupuncture other therapy | local ecchymoses |
| Huang J,2021 | China | 30 | patients with acute or chronic stroke | scalp acupuncture | western medicine rehabilitation training | NS |
| Huh JH, 2021 | Korea | 7 | patients with mild-tomoderate carpal tunnel syndrome | manual acupuncture | other therapy | erythema  acupuncture ecchymosis local paresthesia |
| Hwang MS, 2020 | Korea | 13 | patients with chemotherapy-induced peripheral neuropathy | acupuncture | sham acupuncture western medicine | minor swelling bruising NA |
| Jan AL,2017 | Australia | 6 | patients with acute pain | ear acupuncture | sham acupuncture usual care | pain |
| Jang A, 2020 | Australia | 9 | patients with breast cancer patients, non-small cell lung cancerand various types of malignancy | true acupuncture | sham acupuncture usual care | spot bleeding or bruising local discomfort nausea dizziness |
| Jang S, 2020 | Korea | 3 | women with poor ovarian response | manual acupuncture ear acupuncture electroacupuncture warm acupuncture | sham electroacupuncture other therapy no intervention | mild allergies |
| Jang S, 2020 | Korea | 19 | women with breast cancer | manual acupuncture electroacupuncture | sham acupuncture western medicine rehabilitation training no intervention waitlist control | pain bleeding or bruising headache NA |
| Jedel E, 2005 | European | 3 | adult patients with xerostomia | manual acupuncture | other therapy  no intervention | tiredness |
| Jerng UM, 2014 | Korea | 4 | patients with oligozoospermia or asthenozoospermia | acupuncture acupuncture + other therapy | sham acupuncture other therapy | NA |
| Ji M, 2015 | China | 12 | patients with sciatica | manual acupuncture warm acupuncture electroacupuncture laser acupuncture | western medicine | subcutaneous hemorrhage NA |
| Jia W, 2020 | China | 7 | male with infertility | acupuncture | sham acupuncture western medicine  no intervention | fainting NA |
| Jiang YB, 2015 | China | 12 | patients with nerve deafness | acupuncture ear acupuncture electro-acupuncture acupuncture+ western medicine/Chinese medicine | western medicine Chinese medicine | NR |
| Jin Y, 2020 | China | 19 | patients with chemotherapy-induced peripheral neuropaty | acupuncture | sham acupuncture western medicine usual care | discomfort minor swelling bruising NA |
| Jo J, 2016 | Korea | 4 | women with polycystic ovarian syndrome | electroacupuncture manual acupuncture | no intervention | NR |
| Jo J, 2014 | Korea | 8 | women with primary ovarian insufficiency | acupuncture | western medicine sham acupuncture no intervention | NA bruises induration mild edema |
| Jo J, 2017 | Korea | 27 | participants with polycystic ovarian syndrome | acupuncture | sham acupuncture no intervention western medicine | back spasm redness hematomas dizziness  nausea mild bleeding nausea or vomiting pain  bleeding mild diarrhea dizziness or weakness NA |
| Ju ZY, 2017 | China | 6 | adults with chronic neuropathic pain | acupuncture | sham acupuncture other therapy | NS |
| Jung A, 2011 | Korea | 7 | participants with temporomandibular joint disorder | acupuncture | sham acupuncture | NA |
| Kim H, 2019 | Korea | 5 | participants with mild cognitive impairment | electroacupuncture | western medicine | NR |
| Kim JI, 2012 | Korea | 9 | patients with tinnitus | acupuncture | western medicine | pain |
| Kim JI, 2012 | Korea | 8 | patients with Bell's palsy | acupuncture | western medicine | bruising NA |
| Kim KH, 2013 | Korea | 6 | patients with lumbar spinal stenosis | acupuncture +other therapy | other therapy | NR |
| Kim KH, 2012 | Korea | 4 | patients with various musculoskeletal and non-musculoskeletal symptoms | acupuncture + western medicine | western medicine | pain bleeding light-headedness sweating itching erythema |
| Kim KH, 2010 | Korea | 6 | patients with end-stage renal disease | acupuncture  electroacupuncture | sham Acupuncture western medicine | elbow soreness minimal bleeding NA |
| Kim KH, 2018 | Korea | 32 | participants with symptomatic gastroparesis | acupuncture acupuncture + western medicine | western medicine | dizziness |
| Kim KH,2016 | Korea | 24 | adults with chronic kidney disease | manual acupuncture | sham manual acupuncture | elbow soreness NA |
| Kim KN, 2015 | Korea | 20 | patients with functional dyspepsia | acupuncture | sham acupuncture  western medicine no intervention | slight ecchymosis NA |
| Kim SH, 2019 | Korea | 19 | participants with primary insomnia | acupuncture | western medicine | needle-sickness |
| Kim SY, 2011 | Korea | 10 | women with premenstrual syndrome or premenstrual tension syndrome | acupuncture | sham acupuncture no intervention | subcutaneous haematoma hypomenorrhoea shortened menstrual cycle NA |
| Kim SY, 2018 | Korea | 27 | patients had BMI of ≥25 and were aged between 18 and 65 years | acupuncture electroacupuncture manual acupuncture auricular acupuncture | sham acupuncture  rehabilitation training no intervention other therapy | NA infammation pain dizziness nausea tiredness psychological disorder digestive system symptoms bleeding or bruising skin redness minor swelling pruritus induration anorexia palpitations haematoma |
| Kim TH, 2014 | Korea | 20 | adults with acute ankle sprains | acupuncture | no intervention sham acupuncture other therapy | NR |
| Kim YD, 2013 | Korea | 6 | patients with posttraumatic stress disorder | acupuncture auricular acupuncture electro-acupuncture | cognitive behavioral therapy waitlist control western medicine | pain |
| Ko GWY, 2019 | UK | 5 | patients with delayed-onset muscle soreness | acupuncture | sham acupuncture no intervention | NR |
| Ko HF, 2021 | China | 7 | patients with total knee replacement | acupuncture | sham acupuncture rehabilitation training | NA |
| Koog YH, 2014 | Korea | 58 | general population | acupuncture | sham acupuncture | NS |
| Kuang X, 2021 | China | 28 | patient with post-stroke cognitive impairment | electroacupuncture manual acupuncture | other therapy | subcutaneous mild haematoma fainting |
| Kwon CY, 2021 | Korea | 5 | people with dementia | electroacupuncture ear acupressure Anshen acupuncture | usual care western medicine | NA |
| Kwon CY, 2020 | Korea | 10 | participants with psychological trauma-related disorders after large-scale disasters | acupuncture | usual care western medicine | NA |
| Kwon CY, 2018 | Korea | 9 | participants with cognitive disorders | auricular acupuncture auricular acupuncture + western medicine auricular acupuncture+ Chinese medicine | western medicine Chinese medicine | NA |
| La Touche R, 2019 | China | 22 | participants with erectile dysfunction | acupuncture acupuncture + western medicine acupuncture + Chinese medicine Acupuncture+ psychological therapy | Chinese medicine western medicine sham acupuncture psychological therapy | facial red dizziness needle sticking pruritus NA |
| Lai XH, 2020 | China | 15 | women with stress urinary incontinence | electroacupuncture | western medicine sham electroacupuncture other therapy | pain subcutaneous haematoma  fatigue NA |
| Lam YC, 2008 | China | 10 | patients with idiopathic Parkinson’s disease | scalp/body electroacupuncture electroacupuncture +western medicine | western medicine | subcutaneous haematoma worsening of orthostasis |
| Lan J, 2020 | China | 21 | participants (≥45years) with vascular dementia | acupuncture | western medicine | fever mild pain  punctate hemorrhage hematoma needle sickness bleeding or bruising |
| Lan L, 2014 | China | 7 | patients (≥17years) with functional dyspepsia | manual acupuncture acupuncture | sham acupuncture western medicine | numb feeling subcutaneous hematoma |
| Langhorst, J, 2009 | Germany | 7 | patients with fibromyalgia syndrome | acupuncture | sham acupuncture western medicine no intervention | discomfort nausea soreness worsening of fibromyalgia |
| Lee A, 2014 | China | 59 | all surgical patients without age limitation | acupuncture electroacupuncture | sham acupuncture no intervention | NA haematomas pain redness and irritation feeling tired and sleepy erythema cutaneous irritation |
| Lee B, 2018 | Korea | 27 | children with autism spectrum disorder | manual acupuncture electroacupuncture | usual care other therapy | minor superficial bleeding crying irritability NA |
| Lee D, 2018 | Korea | 4 | patients (0-25 week) with sinfantile colic | acupuncture electroacupuncture | western medicine no intervention | slight bleeding |
| Lee H, 2003 | Korea | 6 | patients with GI endoscopic procedures | acupuncture | sham acupuncture western medicine no intervention | NA |
| Lee H, 2004 | Korea | 3 | parturient | acupuncture | usual care sham acupuncture | NA |
| Lee H, 2009 | Korea | 11 | patients with hypertensive | acupuncture manual acupuncture acupuncture + western medicine | sham acupuncture sham acupuncture + western medicine | hypertensive urgencies pain bleeding  NA |
| Lee H, 2016 | Korea | 7 | adults with either chronic or acute heart failure | manual acupuncture acupuncture + western medicine | sham acupuncture western medicine no intervention | NA |
| Lee H, 2013 | Korea | 11 | patients with acute/subacute nonspecific low back pain | acupuncture | sham acupuncture western medicine | hypodermal bleedings |
| Lee H, 2004 | UK | 7 | patients with pain in human cancer | ear acupuncture | sham ear acupuncture western medicine | NS |
| Lee HS, 2013 | Korea | 4 | patients with Parkinson's disease | scalp acupuncture+ western medicine | western medicine | pain |
| Lee JA, 2014 | Korea | 1 | patients with rheumatoid arthritis | bee venom acupuncture | sham bee venom acupuncture | NS |
| Lee JH, 2016 | Korea | 4 | adults with cancer | manual acupuncture  warm needling therapy fire needle acupuncture | sham acupuncture no intervention | NA |
| Lee JW, 2020 | Korea | 22 | sports injuries of athletes | manual acupuncture | other therapy | bleeding pain numbness NA |
| Lee MS, 2011 | UK | 11 | children with autism spectrum disorders | acupuncture | sham acupuncture no treatment | bleeding irritability symptoms worsen NA |
| Lee MS, 2008 | UK | 6 | patients with breast cancer | acupuncture | sham acupuncture | bleeding or bruising NA |
| Lee MS, 2009 | Korea | 6 | men with prostate cancer | manual acupuncture | sham manual acupuncture | distress fatigue hematoma increase of vasomotor symptoms |
| Lee MS, 2009 | Korea | 12 | allergic rhinitis | acupuncture manual acupuncture | western medicine sham acupuncture | pain bruising dizziness numbness headache NA |
| Lee MS, 2008 | Korea | 11 | patients with musculoskeletal pain | bee venom acupuncture | saline injection classic acupuncture saline injection + classic acupuncture | skin hypersensitivity itching Pain |
| Lee MS, 2011 | Korea | 6 | patients with dry eye | acupuncture | artificial tears | NA |
| Lee MS, 2008 | Korea | 9 | patients with rheumatoid arthritis | acupuncture | western medicine sham acupuncture | pain NA |
| Lee, MS, 2008 | Korea | 4 | patients with erectile dysfunction | acupuncture | sham acupuncture | NA |
| Lee MS, 2009 | Korea | 6 | women with hot flushes | acupuncture | sham acupuncture | NA bleeding skin rash pruritis insomnia pain itchiness twitching |
| Lee MS, 2008 | Korea | 11 | patients with Parkinson disease | Acupuncture scalp electroacupuncture | western medicine sham acupuncture | pain mild dry mouth nausea dizziness gastrointestinal symptoms |
| Lee MS, 2009 | Korea | 13 | patients with schizophrenia | manual acupuncture acupuncture + western medicine | sham acupuncture western medicine | pain dry mouth tremor insomnia blurred vision dizziness constipation nausea ⁄vomiting palpitation myotonia sweating headache numbness |
| Lee MS, 2008 | Korea | 10 | patients with insomnia | auricular acupuncture | sham acupuncture western medicine no treatment usual care | pain |
| Lee SH, 2016 | Korea | 13 | patients with insomnia after stroke | acupuncture | sham acupuncture western medicine | NR |
| Lee SH, 2016 | Korea | 12 | patients with post stroke shoulder pain | acupuncture + rehabilitation training | rehabilitation training | NR |
| Lee SJ, 2012 | Korea | 21 | patients with stroke | electro-scalp acupuncture  scalp acupuncture  scalp acupuncture + rehabilitation training scalp acupuncture +western medicine | western medicine rehabilitation training | death NA |
| Lew J, 2021 | USA | 6 | patients with neck and upper back myofascial pain syndrome | dry needling therapy | trigger point manual therapy | NR |
| Li D, 2019 | China | 8 | patients with herpes zoster | fire needling therapy | western medicine | NA |
| Li DZ, 2014 | China | 4 | patients with hypertension | acupuncture | sham acupuncture | hypertensive urgencies pain bleeding |
| Li HJ, 2021 | USA | 26 | breast cancer survivors | verum acupuncture | sham acupuncture waitlist control usual care other therapy | bruising or bleeding pain swelling skin infection hematoma headache |
| Li, JL, 2016 | China | 21 | patients with rheumatoid arthritis | electroacupuncture fire needling therapy acupuncture | western medicine Chinese medicine | NS |
| Li JL, 2021 | China | 14 | patients with osteoporotic vertebral compression fracture | acupuncture | other therapy  western medicine | NS |
| Li K, 2019 | USA | 3 | patients with chemotherapy-induced peripheral neuropathy | acupuncture | western medicine | swelling bruising |
| Li L, 2014 | China | 25 | adult patients (＞18 years) with hemiplegia | acupuncture + rehabilitation training/ conventional medicine | rehabilitation training western medicine usual care | transient fainting pain blood pressure dizziness superficial hematoma |
| Li LX, 2015 | China | 21 | children (6 to 14months) with cerebral palsy | acupuncture | sham acupuncture rehabilitation training | crying pain infections NA |
| Li M, 2021 | China | 7 | patients with recurrent implantation failure | acupuncture | other therapy  sham acupuncture | subcutaneous congestion |
| Li P, 2015 | UK | 14 | patients with Bell’s palsy | acupuncture | western medicine | NA |
| Li S, 2018 | China | 9 | women with postpartum depression | acupuncture | sham acupuncture Chinese medicine western medicine psychological intervention | fainting |
| Li SQ, 2022 | China | 21 | patients with type 2 diabetes mellitus | acupuncture  acupuncture + western medicine | other therapy  western medicine | pain nausea palpitations |
| Li W, 2020 | China | 34 | patients with insomnia | acupoint thread embedding | western medicine | NS |
| Li X, 2020 | China | 13 | patients with metabolic syndrome | acupuncture | sham acupuncture | bleeding or bruise |
| Li XR, 2014 | China | 1 | adults and children with allergic rhinitis | acupoint thread embedding | Chinese medicine | NA |
| Lian WL, 2014 | China | 33 | adults with cancer | acupuncture | western medicine sham acupuncture no intervention | bleeding or bruising NA |
| Liang S, 2017 | China | 12 | participants with chloasma | acupuncture acupuncture + western medicine | western medicine no intervention | NA |
| Lim B, 2006 | USA | 6 | adult subjects with irritable bowel syndrome | acupuncture  ear acupuncture | sham acupuncture western medication | NA abdominal pain defecation difficulties diarrhea alternating diarrhea constipation bloating |
| Lim CED, 2019 | Australia | 8 | women with polycystic ovarian syndrome | acupuncture  electroacupuncture | sham acupuncture no intervention rehabilitation training western medicine | NS |
| Lin JG, 2011 | China | 10 | participants with opiate/heroin dependence | acupuncture | NR | slight bleeding mild nausea dizziness dry mouth |
| Liu AF, 2020 | China | 17 | participants with acute ankle sprain | Acupuncture | no intervention sham acupuncture western medicine usual care | NA |
| Liu AJ, 2015 | China | 18 | patients with acute ischemic stroke | electroacupuncture+ western medicine | western medicine | acupuncture site ecchymosis NA |
| Liu C, 2019 | China | 14 | participants (＜18years) with autism spectrum disorders | scalp acupuncture | rehabilitation training usual care | NR |
| Liu F, 2016 | China | 18 | patients with tinnitus | acupuncture | sham acupuncture western medicine Chinese medicine | minor pain |
| Liu L, 2022 | China | 10 | patients with (＞18years) sacroiliac joint malposition | acupuncture | rehabilitation training other therapy | mild subcutaneous hematoma NA |
| Liu P, 2021 | China | 26 | patients with gouty arthritis | acupuncture | western medicine | NR |
| Liu SN, 2019 | China | 38 | participant with post-stroke shoulder-hand syndrome | acupuncture+ rehabilitation training | rehabilitation training | bruising NA |
| Liu W, 2020 | China | 22 | patients with post stroke cognitive impairment | acupuncture eye acupuncture acupuncture + western medicine/ usual care | usual care western medicine | NA subcutaneous hematoma fainting |
| Liu XM, 2021 | China | 7 | patients (>18years) with breast cancer | body/auricular acupuncture electroacupuncture acupuncture acupuncture + western medicine | sham acupuncture western medicine | bruising presyncope NA |
| Liu XJ, 2020 | China | 10 | women (20-45 years) with luteinized unruptured follicle syndrome | abdominal acupuncture acupuncture | chinese herb  western medicine | NA |
| Liu XL, 2015 | China | 59 | adult (age ≥ 18 years) with pain following any surgical procedure | acupuncture electroacupuncture manual acupuncture | usual care sham acupuncture no intervention | pain discomfort  bruising or bleeding mild burning  erythema fatigue |
| Liu Y, 2022 | China | 7 | women with polycystic ovary syndrome | acupuncture electroacupuncture acupuncture/electroacupuncture + western medicine | sham acupuncture western medicine | subcutaneous bruising hematoma NA |
| Liu Yuan, 2019 | China | 12 | patients with stable angina pectoris | acupuncture acupuncture + western medicine | western medicine | NA |
| Liu Yun, 2019 | China | 22 | infertile women without undergoing assisted reproductive techniques | acupuncture acupuncture + western medicine | western medicine | NA |
| Liu YH, 2017 | China | 10 | participants (≥18years) with cancer | manual acupuncture electroacupuncture | no intervention sham acupuncture Chinese medicine | NA |
| Llurda-Almuzara L, 2021 | Spain | 6 | adult population (>18 years old) with plantar heel pain or plantar fasciitis | dry needling | sham acupuncture no intervention | post-needling soreness bleeding or bruising |
| Long Z, 2022 | China | 3 | adult female patients with mixed urinary incontinence | electroacupuncture  acupuncture | western medication | mild subcutaneous hematoma fatigue worsened incontinence NA |
| Lu H, 2021 | China | 16 | patients with hyperemesis gravidarum | acupuncture  acupuncture + symptomatic rehydration support treatment | sham acupuncture western medicine no intervention  symptomatic rehydration support treatment | skin rash |
| Lu HL, 2022 | China | 12 | patients with rheumatoid arthritis | electroacupuncture electroacupuncture + western medicine acupuncture | west medicine | tingling sensation abnormal sensations in the throat loss of appetite bloating subcutaneous hematoma mild liver dysfunction leukopenia |
| Lu W, 2009 | USA | 11 | patients with cancer | acupuncture | west medicine | NR |
| Lv ZT, 2016 | China | 6 | participants with obstructive sleep apnea | acupuncture | no intervention sham acupuncture | NA |
| Lu ZT, 2016 | China | 7 | patients with chondromalacia patellae | acupuncture | west medicine | NA |
| Ma H, 2014 | China | 6 | women with polycystic ovary syndrome | acupuncture acupuncture + western medicine | western medicine | NS |
| Ma R, 2016 | China | 3 | participants (18-75years) with anxiety disorder | acupuncture | western medicine | NA |
| Mak TC, 2017 | China | 7 | participants with overactive bladder | acupuncture | acupuncture sham acupuncture western medicine | bleeding or bruising pain |
| Ziuk K, 2012 | Canada | 7 | participants with cerebral palsy | acupuncture | sham acupuncture usual care | fear minor pain |
| Manheimer E, 2010 | USA | 16 | participants with osteoarthritis | acupuncture | sham intervention waitlist control other therapy | bruising or bleeding |
| Manheimer E, 2012 | USA | 17 | adult participants with irritable bowel syndrome | acupuncture | sham acupuncture other therapy | syncope |
| Manheimer E, 2018 | USA | 6 | participants with hip or knee osteoarthritis or both hip and knee osteoarthritis | acupuncture | sham acupuncture no intervention | bruising or bleeding pain |
| Manheimer E, 2007 | USA | 11 | participants with knee osteoarthritis | acupuncture | sham control waitlist control usual care | NS |
| Manheimer E, 2013 | USA | 16 | pregnancy among women with vitro fertilization | acupuncture | sham acupuncture no intervention | NR |
| Manheimer E, 2012 | USA | 17 | participants with irritable bowel syndrome | acupuncture | sham acupuncture no intervention western medicine | syncope |
| Manheimer E, 2008 | USA | 7 | women with vitro fertilisation | acupuncture | sham acupuncture no intervention | NS |
| Mansu SSY, 2018 | Australia | 12 | participants with acne vulgaris | acupuncture auricular acupuncture electroacupuncture | western medicine no intervention sham acupuncture | NS |
| Mao X, 2020 | China | 7 | patients with functional dyspepsia | electroacupuncture | western medicine sham electroacupuncture | hemorrhage faint marasmus NA |
| Mills EJ, 2005 | Canada | 9 | patients with cocaine dependence | acupuncture | sham acupuncture | pain fear of needles |
| Moon TW, 2014 | Korea | 6 | patients with whiplash associated disorder | acupuncture | usual care sham acupuncture | bruising fatigue slight pain sweating low blood pressure |
| Murakami M, 2017 | USA | 10 | participants with pain | ear acupuncture  acupuncture + western medicine | western medicine | local pain bleeding headache |
| Naguit N, 2021 | USA | 15 | participants (≥18years) with migraine | acupuncture | western medicine sham acupuncture no intervention waitlist control | hemorrhage subcutaneous hematoma ecchymosis leg weakness pain local paresthesia |
| Namazi N, 2017 | Tehran | 7 | participants with obese adults | laser acupuncture | sham laser acupuncture | NR |
| Navarro-Santana MJ,  2020 | Spain | 28 | adults(≥18years) with neck pain symptoms | dry needling therapy | sham dry needling therapy no intervention physical therapy | post-needling soreness NA |
| Navarro-Santana MJ, 2022 | Spain | 6 | participants with neck pain | dry needling therapy | prigger point injection (wet needling) | post-needling soreness muscle pain discomfort paresthesia fatigue headache hemorrhage transient flare dizziness |
| Navarro-Santana MJ,  2020 | Spain | 7 | adults with lateral epicondylalgia of musculoskeletal origin | dry needling | sham acupuncture no intervention | local hemorrhage |
| Ni XX, 2020 | China | 8 | patients with cancer | acupuncture | sham acupuncture usual care | NA |
| Noh H, 2017 | Korea | 42 | patients with idiopathic Parkinson’s disease | acupuncture acupuncture +western medicine electroacupuncture +western medicine | sham acupuncture western medicine | gastrointestinal symptoms (nausea, vomiting, constipation, and anorexia) pain subcutaneous hematocele itchiness motor disorders dry mouth NA |
| O'Sullivan EM, 2020 | UK | 3 | patients with head and neck cancer | acupuncture | sham acupuncture | NA |
| Ou L,2021 | China | 11 | older adults with hip and knee arthroplasty | electroacupuncture | no intervention sham electroacupuncture | nausea/vomiting pulmonary infection NA |
| Paley CA, 2011 | UK | 3 | adults with cancer-related pain | acupuncture | sham acupuncture western medicine | NR |
| Pan H, 2018 | China | 35 | patients with primary osteoporosis and postmenopausal osteoporosis | warming acupuncture electroacupuncture | western medicine | NR |
| Pan YQ, 2018 | China | 17 | patients with breast cancer | acupuncture electroacupuncture | sham acupuncture no intervention western medicine | NR |
| Pang B, 2016 | China | 16 | participants (＞17 years) functional dyspepsia | acupuncture | sham acupuncture | electric shock or tingling |
| Park J, 2013 | Korean | 17 | patients with ankle sprains | acupuncture | sham acupuncture no intervention usual care | NA |
| Park J, 2001 | UK | 9 | patients with stroke | acupuncture | sham acupuncture rehabilitation training usual care | dizziness |
| Park J, 2013 | Korea | 105 | pregnant women | acupuncture | sham acupuncture no intervention usual care | pain bleeding or bruising haematoma tiredness headache drowsiness dizziness discomfort  nausea heat or sweating faint rash  itching blood pressure |
| Park JY, 2017 | Korea | 15 | general population | acupuncture | sham acupuncture | fainting hemorrhage |
| Park KS, 2017 | Korea | 8 | obese patients | auricular acupuncture acupuncture + usual care | sham auricular acupuncture  no intervention usual care | minor inflammation mild tenderness dizziness NA |
| Park S, 2020 | Korea | 11 | patients with thoracotomy | electroacupuncture | sham electroacupuncture western medicine | NA |
| Park YJ, 2020 | Korea | 15 | adults with stroke | Acupuncture + rehabilitation training | rehabilitation training | pain |
| Posadzki P, 2013 | UK | 7 | patients with cancer | acupuncture electroacupuncture acupuncture usual care | sham acupuncture no intervention usual care | nausea stomach ache bleeding or bruising feeling of discomfort |
| Posadzki P, 2011 | Korea | 9 | adults with chronic prostatitis/chronic pelvic pain syndrome | acupuncture | western medicine Chinese medicine | hematomas pain NA |
| Pourahmadi M,2021 | USA | 11 | patients with headache | dry needling therapy | sham acupuncture western medicine | discomfort pain fear |
| Qin X, 2020 | China | 5 | women (≥18years) with uncomplicated recurrent urinary tract infection | manual  acupuncture | sham acupuncture western medicine  no intervention | gastrointestinal discomfort frequent menstruation dizziness |
| Qin Z, 2015 | China | 11 | patients with sciatica | acupuncture acupuncture + western medicine | western medicine  sham acupuncture | hypodermal bleeding NA |
| Qin ZS, 2016 | China | 7 | participants with chronic prostatitis/ chronic pelvic pain syndrome | acupuncture acupuncture + western medicine | western medicine  sham acupuncture | hematomas pain  NA |
| Qiu X, 2021 | China | 16 | patients with limb cramps after stroke | fire acupuncture | acupuncture | NR |
| Qu F, 2016 | UK | 9 | patients with polycystic ovarian syndrome | auricular acupuncture electroacupuncture warm needling therapy | sham acupuncture sham acupuncture+ western medicine/ Chinese medicine no intervention | NR |
| Rahou-El-Bachiri Y,  2020 | Spain | 10 | adults with knee pain of musculoskeletal origin | dry needling therapy | sham acupuncture no intervention | post-needling soreness hemorrhages NA |
| Rathbone J, 2005 | UK | 5 | patients with schizophrenia, chizophreniform psychosis and schizophrenia-like illnesses | acupuncture + western medicine | western medicine | NA |
| Roberts J, 2008 | UK | 7 | patients with allergic rhinitis | acupuncture | sham acupuncture usual care | NR |
| Seo SY, 2017 | Korea | 16 | adults with chronic neck pain | acupuncture electroacupuncture | usual care physical therapy western medicine | aggravation of symptoms dizziness tiredness |
| Shen FJ, 2019 | China | 13 | participants with migraine | acupuncture | western medicine | sedation bleeding local pain ecchymosis |
| Shen L, 2020 | Korea | 7 | patients with shoulder pain | bee venom acupunture | saline injection | pruritus burning sensation pain local swelling redness |
| Sheng J, 2019 | China | 15 | patients with abdominal obesity | acupoint catgut embedding | usual care other therapy | fainting subcutaneous indurations hematoma bruise |
| Shergis JL, 2016 | Australia | 30 | participants with insomnia | acupuncture | sham acupuncture western medicine | bruise headache dizziness fatigue muscle convulsion fainting hand numbness pain |
| Sim H, 2011 | Korea | 6 | patients with carpal tunnel syndrome | acupuncture laser acupuncture | sham laser acupuncture western medicine other therapy | NA |
| Skjeie H, 2018 | Norway | 3 | infantile colic | acupuncture | sham acupuncture usual care no intervention | minor bleeding hiccups increased regurgitation |
| Smith CA, 2010 | UK | 30 | adults with depression | manual acupuncture | wait list control sham acupuncture other therapy  western medicine | sleep disturbances headaches tiredness palpitations dryness of the mouth NA |
| Smith CA, 2010 | Australia | 10 | patients with primary dysmenorrhoea | acupuncture | sham acupuncture western medicine Chinese medicine usual care | tiredness pain  headache bleeding needling pain aggravation of symptoms |
| Sniezek DP, 2013 | USA | 6 | women with anxiety and depression | manual acupuncture | sham acupuncture other therapy usual care no intervention | pain bleeding |
| Sorbero M, 2015 | USA | 18 | participants with major depressive disorder | acupuncture acupuncture + western medicine | sham acupuncture western medicine | pain discomfort mild bleeding or bruises sleep disturbances palpitations fainting |
| ern C, 2016 | UK | 15 | participants with alcohol dependence | acupuncture | sham acupuncture western medicine usual care | pain mild bleeding agitation drowsiness |
| Stub T, 2011 | China | 4 | adults with depression | acupuncture | sham acupuncture western medicine | fatigue |
| Su IJ, 2021 | China | 10 | adults with post-stroke depression | acupuncture acupuncture + western medicine | other therapy  western medicine usual care | local pain bruising fainting |
| Su X, 2021 | China | 13 | participants with (≥18 years) acute low back pain | acupuncture | sham acupuncture western medicine | NA |
| Sun Y, 2008 | USA | 31 | chronic headache in adult patients | acupuncture | sham acupuncture western medicine | bleeding or bruising local paraesthesia headache |
| Sun Y, 2008 | USA | 15 | adults with postoperative pain | acupuncture | sham acupuncture | bleeding headache local pain dizziness discomfort instantaneous bradycardia |
| Sung SH, 2021 | Korea | 11 | patients with temporomandibular disorders | electroacupuncture  electroacupuncture +usual care | no intervention sham acupuncture usual care | NR |
| Sung SH, 2018 | Korea | 4 | women with chronic pelvic pain | auricular acupuncture electroacupuncture warm needling therapy | physical therapy western medicine | NA |
| Sung WS, 2020 | Korea | 18 | patients with lumbar herniated intervertebral disc | acupoint thread embedding | other therapy | NA |
| Tan J, 2021 | China | 10 | patients with spinal cord injury | electroacupuncture | rehabilitation training western medicine | NR |
| Tang ECH, 2021 | China | 9 | patients with cognitive impairment associated with cerebral small vessel disease | acupuncture | western medicine Chinese medicine usual care rehabilitation training | NA |
| Tang HZ, 2015 | China | 4 | adult participants (≥18 years old) with lateral epicondylitis | acupuncture acupuncture +physical therapy | sham acupuncture physical therapy | pain NA |
| Tang S, 2017 | China | 30 | patients with lumbar discherniation | acupuncture | western medicine usual care no intervention | local haematoma NA |
| Thiagarajah A, 2017 | Singapore | 4 | patients with pain caused by plantar fasciitis | acupuncture/electroacupuncture+ conventional treatment | sham acupuncture western medicine other therapy | headaches dizziness soreness mild oedema bruising |
| Tong QY, 2021 | China | 12 | adult preoperative anxiety inpatients or outpatients | acupuncture | sham acupuncture no intervention usual care | nausea vomiting NA |
| Trigkilidas D, 2010 | UK | 4 | patients with chronic low back pain | acupuncture | usual care | pain dizziness back spasm |
| Trinh K, 2021 | Canada | 1 | patients with any foot or ankle disorder | dry needling electroacupuncture | sham acupuncture waitlist control | NA |
| Trinh K, 2022 | Canada | 10 | patients with hand- or wrist-related condition | acupuncture | sham acupuncture waitlist control no treatment | NA |
| Trinh KV, 2016 | Canada | 27 | adults (18 years or older) with neck disorders | acupuncture | sham acupuncture wait-list control no intervention | pain bruising fainting worsening of symptoms local swelling dizziness |
| Tu M, 2021 | China | 7 | participants with chronic stable angina pectoris | acupuncture | sham acupuncture usual care | local subcutaneous  bleeding pain numbness |
| Tu Y, 2022 | China | 27 | patients with flaccid hemiplegia | acupuncture electroacupuncture acupuncture/ electroacupuncture + western medicine/ rehabilitation training | western medicine rehabilitation training | NR |
| Urroz P, 2013 | Australia | 4 | adult participants with exercise performance and postexercise recovery | acupuncture electroacupuncture | sham acupuncture no intervention | NR |
| Valencia-Chulián R,  2020 | Spain | 16 | adult participants with hemiplegia or hemiparesis after stroke | dry needling therapy | sham acupuncture | loss of blood pressure feeling of heaviness tingling |
| Van den Heuvel E,  2016 | Belgium | 29 | women with nausea and vomiting and on hyperemesis in pregnancy | acupuncture | sham acupuncture western medicine Chinese medicine no intervention | NR |
| Van den Noort M, 2018 | Korea; | 26 | patients with schizophrenia | acupuncture | western medicine | NA |
| Von Trott P, 2020 | UK | 12 | adult participants (>18 years) with breathlessness | acupuncture | sham acupuncture no intervention usual care | NS |
| Wang J, 2018 | China | 19 | patients with chronic obstructive pulmonary disease | acupuncture | sham acupuncture western medicine Chinese medicine no intervention | fatigue subcutaneous hemorrhage dizziness needle site pain NA |
| Wang J, 2013 | China | 35 | patients with hypertension | acupuncture acupuncture + western medicine | western medicine sham acupuncture western medicine + sham acupuncture usual care | pain bleeding NA |
| Wang J, 2016 | China | 3 | patients with chronic urinary retention | acupuncture +  rehabilitation training/other therapy | rehabilitation training other therapy | NR |
| Wang JH, 2019 | China | 24 | participants with smoking cessation | electroacupuncture auricular acupuncture acupuncture | sham electroacupuncture sham acupuncture no intervention waitlist control | NA bleeding or bruising fainting pain anorexia weeping headache dizziness nausea sensation |
| Wang L, 2021 | China | 16 | children with autism spectrum disorder | acupuncture acupuncture + rehabilitation training | rehabilitation training sham acupuncture | subcutaneous bruise bleeding cried irritability |
| Wang L, 2019 | China | 9 | adults with obstructive sleep apnea | acupuncture electroacupuncture | no treatment other therapy  Chinese medicine western medicine | NA |
| Wang LQ, 2018 | China | 14 | patients with diabetic peripheral neuropathy | acupoint injection | western medicine | NS |
| Wang M, 2015 | China | 30 | participants with underwent elective or emergent abdominal surgery | acupoint injection | usual care other therapy | NA |
| Wang P, 2022 | China | 7 | patients with dysphagia | acupuncture acupuncture + rehabilitation treatment | rehabilitation treatment | pneumonia asphyxia dehydration pain |
| Wang R, 2017 | China | 10 | patients with myofascial pain syndrome | manual acupuncture | no intervention sham Acupuncture | discomfort bruises |
| Wang TT, 2020 | China | 18 | patients with knee osteoarthritis | acupuncture | sham acupuncture other therapy | NS |
| Wang WH, 2020 | China | 23 | patients with peripheral facial paralysis | electroacupuncture | other therapy | NS |
| Wang XM, 2018 | China | 15 | patients with postpartum urinary retention | acupuncture electroacupuncture | western medicine | NA |
| Wang XP, 2018 | China | 18 | patients with hot flashes in breast cancer | acupuncture electroacupuncture | sham acupuncture western medicine | NA |
| Wang Y, 2018 | China | 7 | patients with postherpetic neuralgia | acupuncture electroacupuncture | western medicine | NR |
| Wang Y, 2012 | China | 8 | patients with acute ischemic stroke | scalp acupuncture | western medicine | NR |
| Wang Y, 2013 | China | 1 | patients with a diagnosis of stress urinary incontinence | electroacupuncture scalp acupuncture body acupuncture | western medicine | NA |
| Wei X, 2019 | China | 13 | patients with reflex sympathetic dystrophy after stroke | electroacupuncture electroacupuncture + rehabilitation training /other therapy | rehabilitation training other therapy | NR |
| Wen X, 2021 | China | 66 | participants with Parkinson’s disease | acupuncture /electroacupuncture /scalp electroacupuncture +western medicine | western medicine | NA |
| Wong ISY, 2012 | China | 9 | patients with dysphagia after stroke | acupuncture + rehabilitation training electroacupuncture | rehabilitation training | NR |
| Wong V, 2012 | China | 4 | patients with traumatic brain injury | acupuncture + western medicine/ rehabilitation training | western medicine /rehabilitation training | NR |
| Woo HL, 2018 | Korea | 60 | women with primary dysmenorrhea | manual acupuncture electroacupuncture auricular acupuncture warm needling therapy acupoint thread embedding | sham acupuncture western medicine no intervention | discomfort hemorrhage headache myalgia fever dizziness NA |
| Wu HM, 2009 | China | 5 | patients with stroke in the subacute or chronic stage | acupuncture acupuncture + western medicine acupuncture + rehabilitation training | western medicine sham acupuncture + rehabilitation training | NR |
| Wu IX, 2019 | China | 10 | patients with carpal tunnel syndrome | manual acupuncture electroacupuncture acupuncture manual acupuncture acupuncture + rehabilitation training | sham acupuncture western medicine rehabilitation training | local pain ecchymosis local paresthesia local dermatitis bruising numbness tingling NA |
| Wu LQ, 2019 | China | 20 | patients with insulin resistance | acupuncture acupuncture+western medicine/sham acupuncture /lifestyle modification | western medicine sham acupuncture lifestyle modification | mild pain subcutaneous haematoma NA |
| Xiang A, 2017 | China | 13 | participants with nonspecific pain | acupuncture | analgesic injection sham acupuncture no intervention | hematoma NA |
| Xiang Y, 2017 | China | 14 | adults (>18 years) with non-specific low back pain | acupuncture | sham acupuncture | minor haematoma bleeding |
| Xiao X, 2020 | China | 11 | participants with chronic obstructive pulmonary disease | acupuncture + rehabilitation training acupuncture + western medicine | western medicine rehabilitation training | mild contusion pain NA |
| Xie G, 2020 | China | 13 | patients with nonspecific chronic low back pain | acupoint injection | sham acupoint injection rehabilitation training | skin flair edema skin rash |
| Xing M, 2019 | China | 10 | patients with moderate-severe acne | fire needling therapy fire needling therapy + western medicine | western medicine | burning itching redness swelling of the skin |
| Xiong J, 2021 | China | 11 | patients with cough-variant asthma | acupuncture acupuncture + western medicine acupuncture + rehabilitation training | western medicine rehabilitation training | gastrointestinal reactions local skin redness NA |
| Xiong W, 2016 | China | 11 | patients with diabetic peripheral neuropathy | electroacupuncture electroacupuncture+ western medicine | western medicine | NS |
| Xu G, 2021 | China | 6 | patients (aged ≥18 years) with defecation dysfunction | acupuncture electropuncture | western medicine rehabilitation training other therapy | NR |
| Xuan Y, 2021 | China | 23 | participants with primary dysmenorrhea | acupuncture | western medicine | NA |
| Xuan YC, 2020 | China | 10 | participants with ankylosing spondylitis | acupuncture | western medicine usual care | NA |
| Xue P, 2016 | China | 18 | participants with asthma | acupoint injection acupoint injection+ usual care | western medicine usual care | local skin  redness swelling pain |
| Yan B, 2020 | China | 7 | patients diagnosed with chronic pain combined with depression or depression combined with chronic pain | acupuncture acupuncture+ western medicine | western medicine | NA |
| Yang B, 2013 | China | 9 | participants with fibromyalgia syndrome | acupuncture electroacupuncture | sham acupuncture western medicine | NA |
| Yang C, 2018 | China | 19 | participants with shoulder adhesive capsulitis | acupuncture | sham acupuncture western medicine rehabilitation training | subcutaneous hematoma |
| Yang J, 2021 | China | 41 | stroke patients diagnosed with insomnia | acupuncture | western medicine | NA |
| Yang J, 2018 | China | 22 | adults with primary hypertension | acupuncture | sham acupuncture western medicine | headache pain spot-bleeding |
| Yang J, 2020 | China | 9 | participants (≥18years) with pain | battlefield acupuncture battlefield acupuncture + other therapy | other therapy  no intervention sham battlefield acupuncture+ other therapy | discomfort pain redness irritation |
| Yang L, 2015 | China | 7 | participants with sjogren’s syndrome | acupuncture | artificial tears | hematoma pain NA |
| Yang M, 2020 | China | 13 | participants with menstrual migraine | acupuncture | sham acupuncture western medicine | bleeding or bruising subcutaneous haematoma tingling sensation pain |
| Yang MX, 2013 | China | 14 | participants with diabetic gastroparesis | acupuncture | sham acupuncture western medicine | NR |
| Yang T, 2020 | China | 17 | participants with non-dialysis dependent chronic kidney disease | acupoint injection | no intervention shame acupoint injection | erythema itching numbness swelling pain NA |
| Yang XY, 2021 | China | 20 | participants with anxiety disorder | acupuncture electroacupuncture | other therapy  western medicine sham acupuncture usual care | pain hematoma faintness bleeding |
| Yang Y, 2020 | China | 9 | participants with cancer pain | auricular acupuncture  auricular therapy+ western medicine | sham auricular acupuncture  western medicine | NA |
| Yao JP, 2019 | China | 12 | weight loss in Asians | acupuncture electroacupuncture acupuncture + lifestyle intervention | sham acupuncture lifestyle intervention no intervention | inflammation mild tenderness mild ecchymosis abdominal discomfort |
| Yao Q, 2016 | China | 6 | participants with chronic urticaria | acupuncture acupuncture + Chinese medicine /western medicine | Chinese medicine/ western medicine | local skin hemorrhage feeling faint gastrointestinal discomfort menstrual disorders nervous feeling |
| Ye JY, 2021 | China | 12 | participants with anxiety and/or depression during in vitro fertilization treatment | acupuncture | western medicine no intervention sham acupuncture | NA |
| Ye Q, 2017 | China | 70 | patients with stroke and dysphagia | acupuncture acupuncture + rehabilitation/usual care/west medicine/ other therapy | rehabilitation/basic treatment/west medicine/other therapy | subcutaneous hemorrhage needle sickness |
| Yeung WF, 2009 | China | 20 | participants with a chief complaint of insomnia | acupuncture | western medicine no intervention sham acupuncture | pain ecchymoses |
| Yi HM, 2020 | China | 14 | children with autism spectrum disorders | scalp acupuncture+ rehabilitation training | rehabilitation training | NR |
| Yin ZH, 2020 | China | 14 | patients (≥18years) with postcholecystectomy syndrome | acupuncture + western medicine | western medicine | dizziness  NA |
| You F, 2019 | China | 12 | males with oligoasthenozoospermia | acupuncture electroacupuncture manual acupuncture | sham acupuncture western medicine no intervention Chinese medicine | NA |
| You YN, 2018 | Korea | 21 | patients with stroke | scalp acupuncture | sham scalp acupuncture western medicine | NA |
| Yu C, 2015 | China | 25 | patients with angina pectoris | acupuncture acupuncture + west medicine/Chinese medicine | no intervention rehabilitation training west medicine/ Chinese medicine | NA |
| Yu CC, 2017 | China | 25 | women with polycystic ovary syndrome | acupoint thread embedding | west medicine/Chinese medicine | syncope subcutaneous bleeding  decreased appetite intolerable distention |
| Yu J, 2016 | China | 7 | patients with tourette syndrome | acupuncture acupuncture + west medicine | west medicine | NA |
| Yu S, 2020 | China | 8 | patients (18–85 years) with breast cancer | acupuncture warm needling therapy | western medicine usual care | pain  skin infections haematoma NA |
| Yuan J, 2008 | Northern Ireland | 23 | patients with nonspecific lowback pain | manual acupuncture  electroacupuncture acupuncture | no intervention sham acupuncture  rehabilitation training | NS |
| Yuan QL, 2016 | China | 63 | patients with pain associated with musculoskeletal disorders | acupuncture | sham acupuncture | pain nausea vomiting dizziness syncope |
| Yuan XX, 2017 | China | 31 | patients with functional dyspepsia | manual acupuncture | western medicine | subcutaneous hematoma dizziness headache mild diarrhea |
| Yue J, 2016 | China | 5 | patients with hiccups following stroke | acupuncture | western medicine | NR |
| Yun JM, 2020 | Korea | 11 | patients with occipital neuralgia | acupuncture acupuncture + western medicine | western medicine | NR |
| Zeng YC, 2014 | China | 7 | cancer survivors | acupuncture | sham acupuncture usual care no treatment/wait-list control | spot bleeding or bruising feeling discomfort nausea |
| Zhan J, 2017 | China | 19 | motor dysfunction in acute stroke survivors | electroacupuncture +  rehabilitation therapy/ western medicine | rehabilitation therapy  western medicine sham acupuncture no intervention | NR |
| Zhan J, 2021 | China | 7 | participants with stroke | abdominal acupuncture+ rehabilitation therapy  western medicine other therapy | rehabilitation therapy  western medicine other therapy | local subcutaneous ecchymosis |
| Zhang BL, 2019 | China | 28 | adults with post-stroke aphasia | acupuncture  scalp acupuncture | no intervention sham scalp acupuncture | NR |
| Zhang GC, 2012 | China | 15 | participants with post-stroke depression | acupuncture | west medicine | NA |
| Zhang J, 2019 | China | 15 | participants with premenstrual syndrome | acupuncture | sham acupuncture no intervention western medicine | hypomenorrhea pain  hematoma NA |
| Zhang J, 2014 | China | 17 | participants with post-stroke depression | filiform needle acupuncture | western medicine | pain |
| Zhang JH, 2012 | China | 3 | children (<18 years) with a history of asthma | laser acupuncture | sham laser acupuncture western medicine | NR |
| Zhang K, 2019 | China | 12 | participants with acute pancreatitis | acupuncture + other therapy | other therapy | pain local swelling bleeding NA |
| Zhang K, 2018 | China | 19 | obesity patients | acupuncture | sham acupuncture other therapy  no intervention | bleeding or bruising infammation dizziness NA |
| Zhang N, 2020 | China | 6 | participants with chronic severe functional constipation | electroacupuncture | sham electroacupuncture | needle detachment bleeding pain hematoma |
| Zhang N, 2019 | USA | 7 | participants with migraine | acupuncture | western medicine | mild bleeding ecchymosis local pain sedation fatigue |
| Zhang Q, 2019 | China | 16 | participants with chronic fatigue syndrome | acupuncture | sham acupuncture other therapy  no intervention usual care | redness  itching right thumb numbness  pain NA |
| Zhang RC, 2019 | China | 11 | participants with Bell’s palsy | acupuncture | west medicine | NS |
| Zhang T, 2013 | China | 15 | adult participants with constipation | electroacupuncture | other therapy | NA |
| Zhang W, 2017 | China | 8 | participants with benign prostatic hyperplasia | acupuncture | sham acupuncture | psychological disorder tiredness NA |
| Zhang X, 2019 | China | 13 | patients with myasthenia gravis | acupuncture | west medicine | slight dry mouth nausea myasthenic crisis |
| Zhang X, 2018 | China | 31 | patients with vitro fertilization or intracytoplasmic sperm injection | acupuncture acupuncture +other therapy | sham treatment other therapy  no intervention | inadequate comfort dizziness fatigue NA |
| Zhang X, 2018 | China | 6 | women (≥18 years) with breast cancer-related lymphoedema | acupuncture | waitlist control | bruising pain haematoma |
| Zhang XC, 2019 | China | 12 | participants with fibromyalgia | acupuncture | sham acupuncture  west medicine | bruising soreness nausea discomfort of needle insertion aggravation of symptoms |
| Zhang XC, 2015 | China | 12 | participants with sudden sensorineural hearing loss | acupuncture acupuncture + western medicine | no intervention sham acupuncture western medicine | a local skin infection NA |
| Zhang XW, 2018 | China | 20 | participants with Bell’s palsy | electroacupuncture manual acupuncture | other therapy | NS |
| Zhang XY, 2019 | China | 7 | adults (≥18 years) with post-stroke depression | acupuncture acupuncture + western medicine | western medicine | subcutaneous continuous pain dizziness nausea subcutaneous hematoma |
| Zhang Y, 2018 | China | 10 | adult cancer patients (≥ 18 years) | acupuncture electroacupuncture | sham acupuncture usual care | NR |
| Zhang Y, 2021 | China | 33 | adult females (age>=18 years) with breast cancer | acupuncture | sham acupuncture western medicine  no intervention usual care | pain bleeding or bruising fatigue pruritus dizziness NA |
| Zhang Y, 2020 | China | 14 | patients (≥18years) with poststroke aphasia | acupuncture+  rehabilitation training | rehabilitation training no intervention sham acupuncture | NA |
| Zhang Z, 2015 | China | 8 | participants with angina pectoris | acupuncture | western medicine | NR |
| Zhao FY, 2021 | China | 15 | women in the perimenopausal period with a clinical diagnosis of primary insomnia | acupuncture acupuncture + west medicine | west medicine | hematoma mild dizziness mild tension |
| Zhao FY, 2021 | China | 11 | patients with primary insomnia | manual acupuncture electroacupuncture | waitlist control sham acupuncture | needle site bruising hematoma headache  needle site pain hand numbness  worsening of insomnia  NA |
| Zhao L, 2011 | China | 16 | patients with neurovascular headache | acupuncture | west medicine | headache |
| Zhao QY, 2021 | China | 12 | patients with urinary retention after hysterectomy | electroacupuncture+ rehabilitation training | rehabilitation training | NS |
| Zhao XF, 2015 | China | 23 | patients with hypertension | acupuncture acupuncture + western medicine/ lifestyle | sham acupuncture western medicine lifestyle | spot-bleeding  hypertensive urgencies congestive heart failure pain |
| Zhao YW, 2018 | China | 10 | participants(≥18years) with overactive bladder | acupuncture acupuncture + western medicine | sham acupuncture western medicine | minor pain subcutaneous bruises |
| Zheng CH, 2012 | China | 24 | women with vitro fertilization | manual acupuncture electroacupuncture laser acupuncture | no intervention sham acupuncture | NR |
| Zheng GQ, 2011 | China | 7 | patients with acute hypertensive intracerebral hemorrhage | acupuncture electroacupuncture | usual care | NA |
| Zheng H, 2019 | China | 41 | patients with irritable bowel syndrome | acupuncture acupuncture + western medicine/ Chinese medicine | western medicine Chinese medicine usual care | needle fainting |
| Zheng RQ, 2021 | China | 10 | patients with polycystic ovary syndrome | manual acupuncture electroacupuncture | sham acupuncture other therapy | mild bleeding NA |
| Zheng XZ, 2021 | China | 9 | female patients with gynecologic surgery | acupuncture | sham acupuncture | redness erythema swelling allergy |
| Zhi FY, 2019 | China | 9 | patients with optic atrophy | manual acupuncture manual acupuncture+ western medicine | western medicine | NR |
| Zhong L, 2021 | China | 35 | patients with dysphagia after stroke | acupuncture + other therapy | other therapy | bleeding  pain discomfort |
| Zhong Y, 2018 | China | 23 | patients with urinary retention | electroacupuncture  electroacupuncture + usual care | sham acupuncture no intervention usual care | local hematoma  local muscle convulsion urinary system infection NA |
| Zhong Y, 2019 | China | 13 | patients with low endometrial receptivity | acupuncture acupuncture + conventional therapy | conventional therapy (routine treatment, western medicine,  sham acupuncture or no intervention) | fainting NA |
| Zhong YJ, 2020 | China | 10 | women with stress urinar incontinence | electroacupuncture | sham elactroacupuncture western medicine | pain hematoma bruise fatigue tingling palpitation |
| Zhong YM, 2019 | China | 8 | participants (≥18years) with obesity | electroacupuncture auricular acupuncture | sham acupuncture  no intervention | bleeding dizziness minor inflammation mild tenderness |
| Zhou J, 2015 | China | 10 | patients with Alzheimer disease | acupuncture  acupuncture + western medicine | western medicine no intervention | hemorrhage of local skin fatigue feeling faint drowsy feelings  nausea dizziness nausea |
| Zhou L, 2020 | China | 37 | patients (≥18 years) with cognitive impairment of post-stroke | acupuncture electroacupuncture | sham acupuncture rehabilitation training | NR |
| Zhou M, 2009 | China | 6 | participants with Bell’s palsy | acupuncture | sham acupuncture other therapy  no intervention | NR |
| Zhou WM, 2016 | China | 24 | patients with functional dyspepsia | acupuncture | sham acupuncture western medicine | mild fainting pain or hematoma at acupoints mild gastrointestinal distention |
| Zhu F, 2021 | China | 19 | patients with acute pancreatitis | acupuncture electroacupuncture warm acupuncture ear acupuncture | usual care sham acupuncture | subcutaneous hemorrhage  NA |
| Zhu J, 2016 | China | 12 | patients (18-70years) with gastro-oesophageal reflux disease | manual acupuncture electroacupuncture acupuncture + western medicine | western medicine | mild wrist pain |
| Zhu X, 2011 | Australia | 24 | women with endometriosis | auricular acupuncture | Chinese medicine | NR |
| Zhuang L, 2012 | China | 4 | patients with radiation-induced xerostomia | acupuncture | usual care sham acupuncture | tiny hemorrhage |
| Zheng J, 2022 | China | 14 | females with chronic pelvic pain syndrome | acupuncture + rehabilitation training | rehabilitation training | NS |
| Kai-feng D, 2022 | China | 10 | patients with gouty arthritis | fire needling therapy | western medicine | gastric discomforts skin rashes  pruritus |
| Hou Y, 2020 | China | 9 | participants with unilateral neglect after stroke | acupuncture+ rehabilitation training | rehabilitation training | NA |
| Li, LX, 2019 | China | 17 | participants with ischaemic or haemorrhagic stroke | acupuncture + rehabilitation training | rehabilitation training | pain mild vomiting ecchymosis haematoma |
| Huang T, 2011 | China | 4 | children (<16years) with nocturnal enuresis | acupuncture | west medicine sham acupuncture | NA |
| Lee SW, 2017 | China | 7 | diabetes patients | acupuncture | sham acupuncture usual care | mild hypoglycemia  NA |
| Chan YY, 2015 | China | 13 | patients with depression | manual acupuncture electroacupuncture auricular acupuncture laser acupuncture acupuncture + west medicine | west medicine | NS |
| Jiang HL, 2020 | China | 18 | patients with diabetic peripheral neuropathy | manual acupuncture  acupuncture + west medicine | west medicine | NA |
| Liu TT, 2009 | China | 21 | participants with opioid– heroin dependence | acupuncture + west medicine | west medicine | nausea vomiting dry mouth blurred vision headache muscle twitching |
| Li Y, 2017 | USA | 13 | patients with cardiac arrhythmia | acupuncture acupuncture + west medicine | west medicine | NR |
| Lingling Y, 2018 | China | 15 | patients with postpartum depression | acupuncture electroacupuncture | sham acupuncture west medicine | discomfort at the needle site headache  needle site pain bruising at the needle site  dizziness |
| Xie Q, 2020 | China | 30 | adults with dysarthria following stroke | acupuncture + rehabilitation training | rehabilitation training | slight pain subcutaneous hemorrhage |
| Wong V, 2013 | China | 4 | patients with traumatic brain injury | acupuncture/electroacupuncture + west medicine | west medicine rehabilitation training | NR |
| Wang X, 2021 | China | 24 | patients with post-stroke depression | acupuncture + western medicine | western medicine | nausea  abdominal distension dry mouth constipation intermittent headaches decreased appetite sleep disturbance |
| Wen X, 2021 | China | 66 | patients with Parkinson’s disease | acupoint thread embedding acupuncture + western medicine /Chinese medicine | western medicine/Chinese medicine | NA |
| Zhang K, 2021 | China | 13 | patients with post-stroke depression | acupuncture + western medicine | western medicine | physical fatigue  fainting |
| Yang J, 2021 | China | 10 | participants with poststroke thalamic pain | acupuncture | western medicine | NS |
| You J, 2021 | China | 8 | participants with depression and chronic pain | electroacupuncture manual acupuncture | western medicine | NS |
| Zhang J,2020 | China | 15 | patients with insomnia | acupuncture electroacupuncture | sham acupuncture | hematoma  headache dizziness sharp needling pain nausea insomnia hand numbness |
| Zhang Q,2017 | China | 19 | patients with chronic knee pain | acupuncture acupuncture + usual care | western medicine usual care no intervention | NS |
| Xu M,2018 | China | 33 | patients with acute stroke | acupuncture | sham acupuncture | NS |
| wei Z,2104 | China | 13 | patients with post-stroke depression | acupuncture electroacupuncture | western medicine | NS |
| Zhang Y, 2020 | China | 29 | patients with flat warts | acupuncture fire needle therapy | Chinese medicine western medicine | erythema blisters infection itching pain mild burning pigmentation desquamation scar isomorphic response |
| Zhang ZJ, 2010 | China | 207 | patients with depressive disorders | fire needling therapy fire needling therapy+ western medicine/Chinese medicine | western medicine/ Chinese medicine | NS |
| Zheng C, 2022 | China | 13 | patients with fibromyalgia | electroacupuncture manual acupuncture | sham acupuncture other therapy | bruising nausea fainting |
| Zhou L, 2022 | China | 26 | patients with stroke and insomnia | acupuncture | western medicine | NS |
| Zhu LB, 2014 | China | 33 | participants with pain symptoms | wrist-ankle acupuncture | western medicine | dizziness nausea subcutaneous hemorrhage |
| Xu J,2018 | China | 14 | patients with migraine | acupuncture | sham acupuncture western medicine | periorbital ecchymosis fainting local pain |
| Xu Y, 2017 | China | 16 | patients with primary dysmenorrhoea | acupuncture | western medicine | NS |
| Yang J, 2021 | China | 19 | participants with post-stroke thalamic pain | electroacupuncture acupuncture | western medicine | NS |
| Yang M, 2019 | China | 17 | patients with stable angina pectoris | electroacupuncture manual acupuncture | sham acupuncture western medicine usual care | NS |
| Zheng J, 2022 | China | 14 | female patients with chronic pelvic pain syndrome | acupuncture + rehabilitation training | rehabilitation training | NS |
| Deng K, 2022 | China | 10 | patients with gouty arthritis | fire needling therapy | western medicine | discomfort skin rashes pruritus |
| Fan SQ, 2021 | China | 20 | participants (≥18years) with migraine | acupuncture | sham acupuncture western medicine | NS |
| Fei Y, 2019 | China | 8 | patients with atrial fibrillation | acupuncture electroacupuncture | western medicine | NS |
| Guo J, 2020 | China | 31 | adults with diarrhea-predominant irritable bowel syndrome or functional diarrhea | acupuncture electroacupuncture eye acupuncture warm needling therapy | sham acupuncture western medicine | NS |
| He C, 2017 | China | 7 | adult patients with plantar heel pain | dry needling therapy acupuncture | sham acupuncture usual care | pain |
| Hou XB, 2020 | China | 19 | patients with cancer pain | acupuncture electroacupuncture ear acupuncture wrist and ankle acupuncture | western medicine other therapy | NS |
| Höxtermann MD, 2021 | Germany | 65 | oncological patients | electroacupuncture | no intervention western medicine sham acupuncture | bleeding hematoma pain vegetative symptoms |
| Huang JF, 2020 | China | 22 | participants with chronic spinal pain | acupuncture electroacupuncture fire needling therapy auricular acupuncture abdominal acupuncture warm acupuncture bee venom acupuncture | usual care sham acupuncture western medicine | pain |
| Wang L, 2022 | China | 24 | adults with functional gastrointestinal disorders | acupuncture electroacupuncture manual acupuncture | sham acupuncture western medicine | NS |
| Wang L, 2020 | China | 28 | patients (>18 years) with functional constipation | acupuncture | sham acupuncture western medicine | NS |
| Wang X, 2020 | China | 13 | participants (≥18 years) ulcerative colitis | acupuncture acupuncture + western medicine | western medicine | nausea/vomiting dizziness mild gastrointestinal discomfort spasmodic myalgia constipation |
| Wang XF, 2021 | China | 19 | patients with poststroke depression | electroacupuncture | western medicine | fainting subcutaneous haemorrhage pain nausea |
| Lan Y, 2015 | China | 15 | patients (18-80years) with dissatisfaction about sleep quality | auricular acupuncture | sham acupuncture western medicine | auricle pain using magnetic pearls experienced redness |
| Li P, 2020 | China | 10 | patients with functional constipation | acupuncture | western medicine | NS |
| Li X, 2017 | China | 18 | patients with post-stroke depression | electroacupuncture | western medicine | NS |
| Mu J, 2020 | China | 33 | adults (≥18 years) with chronic nonspecific low back pain | acupuncture | sham acupuncture usual care | NS |
| Ou MQ, 2020 | China | 28 | patients with migraine | acupuncture electroacupuncture | western medicine sham acupuncture | NS |
| Pei W, 2019 | China | 8 | participants with postherpetic neuralgia | acupuncture | sham acupuncture western medicine | NS |
| Quan K, 2022 | China | 27 | adult patients with infertility | acupuncture | no intervention sham acupuncture | local pain bleeding or bruising pruritus |
| Smith CA, 2018 | Australia | 64 | patients with depression | acupuncture manual acupuncture electroacupuncture laser acupuncture | sham acupuncture western medicine | dizziness aches fatigue vagueness nausea |
| Smith CA, 2018 | Australia | 20 | women with embryo transfer | acupuncture | sham acupuncture no intervention | nausea dizziness fainting tiredness drosiness headache pain itching bruising |
| Smith CA, 2016 | Australia | 42 | women (15-49 years) with primary dysmenorrhoea | acupuncture | western medicine usual care | NS |
| Su XT, 2021 | China | 48 | patients with vascular cognitive impairment | manual acupuncture electroacupuncture acupuncture | western medicine usual care | NS |
| Tao B, 2015 | China | 19 | patients with functional constipation | acupuncture | western medicine Chinese medicine | NS |
| Xi-yang Wang, 2021 | China | 61 | adult (18–75 years) with functional gastrointestinal disorders | acupuncture acupuncture + other therapy | sham acupuncture western medicine | broken needles nausea syncope severe pain haematoma bleeding numbness infection  insomnia/dizziness/ discomfort |
| Wang Y, 2020 | China | 30 | patients with Alzheimer's disease | acupuncture  acupuncture + western medication | western medicine | NS |
| Xiao X, 2020 | China | 16 | perimenopausal women diagnosed with depression | acupuncture  manual electroacupuncture | western medication | NS |
| Linde K, 2016 | Germany | 12 | adult participants with tension-type headache | acupuncture | usual care waitlist control sham acupuncture | NS |
| Linde K, 2018 | Germany | 22 | participants with migraine | acupuncture | no acupuncture sham acupuncture western medicine | NS |
| Linde K, 2010 | Germany | 22 | participants with migraine | acupuncture | no acupuncture sham acupuncture western medicine | NS |
| Liu B, 2016 | China | 10 | patients with chronic prostatitis/chronic pelvic pain syndrome | acupuncture | sham acupuncture western medicine | NS |
| Liu F, 2019 | China | 9 | diagnosed with chronic pain-related insomnia | acupuncture | western medicine sham acupuncture | soreness local bruising nausea |
| Liu F, 2021 | China | 21 | patients with insomnia | scalp acupuncture head acupuncture | western medicine other therapy  no intervention | NS |
| Liu R, 2021 | China | 17 | patients with post-stroke depression | acupuncture electroacupuncture | western medicine sham acupuncture | NS |
| Lu C, 2021 | China | 22 | children (≤18 years) Tic disorders | manual acupuncture electroacupuncture | western medicine | NS |
| Lu W, 2016 | China | 28 | patients with gouty arthritis | manual acupuncture electroacupuncture | western medicine | gastrointestinal tract reaction central nervous system reaction leukopenia skin rash fainting |
| Luo Y, 2020 | China | 47 | patients with vitiligo | fire needle therapy | other therapy  western medicine Chinese medicine | burning sensation local redness itching of the skin |
| Manyanga T, 2014 | Canada | 12 | adults with osteoarthritis | acupuncture | sham acupuncture usual care no intervention | pain sleepiness fainting nausea localized swelling |
| Armour M,2019 | Australia | 29 | patients with depression | manual acupuncture electroacupuncture laser acupuncture | sham acupuncture usual care psychological intervention | NS |
| Bae H,2014 | Korea | 14 | patients with preoperative anxiety | acupuncture | sham acupuncture | NS |
| Cao H,2009 | China | 46 | patients with insomnia | body acupuncture auricular acupuncture electroacupuncture acupoint injection | sham acupuncture western medicine Chinese medicine no intervention | mild headache lassitude |
| Cao H,2019 | China | 73 | patients with primary insomnia | manual acupuncture electroacupuncture scalp acupuncture wrist–ankle acupuncture eye acupuncture | sham acupuncture western medicine no intervention | fainting bleeding dizziness skin flushing |
| Chen H,2018 | China | 30 | patients with essential hypertensive | acupuncture electroacupuncture  acupuncture + lifestyle modifications /western medicine | sham acupuncture western medicine lifestyle modifications no intervention | headache syncope dizziness pain cough bleeding |
| Jiang Y,2018 | China | 62 | participants with migraine | acupuncture | sham acupuncture western medicine no intervention | NS |
| Kong DZ,2019 | China | 8 | patients with chronic hepatitis B | acupuncture | sham acupuncture no intervention | NS |
| Chan-Young Kwon,2021 | Korea | 12 | patients with functional dyspepsia | acupuncture acupuncture + western medicine | western medicine | NS |

# Table S2: Detailed evaluation of the methodological quality with AMSTAR 2

| **Author, year** | **Q1** | **Q2** | **Q3** | **Q4** | **Q5** | **Q6** | **Q7** | **Q8** | **Q9** | **Q10** | **Q11** | **Q12** | **Q13** | **Q14** | **Q15** | **Q16** | **All** |
| --- | --- | --- | --- | --- | --- | --- | --- | --- | --- | --- | --- | --- | --- | --- | --- | --- | --- |
| Abdi F, 2021 | Yes | Yes | No | Yes | Yes | Yes | PY | Yes | Yes | No | NM | NM | No | No | NM | Yes | Critically low |
| Amorim D, 2018 | Yes | No | No | PY | Yes | Yes | No | Yes | No | No | NM | NM | Yes | No | NM | Yes | Critically low |
| Ang L, 2020 | Yes | Yes | No | Yes | Yes | Yes | PY | Yes | Yes | No | NM | NM | Yes | No | NM | Yes | Critically low |
| Ang L, 2021 | Yes | No | Yes | PY | Yes | Yes | Yes | PY | Yes | No | NM | NM | No | No | NM | No | Critically low |
| Ba J, 2013 | Yes | No | No | PY | Yes | Yes | PY | Yes | Yes | No | Yes | Yes | Yes | Yes | Yes | Yes | Critically low |
| Bae K, 2015 | Yes | No | No | PY | Yes | Yes | PY | Yes | Yes | No | NM | NM | Yes | No | NM | Yes | Critically low |
| Yang B, 2014 | Yes | No | No | PY | Yes | Yes | PY | Yes | Yes | No | Yes | Yes | Yes | Yes | No | Yes | Critically low |
| Bai ZH, 2015 | Yes | Yes | No | PY | Yes | Yes | PY | Yes | Yes | No | NM | NM | Yes | No | NM | Yes | Critically low |
| Park SW, 2014 | Yes | No | No | PY | Yes | Yes | PY | Yes | No | No | Yes | Yes | Yes | Yes | Yes | Yes | Critically low |
| Baviera AF, 2019 | Yes | No | No | Yes | Yes | Yes | PY | Yes | No | No | NM | NM | Yes | No | NM | No | Critically low |
| Befus D, 2018 | Yes | Yes | No | Yes | Yes | Yes | PY | Yes | Yes | No | Yes | Yes | Yes | Yes | No | Yes | Critically low |
| Ben-Arie E, 2020 | Yes | Yes | No | Yes | Yes | Yes | PY | Yes | Yes | No | Yes | Yes | Yes | Yes | Yes | Yes | Low |
| Blanco-Díaz M, 2022 | Yes | Yes | No | Yes | Yes | Yes | Yes | PY | Yes | No | NM | NM | No | No | NM | Yes | Critically low |
| Bonomo P, 2022 | Yes | No | No | Yes | Yes | Yes | Yes | Yes | Yes | No | NM | NM | No | No | NM | Yes | Critically low |
| Bower WF, 2005 | Yes | No | No | Yes | Yes | Yes | PY | Yes | Yes | No | Yes | Yes | Yes | No | Yes | No | Critically low |
| Cai YY, 2017 | Yes | No | No | Yes | Yes | Yes | PY | Yes | Yes | No | Yes | Yes | Yes | Yes | Yes | Yes | Critically low |
| Cao HJ, 2013 | Yes | No | No | PY | Yes | Yes | PY | Yes | Yes | No | Yes | Yes | Yes | Yes | Yes | Yes | Critically low |
| Cao JP, 2018 | Yes | No | No | Yes | Yes | Yes | Yes | Yes | Yes | No | Yes | Yes | Yes | Yes | Yes | Yes | Low |
| Cao L, 2012 | Yes | No | No | PY | Yes | Yes | PY | Yes | Yes | No | Yes | No | Yes | No | No | No | Critically low |
| Casimiro L, 2010 | Yes | Yes | No | Yes | Yes | Yes | Yes | Yes | Yes | No | Yes | Yes | Yes | Yes | Yes | Yes | Moderate |
| Chai QYC, 2015 | Yes | Yes | No | Yes | Yes | Yes | Yes | Yes | Yes | No | NM | NM | No | Yes | NM | Yes | Critically low |
| Chan YT, 2021 | Yes | Yes | No | Yes | Yes | Yes | PY | Yes | Yes | No | Yes | Yes | Yes | Yes | Yes | Yes | Low |
| Chang H, 2020 | Yes | Yes | No | Yes | Yes | Yes | PY | Yes | Yes | No | Yes | Yes | Yes | Yes | No | Yes | Critically low |
| Chang SC, 2016 | No | No | No | PY | Yes | Yes | PY | Yes | Yes | No | Yes | Yes | Yes | Yes | Yes | Yes | Critically low |
| Chao GQ, 2014 | Yes | No | No | Yes | Yes | Yes | Yes | Yes | Yes | No | Yes | Yes | Yes | Yes | Yes | Yes | Low |
| Chau JPC, 2018 | Yes | No | No | Yes | Yes | Yes | PY | Yes | Yes | No | NM | NM | Yes | No | NM | No | Critically low |
| Chen C, 2019 | Yes | Yes | No | Yes | Yes | Yes | Yes | Yes | Yes | No | Yes | Yes | Yes | Yes | Yes | Yes | Moderate |
| Chen H, 2020 | Yes | PY | No | Yes | Yes | Yes | PY | Yes | Yes | No | Yes | No | Yes | Yes | Yes | Yes | Low |
| Chen H, 2022 | Yes | Yes | No | Yes | Yes | Yes | PY | Yes | Yes | No | NM | NM | Yes | Yes | NM | Yes | Critically low |
| Chen HY, 2007 | No | No | No | PY | Yes | Yes | PY | Yes | Yes | No | Yes | Yes | Yes | Yes | No | No | Critically low |
| Chen JR, 2018 | Yes | Yes | No | Yes | Yes | Yes | PY | Yes | Yes | No | Yes | Yes | Yes | Yes | Yes | Yes | Low |
| Chen JF, 2021 | Yes | Yes | No | Yes | Yes | Yes | PY | Yes | Yes | Yes | Yes | Yes | Yes | Yes | Yes | Yes | Low |
| Chen J, 2012 | Yes | No | No | Yes | Yes | Yes | PY | Yes | Yes | No | Yes | Yes | No | Yes | Yes | No | Critically low |
| Chen LC, 2017 | Yes | Yes | No | Yes | Yes | Yes | Yes | Yes | Yes | No | Yes | No | Yes | Yes | No | Yes | Low |
| Chen N, 2017 | Yes | No | No | Yes | Yes | Yes | PY | Yes | Yes | No | Yes | Yes | Yes | Yes | Yes | No | Critically low |
| Chen N, 2010 | Yes | Yes | No | Yes | Yes | Yes | Yes | Yes | Yes | No | NM | NM | Yes | Yes | NM | Yes | Critically low |
| Chen P, 2021 | Yes | No | No | Yes | Yes | Yes | PY | Yes | Yes | No | Yes | Yes | Yes | Yes | No | No | Critically low |
| Chen SW, 2018 | Yes | No | No | Yes | Yes | Yes | PY | Yes | Yes | No | Yes | Yes | Yes | Yes | No | Yes | Critically low |
| Chen WJ, 2021 | Yes | No | No | Yes | Yes | Yes | PY | Yes | Yes | No | Yes | Yes | Yes | Yes | Yes | Yes | Critically low |
| ChenW, 2013 | Yes | Yes | No | Yes | Yes | Yes | PY | Yes | Yes | No | NM | NM | Yes | No | NM | Yes | Critically low |
| Chen XM, 2022 | Yes | No | No | Yes | Yes | Yes | PY | Yes | Yes | No | Yes | Yes | Yes | Yes | Yes | Yes | Critically low |
| Chen XT, 2022 | Yes | PY | No | Yes | Yes | Yes | PY | Yes | Yes | No | Yes | Yes | Yes | Yes | No | Yes | Critically low |
| Chen YC, 2021 | Yes | No | No | PY | Yes | Yes | PY | Yes | Yes | No | Yes | Yes | Yes | Yes | Yes | Yes | Critically low |
| Chen YP, 2016 | Yes | No | No | PY | Yes | Yes | PY | Yes | Yes | No | Yes | Yes | Yes | Yes | Yes | Yes | Critically low |
| Chen YP, 2021 | Yes | No | No | Yes | Yes | Yes | PY | Yes | Yes | No | Yes | No | No | Yes | Yes | Yes | Critically low |
| Chen Z, 2021 | Yes | No | No | Yes | Yes | Yes | No | Yes | Yes | No | Yes | Yes | Yes | Yes | Yes | Yes | Critically low |
| ChenZH, 2018 | Yes | Yes | No | Yes | Yes | Yes | Yes | Yes | Yes | No | Yes | Yes | Yes | Yes | Yes | Yes | Moderate |
| Cheng K, 2017 | Yes | Yes | No | Yes | Yes | Yes | Yes | Yes | Yes | Yes | Yes | Yes | Yes | Yes | Yes | Yes | High |
| Cheong KB, 2013 | Yes | PY | No | Yes | Yes | Yes | PY | Yes | Yes | No | Yes | Yes | Yes | Yes | Yes | Yes | Low |
| Cheuk DKL, 2014 | Yes | Yes | No | Yes | Yes | Yes | Yes | Yes | Yes | No | Yes | Yes | Yes | Yes | Yes | Yes | Moderate |
| Cheuk DKL, 2011 | Yes | Yes | No | Yes | Yes | Yes | PY | Yes | Yes | No | Yes | Yes | Yes | Yes | Yes | Yes | Low |
| Cheuk DKL, 2009 | Yes | Yes | No | Yes | Yes | Yes | Yes | Yes | Yes | No | Yes | Yes | Yes | Yes | Yes | Yes | Moderate |
| Chien TJ, 2017 | Yes | No | No | PY | Yes | Yes | PY | Yes | Yes | No | Yes | No | Yes | No | Yes | Yes | Critically low |
| Chien TJ, 2015 | Yes | No | No | Yes | Yes | Yes | PY | Yes | Yes | No | Yes | No | Yes | No | No | Yes | Critically low |
| Chien TJ, 2019 | Yes | No | No | PY | Yes | Yes | PY | Yes | Yes | No | Yes | No | Yes | No | Yes | Yes | Critically low |
| Chien TJ, 2019 | Yes | No | No | PY | Yes | Yes | PY | Yes | Yes | No | Yes | Yes | Yes | No | Yes | Yes | Critically low |
| Chiu HY, 2016 | Yes | No | No | Yes | Yes | Yes | PY | Yes | Yes | No | Yes | Yes | Yes | Yes | Yes | Yes | Critically low |
| Chiu H, 2016 | Yes | No | No | PY | Yes | Yes | PY | Yes | Yes | No | Yes | Yes | Yes | Yes | Yes | Yes | Critically low |
| Chiu HY, 2014 | Yes | No | No | PY | Yes | Yes | Yes | Yes | Yes | No | Yes | No | Yes | No | Yes | Yes | Critically low |
| ChoKH, 2017 | Yes | No | No | Yes | Yes | Yes | PY | Yes | Yes | No | NM | NM | Yes | Yes | NM | Yes | Critically low |
| Cho SH, 2010 | Yes | No | No | Yes | Yes | Yes | Yes | Yes | Yes | No | Yes | No | Yes | Yes | Yes | Yes | Low |
| Cho SH, 2010 | Yes | No | No | Yes | Yes | Yes | Yes | Yes | Yes | No | Yes | Yes | Yes | Yes | Yes | Yes | Low |
| Cho SH, 2010 | Yes | No | No | Yes | Yes | Yes | PY | Yes | Yes | No | Yes | No | Yes | Yes | No | Yes | Critically low |
| Cho SH, 2008 | Yes | No | No | PY | Yes | Yes | Yes | Yes | Yes | No | Yes | Yes | Yes | Yes | Yes | Yes | Critically low |
| Cho SH, 2009 | Yes | No | No | Yes | Yes | Yes | PY | Yes | Yes | No | Yes | No | Yes | Yes | Yes | Yes | Critically low |
| Cho SH, 2009 | Yes | No | No | Yes | Yes | Yes | Yes | Yes | Yes | No | Yes | Yes | Yes | Yes | Yes | Yes | Low |
| Cho WC, 2018 | Yes | No | No | Yes | No | Yes | PY | Yes | Yes | No | Yes | No | Yes | No | Yes | Yes | Critically low |
| Cho YH, 2014 | Yes | No | No | PY | Yes | Yes | Yes | Yes | Yes | No | Yes | No | Yes | Yes | No | Yes | Critically low |
| Choi GH, 2019 | Yes | Yes | Yes | Yes | Yes | Yes | Yes | Yes | Yes | Yes | Yes | Yes | Yes | Yes | Yes | Yes | High |
| Choi TY, 2016 | Yes | Yes | No | Yes | Yes | Yes | PY | Yes | Yes | No | Yes | No | Yes | Yes | No | Yes | Critically low |
| Choi TY, 2012 | Yes | No | No | Yes | Yes | Yes | PY | Yes | Yes | No | Yes | No | Yes | No | No | Yes | Critically low |
| Choi TY, 2012 | Yes | No | No | Yes | Yes | Yes | Yes | Yes | Yes | No | Yes | Yes | Yes | Yes | No | Yes | Critically low |
| Chung SY, 2016 | Yes | No | No | PY | Yes | Yes | PY | Yes | Yes | No | Yes | No | Yes | Yes | Yes | Yes | Critically low |
| Clark RJ, 2015 | No | No | No | Yes | No | No | PY | Yes | Yes | Yes | NM | NM | Yes | Yes | NM | Yes | Critically low |
| Cooper K, 2016 | Yes | Yes | No | Yes | Yes | Yes | Yes | Yes | Yes | No | Yes | No | Yes | No | Yes | Yes | Moderate |
| Hou XB, 2020 | Yes | No | No | Yes | Yes | Yes | PY | Yes | Yes | No | Yes | Yes | Yes | Yes | Yes | Yes | Critically low |
| Coyle MH, 2015 | Yes | No | No | PY | Yes | Yes | PY | Yes | Yes | No | NM | NM | Yes | No | NM | Yes | Critically low |
| Coyle MH, 2020 | Yes | No | No | PY | Yes | Yes | PY | Yes | Yes | No | Yes | Yes | Yes | Yes | No | Yes | Critically low |
| Cui W, 2018 | Yes | No | No | Yes | Yes | Yes | PY | Yes | Yes | No | Yes | No | Yes | Yes | No | Yes | Critically low |
| Cui XM, 2016 | Yes | Yes | No | Yes | Yes | Yes | PY | Yes | Yes | No | NM | NM | Yes | Yes | NM | Yes | Critically low |
| Cui Y, 2022 | Yes | Yes | No | Yes | Yes | Yes | PY | Yes | Yes | No | Yes | Yes | Yes | Yes | Yes | Yes | Low |
| Dai L, 2021 | Yes | Yes | No | Yes | Yes | Yes | PY | Yes | Yes | No | Yes | Yes | Yes | Yes | Yes | Yes | Low |
| Dai L, 2019 | Yes | Yes | No | Yes | Yes | Yes | PY | Yes | Yes | No | Yes | Yes | Yes | Yes | Yes | Yes | Low |
| Davis MA, 2008 | Yes | No | No | PY | Yes | Yes | PY | Yes | Yes | No | Yes | No | Yes | Yes | Yes | Yes | Critically low |
| Deare JC, 2013 | Yes | Yes | Yes | Yes | Yes | Yes | Yes | Yes | Yes | Yes | Yes | Yes | Yes | Yes | Yes | Yes | High |
| Deng M, 2016 | Yes | No | No | PY | Yes | Yes | PY | Yes | Yes | No | Yes | No | Yes | Yes | No | Yes | Critically low |
| Dimitrova A, 2017 | Yes | No | No | PY | Yes | Yes | Yes | Yes | Yes | No | Yes | Yes | Yes | Yes | Yes | Yes | Critically low |
| Dodin S, 2013 | Yes | Yes | No | Yes | Yes | Yes | Yes | Yes | Yes | Yes | Yes | Yes | Yes | Yes | Yes | Yes | High |
| Dong B, 2017 | Yes | No | No | PY | Yes | Yes | PY | Yes | Yes | No | Yes | No | Yes | Yes | Yes | Yes | Critically low |
| Dong Be, 2021 | Yes | No | No | Yes | Yes | Yes | PY | Yes | Yes | No | Yes | Yes | Yes | Yes | Yes | Yes | Critically low |
| Ee CC, 2007 | Yes | No | No | PY | Yes | Yes | PY | Yes | Yes | No | NM | NM | Yes | No | NM | Yes | Critically low |
| Ernst E, 1999 | Yes | No | No | PY | Yes | Yes | PY | Yes | No | No | NM | NM | No | No | NM | No | Critically low |
| Ezzo J, 2001 | Yes | No | No | PY | Yes | Yes | No | Yes | PY | No | NM | NM | Yes | No | NM | No | Critically low |
| Ezzo J, 2014 | Yes | Yes | No | Yes | Yes | Yes | Yes | Yes | Yes | No | Yes | Yes | Yes | Yes | Yes | Yes | Moderate |
| Fan WJ, 2020 | Yes | Yes | No | Yes | Yes | Yes | Yes | Yes | Yes | No | Yes | Yes | Yes | Yes | Yes | Yes | Moderate |
| Fang SJ, 2017 | Yes | No | No | PY | Yes | Yes | PY | Yes | No | No | Yes | No | No | Yes | Yes | No | Critically low |
| Farag AM, 2020 | Yes | Yes | No | Yes | Yes | Yes | Yes | Yes | Yes | No | Yes | Yes | Yes | Yes | Yes | Yes | Moderate |
| Feng SY, 2015 | Yes | No | No | PY | Yes | Yes | Yes | Yes | Yes | No | Yes | Yes | Yes | Yes | Yes | Yes | Critically low |
| Fernandez-de-las-Penas C, 2021 | Yes | Yes | No | PY | Yes | Yes | PY | Yes | Yes | No | Yes | Yes | Yes | Yes | Yes | Yes | Low |
| Fernández-Jané C, 2020 | Yes | Yes | No | Yes | Yes | Yes | Yes | Yes | Yes | No | Yes | Yes | Yes | Yes | No | Yes | Low |
| Fern, 2019 | Yes | Yes | No | Yes | Yes | Yes | Yes | Yes | Yes | No | Yes | Yes | Yes | Yes | Yes | Yes | Moderate |
| France S, 2014 | Yes | No | No | PY | Yes | Yes | PY | Yes | PY | No | NM | NM | Yes | No | NM | Yes | Critically low |
| Fu LM, 2009 | Yes | No | No | PY | Yes | Yes | PY | Yes | No | No | Yes | Yes | Yes | Yes | No | Yes | Critically low |
| Furlan AD, 2005 | Yes | Yes | No | Yes | Yes | Yes | Yes | Yes | Yes | Yes | Yes | Yes | Yes | Yes | Yes | Yes | High |
| Gao X, 2021 | Yes | Yes | No | Yes | Yes | Yes | Yes | Yes | Yes | No | Yes | Yes | Yes | Yes | Yes | Yes | Moderate |
| Garcia MK, 2016 | No | No | No | PY | Yes | Yes | PY | Yes | Yes | No | NM | NM | Yes | No | NM | Yes | Critically low |
| Gates S, 2006 | Yes | Yes | No | Yes | Yes | Yes | Yes | Yes | Yes | No | Yes | Yes | Yes | Yes | No | Yes | Low |
| Ge SQ, 2020 | Yes | No | No | Yes | Yes | Yes | Yes | Yes | Yes | No | Yes | Yes | Yes | Yes | Yes | Yes | Low |
| Giovanardi GM, 2020 | Yes | No | No | Yes | Yes | Yes | PY | Yes | Yes | Yes | Yes | Yes | Yes | Yes | Yes | Yes | Critically low |
| Grant S, 2017 | Yes | Yes | No | Yes | Yes | Yes | PY | Yes | Yes | No | Yes | Yes | Yes | Yes | Yes | Yes | Low |
| Grant S, 2016 | Yes | No | No | Yes | Yes | Yes | Yes | Yes | Yes | No | Yes | Yes | Yes | Yes | Yes | Yes | Low |
| Green S, 2013 | Yes | Yes | No | Yes | Yes | Yes | Yes | Yes | Yes | No | Yes | Yes | Yes | Yes | Yes | Yes | Moderate |
| Sally Green, 2008 | Yes | Yes | No | Yes | Yes | Yes | Yes | Yes | Yes | No | Yes | Yes | Yes | Yes | Yes | Yes | Moderate |
| Guo TP, 2015 | Yes | Yes | No | Yes | Yes | Yes | PY | Yes | PY | No | Yes | No | Yes | Yes | Yes | Yes | Low |
| Hai YC, 2007 | Yes | No | No | Yes | Yes | Yes | No | Yes | Yes | No | Yes | Yes | Yes | Yes | Yes | No | Critically low |
| Hall ML, 2018 | Yes | Yes | No | Yes | Yes | Yes | No | Yes | Yes | No | Yes | No | Yes | No | No | No | Critically low |
| Han J, 2021 | Yes | No | No | Yes | Yes | Yes | PY | Yes | Yes | No | Yes | No | Yes | Yes | No | No | Critically low |
| Han X, 2021 | Yes | Yes | No | Yes | Yes | Yes | PY | Yes | Yes | No | Yes | Yes | Yes | Yes | Yes | Yes | Low |
| Hao CZ, 2013 | Yes | No | No | Yes | Yes | Yes | Yes | Yes | Yes | No | Yes | No | Yes | Yes | Yes | Yes | Low |
| He L, 2004 | Yes | Yes | No | Yes | Yes | Yes | Yes | Yes | Yes | No | NM | NM | Yes | Yes | NM | No | Critically low |
| He L, 2007 | Yes | Yes | No | Yes | Yes | Yes | Yes | Yes | Yes | No | NM | NM | Yes | Yes | NM | No | Critically low |
| He M,2016 | Yes | No | No | Yes | Yes | Yes | Yes | Yes | Yes | No | NM | NM | Yes | No | NM | Yes | Critically low |
| Heo I, 2013 | Yes | No | No | Yes | Yes | Yes | PY | Yes | Yes | No | Yes | No | Yes | No | Yes | Yes | Critically low |
| Hong QX, 2016 | Yes | No | No | Yes | Yes | Yes | PY | Yes | Yes | No | Yes | Yes | No | Yes | Yes | Yes | Critically low |
| Hong S, 2020 | Yes | Yes | No | Yes | Yes | Yes | PY | Yes | Yes | No | Yes | Yes | Yes | Yes | Yes | Yes | Low |
| Hou WZ, 2019 | Yes | No | No | Yes | Yes | Yes | PY | Yes | Yes | No | Yes | Yes | Yes | Yes | No | Yes | Critically low |
| Hou Z, 2017 | Yes | No | No | Yes | Yes | Yes | PY | Yes | Yes | No | Yes | Yes | Yes | Yes | Yes | Yes | Critically low |
| Hsieh PC, 2019 | Yes | No | No | Yes | Yes | Yes | PY | Yes | Yes | No | Yes | Yes | Yes | Yes | Yes | Yes | Critically low |
| Hu CQ, 2016 | Yes | No | No | Yes | Yes | Yes | PY | Yes | Yes | No | Yes | Yes | Yes | Yes | Yes | Yes | Critically low |
| Hu H, 2019 | Yes | No | No | PY | Yes | Yes | PY | Yes | Yes | No | Yes | Yes | Yes | Yes | No | Yes | Critically low |
| Hu HT, 2017 | Yes | No | No | Yes | Yes | Yes | PY | Yes | Yes | No | Yes | Yes | Yes | Yes | Yes | Yes | Critically low |
| Hu XY, 2014 | Yes | Yes | Yes | Yes | Yes | Yes | No | Yes | No | No | NM | NM | No | No | NM | No | Critically low |
| Huang CW, 2020 | Yes | No | No | Yes | Yes | Yes | PY | Yes | Yes | No | Yes | Yes | Yes | Yes | Yes | Yes | Critically low |
| Huang F, 2019 | Yes | Yes | No | Yes | Yes | Yes | PY | Yes | Yes | No | Yes | Yes | Yes | Yes | Yes | Yes | Low |
| Huang J,2020 | Yes | No | No | Yes | Yes | Yes | PY | Yes | Yes | No | Yes | Yes | Yes | Yes | Yes | Yes | Critically low |
| Huang JJ, 2021 | Yes | Yes | No | Yes | Yes | Yes | PY | Yes | Yes | No | NM | NM | No | No | NM | Yes | Critically low |
| Huang, KY2020 | Yes | Yes | No | Yes | Yes | Yes | PY | Yes | Yes | No | Yes | Yes | Yes | Yes | No | Yes | Critically low |
| Huang Q, 2019 | Yes | No | No | Yes | Yes | Yes | PY | Yes | Yes | No | Yes | Yes | Yes | Yes | Yes | Yes | Critically low |
| Huang W, 2009 | Yes | No | No | Yes | Yes | Yes | No | Yes | Yes | No | NM | NM | Yes | Yes | No | Yes | Critically low |
| Huang J,2021 | Yes | No | No | Yes | Yes | Yes | PY | Yes | Yes | No | Yes | Yes | Yes | Yes | Yes | Yes | Critically low |
| Huh JH, 2021 | Yes | Yes | No | Yes | Yes | Yes | PY | Yes | Yes | No | NM | NM | No | No | NM | Yes | Critically low |
| Hwang MS, 2020 | Yes | Yes | No | Yes | Yes | Yes | PY | Yes | Yes | No | Yes | No | Yes | No | No | Yes | Critically low |
| Jan AL,2017 | Yes | No | No | Yes | Yes | Yes | PY | Yes | Yes | No | Yes | No | Yes | Yes | No | Yes | Critically low |
| Jang A, 2020 | Yes | No | No | PY | Yes | Yes | PY | Yes | Yes | No | Yes | Yes | Yes | Yes | No | Yes | Critically low |
| Jang S, 2020 | Yes | Yes | No | Yes | Yes | Yes | PY | Yes | Yes | No | NM | NM | Yes | No | NM | Yes | Critically low |
| Jang S, 2020 | Yes | Yes | No | Yes | Yes | Yes | PY | Yes | Yes | No | Yes | Yes | Yes | Yes | No | Yes | Critically low |
| Jedel E, 2005 | Yes | No | No | PY | No | No | No | Yes | NRSI | No | NM | NM | Yes | Yes | NM | No | Critically low |
| Jerng UM, 2014 | Yes | Yes | No | Yes | Yes | Yes | PY | Yes | Yes | No | Yes | Yes | Yes | Yes | No | Yes | Critically low |
| Ji M, 2015 | Yes | No | No | PY | Yes | Yes | PY | Yes | Yes | No | Yes | Yes | Yes | Yes | Yes | Yes | Critically low |
| Jia W, 2020 | Yes | Yes | No | Yes | Yes | Yes | PY | Yes | Yes | No | Yes | No | Yes | No | Yes | Yes | Low |
| Jiang YB, 2015 | Yes | No | No | PY | Yes | Yes | PY | Yes | Yes | No | Yes | No | Yes | Yes | Yes | Yes | Critically low |
| Jin Y, 2020 | Yes | Yes | No | PY | Yes | Yes | PY | Yes | Yes | No | Yes | Yes | Yes | No | No | Yes | Critically low |
| Jo J, 2016 | Yes | No | No | PY | Yes | Yes | PY | Yes | Yes | No | Yes | No | Yes | No | No | Yes | Critically low |
| Jo J, 2014 | Yes | No | No | Yes | Yes | Yes | PY | Yes | Yes | No | Yes | Yes | Yes | Yes | Yes | Yes | Critically low |
| Jo J, 2017 | Yes | Yes | No | Yes | Yes | Yes | PY | Yes | Yes | No | Yes | Yes | Yes | Yes | No | Yes | Critically low |
| Ju ZY, 2017 | Yes | Yes | No | Yes | Yes | Yes | Yes | Yes | Yes | Yes | Yes | Yes | Yes | Yes | Yes | Yes | High |
| Jung A, 2011 | Yes | No | No | Yes | Yes | Yes | PY | Yes | Yes | No | Yes | Yes | Yes | Yes | Yes | Yes | Critically low |
| Kim H, 2019 | Yes | No | No | Yes | Yes | Yes | PY | Yes | Yes | No | Yes | Yes | Yes | No | No | Yes | Critically low |
| Kim JI, 2012 | Yes | No | No | Yes | Yes | Yes | Yes | Yes | Yes | No | NM | NM | Yes | No | NM | Yes | Critically low |
| Kim JI, 2012 | Yes | No | No | PY | Yes | Yes | PY | Yes | Yes | No | Yes | Yes | Yes | No | No | Yes | Critically low |
| Kim KH, 2013 | Yes | No | No | Yes | Yes | Yes | PY | Yes | Yes | No | Yes | No | Yes | Yes | No | Yes | Critically low |
| Kim KH, 2012 | Yes | No | No | Yes | Yes | Yes | PY | Yes | Yes | No | NM | NM | Yes | No | NM | Yes | Critically low |
| Kim KH, 2010 | Yes | No | No | PY | No | Yes | No | Yes | Yes | No | NM | NM | Yes | No | NM | No | Critically low |
| Kim KH, 2018 | Yes | Yes | No | Yes | Yes | Yes | Yes | Yes | Yes | Yes | Yes | Yes | Yes | Yes | Yes | Yes | High |
| Kim KH,2016 | Yes | Yes | No | Yes | Yes | Yes | Yes | Yes | Yes | No | Yes | Yes | Yes | Yes | Yes | Yes | Moderate |
| Kim KN, 2015 | Yes | No | No | PY | Yes | Yes | PY | Yes | Yes | No | Yes | No | Yes | No | No | Yes | Critically low |
| Kim SH, 2019 | Yes | No | No | Yes | Yes | Yes | PY | Yes | Yes | No | Yes | Yes | Yes | Yes | Yes | Yes | Critically low |
| Kim SY, 2011 | Yes | No | No | PY | Yes | Yes | PY | Yes | Yes | No | Yes | No | Yes | No | No | Yes | Critically low |
| Kim SY, 2018 | Yes | No | No | PY | Yes | Yes | PY | Yes | Yes | No | Yes | No | Yes | No | Yes | Yes | Critically low |
| Kim TH, 2014 | Yes | Yes | No | Yes | Yes | Yes | Yes | Yes | Yes | No | Yes | Yes | Yes | Yes | Yes | Yes | Moderate |
| Kim YD, 2013 | Yes | No | No | PY | Yes | Yes | PY | Yes | Yes | No | Yes | No | Yes | No | No | Yes | Critically low |
| Ko GWY, 2019 | Yes | No | No | Yes | Yes | Yes | PY | Yes | Yes | No | NM | NM | Yes | No | NM | Yes | Critically low |
| Ko HF, 2021 | Yes | No | No | Yes | Yes | Yes | PY | Yes | Yes | No | Yes | No | Yes | Yes | Yes | Yes | Critically low |
| Koog YH, 2014 | Yes | No | No | Yes | No | No | PY | No | NRSI | No | Yes | Yes | Yes | Yes | Yes | Yes | Critically low |
| Kuang X, 2021 | Yes | Yes | No | Yes | Yes | Yes | PY | Yes | Yes | No | Yes | Yes | Yes | Yes | Yes | Yes | Low |
| Kwon CY, 2021 | Yes | No | No | PY | Yes | Yes | PY | Yes | Yes | No | NM | NM | Yes | Yes | NM | Yes | Critically low |
| Kwon CY, 2020 | Yes | Yes | No | PY | Yes | Yes | PY | Yes | Yes | No | NM | NM | No | No | NM | Yes | Critically low |
| Kwon CY, 2018 | Yes | Yes | No | Yes | Yes | Yes | PY | Yes | Yes | No | Yes | Yes | Yes | Yes | Yes | Yes | Low |
| La Touche R, 2019 | Yes | No | No | Yes | Yes | Yes | PY | Yes | Yes | No | Yes | No | Yes | No | No | Yes | Critically low |
| Lai XH, 2020 | Yes | Yes | No | Yes | Yes | Yes | PY | Yes | Yes | No | Yes | Yes | Yes | Yes | Yes | Yes | Low |
| Lam YC, 2008 | Yes | No | No | PY | No | No | PY | Yes | Yes | No | NM | NM | Yes | No | NM | No | Critically low |
| Lan J, 2020 | Yes | No | No | Yes | Yes | Yes | PY | Yes | Yes | No | Yes | Yes | Yes | Yes | Yes | Yes | Critically low |
| Lan L, 2014 | Yes | Yes | No | Yes | Yes | Yes | Yes | Yes | Yes | No | NM | NM | Yes | Yes | NM | Yes | Critically low |
| Langhorst, J, 2009 | Yes | No | No | Yes | Yes | Yes | PY | Yes | Yes | No | Yes | Yes | Yes | Yes | Yes | Yes | Critically low |
| Lee A, 2014 | Yes | Yes | No | Yes | Yes | Yes | Yes | Yes | Yes | Yes | Yes | Yes | Yes | Yes | Yes | Yes | High |
| Lee B, 2018 | Yes | Yes | No | Yes | Yes | Yes | PY | Yes | Yes | No | Yes | No | Yes | Yes | No | Yes | Critically low |
| Lee D, 2018 | Yes | No | No | PY | Yes | Yes | PY | Yes | Yes | No | NM | NM | Yes | Yes | NM | Yes | Critically low |
| Lee H, 2003 | Yes | No | No | Yes | No | No | PY | Yes | NRSI | No | NM | NM | No | No | NM | No | Critically low |
| Lee H, 2004 | Yes | No | No | PY | No | No | PY | Yes | Yes | No | NM | NM | Yes | Yes | NM | Yes | Critically low |
| Lee H, 2009 | Yes | No | No | PY | Yes | Yes | PY | Yes | Yes | No | Yes | Yes | Yes | Yes | No | Yes | Critically low |
| Lee H, 2016 | Yes | No | No | Yes | Yes | Yes | Yes | Yes | Yes | No | NM | NM | Yes | Yes | NM | Yes | Critically low |
| Lee H, 2013 | Yes | No | No | Yes | Yes | Yes | PY | Yes | Yes | No | Yes | Yes | Yes | Yes | No | Yes | Critically low |
| Lee H, 2004 | Yes | No | No | Yes | Yes | Yes | Yes | Yes | PY | No | NM | NM | Yes | No | NM | Yes | Critically low |
| Lee HS, 2013 | Yes | No | No | PY | Yes | Yes | PY | Yes | Yes | Yes | Yes | No | Yes | Yes | Yes | Yes | Critically low |
| Lee JA, 2014 | Yes | Yes | No | Yes | Yes | Yes | PY | Yes | Yes | No | NM | NM | Yes | No | NM | Yes | Critically low |
| Lee JH, 2016 | Yes | No | No | PY | No | No | PY | Yes | Yes | No | Yes | Yes | Yes | Yes | No | Yes | Critically low |
| Lee JW, 2020 | Yes | No | No | PY | Yes | Yes | PY | Yes | No | No | NM | NM | No | No | NM | Yes | Critically low |
| Lee MS, 2011 | Yes | No | No | Yes | Yes | Yes | PY | Yes | Yes | No | NM | NM | Yes | No | NM | Yes | Critically low |
| Lee MS, 2008 | Yes | No | No | Yes | Yes | Yes | PY | Yes | PY | No | Yes | Yes | Yes | No | Yes | Yes | Critically low |
| Lee MS, 2009 | Yes | No | No | PY | Yes | Yes | No | Yes | No | No | NM | NM | Yes | No | NM | Yes | Critically low |
| Lee MS, 2009 | Yes | No | No | Yes | Yes | Yes | PY | Yes | PY | No | Yes | Yes | No | Yes | Yes | Yes | Critically low |
| Lee MS, 2008 | Yes | No | No | Yes | Yes | Yes | PY | Yes | PY | No | Yes | Yes | Yes | Yes | Yes | No | Critically low |
| Lee MS, 2011 | Yes | No | No | Yes | No | Yes | PY | Yes | Yes | No | Yes | Yes | Yes | Yes | Yes | Yes | Critically low |
| Lee MS, 2008 | Yes | No | No | Yes | Yes | Yes | PY | Yes | Yes | No | Yes | Yes | Yes | Yes | No | Yes | Critically low |
| Lee, MS, 2008 | Yes | No | No | Yes | Yes | Yes | PY | Yes | PY | No | Yes | Yes | Yes | No | No | Yes | Critically low |
| Lee MS, 2009 | Yes | No | No | Yes | Yes | Yes | PY | Yes | PY | No | NM | NM | Yes | No | NM | Yes | Critically low |
| Lee MS, 2008 | Yes | No | No | Yes | Yes | Yes | PY | Yes | PY | No | Yes | No | Yes | Yes | No | Yes | Critically low |
| Lee MS, 2009 | Yes | No | No | Yes | Yes | Yes | PY | Yes | Yes | No | Yes | Yes | Yes | Yes | No | Yes | Critically low |
| Lee MS, 2008 | Yes | No | No | Yes | Yes | Yes | PY | Yes | PY | No | Yes | No | Yes | No | No | Yes | Critically low |
| Lee SH, 2016 | Yes | No | No | Yes | No | Yes | PY | Yes | Yes | No | Yes | Yes | Yes | Yes | Yes | Yes | Critically low |
| Lee SH, 2016 | Yes | No | No | Yes | No | Yes | PY | Yes | Yes | No | Yes | Yes | Yes | Yes | Yes | Yes | Critically low |
| Lee SJ, 2012 | Yes | No | No | Yes | Yes | Yes | PY | Yes | Yes | No | Yes | Yes | Yes | Yes | Yes | Yes | Critically low |
| Lew J, 2021 | Yes | Yes | No | Yes | Yes | No | Yes | Yes | Yes | No | Yes | No | Yes | Yes | No | Yes | Low |
| Li D, 2019 | Yes | No | No | Yes | Yes | Yes | PY | Yes | Yes | No | Yes | No | Yes | Yes | No | Yes | Critically low |
| Li DZ, 2014 | Yes | No | No | PY | Yes | Yes | PY | Yes | Yes | No | Yes | Yes | Yes | Yes | Yes | Yes | Critically low |
| Li HJ, 2021 | Yes | No | No | Yes | Yes | Yes | PY | Yes | Yes | No | Yes | Yes | Yes | Yes | No | Yes | Critically low |
| Li, JL, 2016 | Yes | No | No | Yes | No | Yes | PY | Yes | PY | No | Yes | Yes | Yes | Yes | No | Yes | Critically low |
| Li JL, 2021 | Yes | No | No | Yes | Yes | Yes | PY | Yes | Yes | No | Yes | Yes | Yes | Yes | Yes | Yes | Critically low |
| Li K, 2019 | Yes | Yes | No | Yes | Yes | Yes | PY | Yes | Yes | No | NM | NM | Yes | Yes | No | Yes | Critically low |
| Li L, 2014 | Yes | No | No | PY | Yes | Yes | PY | Yes | Yes | No | Yes | No | Yes | No | No | Yes | Critically low |
| Li LX, 2015 | Yes | No | No | Yes | Yes | Yes | PY | Yes | Yes | No | Yes | Yes | Yes | Yes | Yes | Yes | Critically low |
| Li M, 2021 | Yes | No | No | Yes | Yes | Yes | PY | Yes | Yes | No | Yes | Yes | Yes | Yes | Yes | Yes | Critically low |
| Li P, 2015 | Yes | No | No | Yes | Yes | Yes | PY | Yes | Yes | No | Yes | Yes | Yes | Yes | Yes | Yes | Critically low |
| Li S, 2018 | Yes | Yes | No | Yes | Yes | Yes | PY | Yes | Yes | No | Yes | Yes | Yes | Yes | Yes | Yes | Low |
| Li SQ, 2022 | Yes | Yes | No | PY | Yes | Yes | PY | Yes | Yes | No | Yes | Yes | Yes | Yes | Yes | Yes | Low |
| Li W, 2020 | Yes | Yes | No | Yes | Yes | Yes | PY | Yes | Yes | No | Yes | Yes | Yes | Yes | Yes | Yes | Low |
| Li X, 2020 | Yes | No | No | PY | No | Yes | PY | Yes | Yes | No | Yes | Yes | Yes | Yes | No | Yes | Critically low |
| Li XR, 2014 | Yes | No | No | Yes | Yes | Yes | Yes | Yes | Yes | No | NM | NM | Yes | No | NM | Yes | Critically low |
| Lian WL, 2014 | Yes | No | No | PY | Yes | Yes | PY | Yes | Yes | No | NM | NM | Yes | No | NM | Yes | Critically low |
| Liang S, 2017 | Yes | No | No | Yes | Yes | Yes | PY | Yes | Yes | No | Yes | Yes | Yes | Yes | Yes | Yes | Critically low |
| Lim B, 2006 | Yes | Yes | No | Yes | Yes | Yes | Yes | Yes | Yes | No | NM | NM | Yes | Yes | No | No | Critically low |
| Lim CED, 2019 | Yes | Yes | No | Yes | Yes | Yes | Yes | Yes | Yes | No | Yes | Yes | Yes | Yes | Yes | Yes | Moderate |
| Lin JG, 2011 | Yes | No | No | No | No | No | PY | Yes | Yes | No | NM | NM | Yes | No | NM | No | Critically low |
| Liu AF, 2020 | Yes | Yes | No | Yes | Yes | Yes | PY | Yes | Yes | No | Yes | No | Yes | Yes | Yes | Yes | Low |
| Liu AJ, 2015 | Yes | No | No | Yes | Yes | Yes | PY | Yes | Yes | No | Yes | No | Yes | No | Yes | Yes | Critically low |
| Liu C, 2019 | Yes | No | No | Yes | Yes | Yes | PY | Yes | Yes | No | Yes | Yes | Yes | Yes | No | Yes | Critically low |
| Liu F, 2016 | No | No | No | PY | Yes | Yes | PY | Yes | Yes | No | Yes | Yes | Yes | Yes | No | Yes | Critically low |
| Liu L, 2022 | Yes | No | No | PY | Yes | Yes | PY | Yes | Yes | No | Yes | Yes | Yes | Yes | Yes | Yes | Critically low |
| Liu P, 2021 | Yes | No | No | Yes | Yes | Yes | PY | No | Yes | No | Yes | Yes | Yes | Yes | Yes | Yes | Critically low |
| Liu SN, 2019 | Yes | Yes | No | PY | Yes | Yes | PY | Yes | Yes | No | Yes | Yes | Yes | Yes | Yes | Yes | Low |
| Liu W, 2020 | Yes | No | No | Yes | Yes | Yes | PY | Yes | Yes | No | Yes | Yes | Yes | Yes | Yes | Yes | Critically low |
| Liu XM, 2021 | Yes | No | No | Yes | Yes | Yes | PY | Yes | Yes | No | Yes | Yes | Yes | Yes | Yes | Yes | Critically low |
| Liu XJ, 2020 | Yes | No | No | Yes | Yes | Yes | PY | Yes | Yes | No | Yes | Yes | Yes | Yes | Yes | Yes | Critically low |
| Liu XL, 2015 | Yes | No | No | Yes | Yes | Yes | PY | Yes | Yes | No | Yes | Yes | Yes | Yes | Yes | Yes | Critically low |
| Liu Y, 2022 | Yes | Yes | No | Yes | Yes | Yes | PY | Yes | Yes | No | Yes | Yes | Yes | Yes | No | Yes | Critically low |
| Yuan L, 2019 | Yes | No | No | Yes | Yes | Yes | PY | Yes | Yes | No | Yes | No | No | No | Yes | Yes | Critically low |
| Yun L, 2019 | Yes | No | No | Yes | Yes | Yes | No | Yes | Yes | No | Yes | Yes | Yes | Yes | Yes | Yes | Critically low |
| Liu YH, 2017 | Yes | Yes | No | Yes | Yes | Yes | PY | Yes | Yes | No | Yes | Yes | Yes | Yes | Yes | Yes | Low |
| Llurda-Almuzara L, 2021 | Yes | Yes | No | Yes | Yes | Yes | PY | Yes | Yes | No | Yes | Yes | Yes | Yes | No | Yes | Critically low |
| Long Z, 2022 | Yes | Yes | No | Yes | Yes | Yes | PY | Yes | Yes | No | NM | NM | Yes | Yes | NM | Yes | Critically low |
| Lu H, 2021 | Yes | Yes | No | Yes | Yes | Yes | PY | Yes | Yes | No | Yes | Yes | Yes | Yes | Yes | Yes | Low |
| Lu HL, 2022 | Yes | Yes | No | Yes | Yes | Yes | PY | Yes | Yes | No | Yes | Yes | Yes | Yes | Yes | Yes | Low |
| Lu W, 2009 | Yes | No | No | Yes | Yes | Yes | PY | Yes | Yes | No | Yes | Yes | Yes | Yes | Yes | No | Critically low |
| Lv ZT, 2016 | Yes | No | No | Yes | Yes | Yes | PY | Yes | Yes | No | Yes | Yes | Yes | Yes | Yes | Yes | Critically low |
| Lu ZT, 2016 | Yes | No | No | Yes | Yes | Yes | PY | Yes | Yes | No | Yes | No | Yes | No | Yes | Yes | Critically low |
| Ma H, 2014 | Yes | No | No | Yes | Yes | Yes | PY | Yes | Yes | No | Yes | No | Yes | Yes | Yes | Yes | Critically low |
| Ma R, 2016 | Yes | No | No | No | Yes | Yes | PY | Yes | Yes | No | NM | NM | Yes | Yes | NM | No | Critically low |
| Mak TC, 2017 | Yes | No | No | PY | Yes | Yes | PY | Yes | Yes | No | Yes | Yes | Yes | Yes | Yes | No | Critically low |
| Ziuk K, 2012 | Yes | No | No | PY | No | No | No | PY | Yes | No | NM | NM | No | No | NM | Yes | Critically low |
| Manheimer E, 2010 | Yes | No | No | Yes | Yes | Yes | Yes | Yes | Yes | Yes | Yes | Yes | Yes | Yes | Yes | Yes | Low |
| Manheimer E, 2012 | Yes | Yes | No | Yes | Yes | Yes | Yes | Yes | Yes | Yes | Yes | Yes | Yes | Yes | Yes | Yes | High |
| Manheimer E, 2018 | Yes | Yes | No | Yes | Yes | Yes | Yes | Yes | Yes | Yes | Yes | Yes | Yes | Yes | Yes | Yes | High |
| Manheimer E, 2007 | Yes | No | No | PY | Yes | Yes | PY | PY | Yes | No | Yes | Yes | Yes | Yes | No | Yes | Critically low |
| Manheimer E, 2013 | Yes | Yes | No | Yes | Yes | Yes | PY | Yes | Yes | No | Yes | Yes | Yes | Yes | Yes | Yes | Low |
| Manheimer E, 2012 | Yes | No | No | PY | Yes | Yes | No | PY | Yes | No | Yes | Yes | Yes | Yes | No | Yes | Critically low |
| Manheimer E, 2008 | Yes | No | No | PY | Yes | Yes | PY | Yes | Yes | No | Yes | Yes | Yes | Yes | No | Yes | Critically low |
| Mansu SSY, 2018 | Yes | No | No | PY | No | No | PY | Yes | Yes | No | Yes | Yes | Yes | Yes | No | Yes | Critically low |
| Mao X, 2020 | Yes | No | No | Yes | Yes | Yes | PY | Yes | Yes | No | Yes | Yes | Yes | Yes | Yes | Yes | Critically low |
| Mills EJ, 2005 | Yes | No | No | PY | Yes | Yes | PY | PY | Yes | No | Yes | No | Yes | No | No | Yes | Critically low |
| Moon TW, 2014 | Yes | No | No | Yes | Yes | Yes | PY | Yes | Yes | No | NM | NM | Yes | Yes | NM | Yes | Critically low |
| Murakami M, 2017 | Yes | No | No | PY | Yes | Yes | PY | Yes | Yes | No | Yes | No | Yes | No | No | Yes | Critically low |
| Naguit N, 2021 | Yes | No | No | Yes | Yes | Yes | PY | Yes | Yes | No | NM | NM | No | Yes | NM | Yes | Critically low |
| Namazi N, 2017 | Yes | No | No | Yes | Yes | Yes | PY | Yes | PY | No | NM | NM | No | No | NM | Yes | Critically low |
| Navarro-Santana MJ,  2020 | Yes | Yes | No | Yes | Yes | Yes | PY | Yes | Yes | No | Yes | Yes | Yes | Yes | Yes | Yes | Low |
| Navarro-Santana MJ, 2022 | Yes | Yes | No | Yes | Yes | Yes | PY | Yes | Yes | No | Yes | No | No | No | Yes | Yes | Critically low |
| Navarro-Santana MJ,  2020 | Yes | Yes | No | Yes | Yes | Yes | PY | Yes | Yes | No | Yes | No | No | Yes | No | Yes | Critically low |
| Ni XX, 2020 | Yes | Yes | No | Yes | Yes | Yes | PY | Yes | Yes | No | Yes | Yes | Yes | Yes | Yes | Yes | Low |
| Noh H, 2017 | Yes | No | No | Yes | Yes | Yes | PY | Yes | Yes | No | Yes | Yes | Yes | Yes | Yes | Yes | Critically low |
| O'Sullivan EM, 2020 | Yes | No | No | Yes | Yes | Yes | Yes | Yes | Yes | No | NM | NM | Yes | No | NM | Yes | Critically low |
| Ou L,2021 | Yes | Yes | No | Yes | Yes | Yes | PY | Yes | Yes | No | Yes | Yes | Yes | Yes | Yes | Yes | Low |
| Paley CA, 2011 | Yes | Yes | No | Yes | Yes | Yes | No | Yes | RCT | No | NM | NM | No | No | NM | Yes | Critically low |
| Pan H, 2018 | Yes | No | No | Yes | Yes | Yes | PY | Yes | Yes | No | Yes | Yes | Yes | Yes | Yes | No | Critically low |
| Pan YQ, 2018 | Yes | No | No | Yes | Yes | Yes | PY | Yes | Yes | No | Yes | Yes | Yes | Yes | No | Yes | Critically low |
| Pang B, 2016 | Yes | No | No | Yes | Yes | Yes | PY | Yes | Yes | No | Yes | Yes | Yes | Yes | No | Yes | Critically low |
| Park J, 2013 | Yes | No | No | Yes | Yes | Yes | PY | Yes | Yes | No | Yes | Yes | Yes | Yes | No | Yes | Critically low |
| Park J, 2001 | Yes | No | No | Yes | Yes | Yes | No | Yes | PY | No | NM | NM | No | No | NM | No | Critically low |
| Park J, 2013 | Yes | No | No | Yes | Yes | Yes | PY | Yes | RCT/  NRSI | No | NM | NM | No | No | NM | Yes | Critically low |
| Park JY, 2017 | Yes | No | No | Yes | Yes | Yes | PY | Yes | Yes | No | Yes | No | No | No | No | Yes | Critically low |
| Park KS, 2017 | Yes | No | No | Yes | No | Yes | PY | Yes | Yes | No | Yes | No | Yes | Yes | Yes | Yes | Critically low |
| Park S, 2020 | Yes | Yes | Yes | Yes | Yes | Yes | PY | Yes | Yes | No | Yes | Yes | Yes | Yes | Yes | Yes | Low |
| Park YJ, 2020 | Yes | No | No | Yes | Yes | Yes | PY | Yes | Yes | No | Yes | No | Yes | Yes | Yes | Yes | Critically low |
| Posadzki P, 2013 | Yes | No | Yes | Yes | Yes | Yes | PY | Yes | Yes | No | NM | NM | Yes | No | NM | Yes | Critically low |
| Posadzki P, 2011 | Yes | No | No | Yes | Yes | Yes | No | Yes | PY | No | Yes | No | No | Yes | No | No | Critically low |
| Pourahmadi M,2021 | Yes | Yes | No | Yes | Yes | Yes | Yes | Yes | Yes | No | Yes | Yes | Yes | Yes | Yes | Yes | Moderate |
| Qin X, 2020 | Yes | Yes | No | Yes | Yes | Yes | PY | Yes | Yes | No | Yes | Yes | Yes | Yes | Yes | Yes | Low |
| Qin Z, 2015 | Yes | No | No | Yes | Yes | Yes | PY | Yes | Yes | No | Yes | Yes | Yes | Yes | Yes | Yes | Critically low |
| Qin ZS, 2016 | Yes | Yes | No | Yes | Yes | Yes | PY | Yes | Yes | No | Yes | Yes | Yes | Yes | Yes | Yes | Low |
| Qiu X, 2021 | Yes | Yes | No | Yes | Yes | Yes | PY | Yes | Yes | No | Yes | Yes | Yes | Yes | Yes | Yes | Low |
| Qu F, 2016 | Yes | No | No | Yes | Yes | Yes | PY | Yes | Yes | No | Yes | No | Yes | Yes | No | Yes | Critically low |
| Rahou-El-Bachiri Y,  2020 | Yes | Yes | No | Yes | Yes | Yes | PY | Yes | Yes | No | Yes | Yes | Yes | Yes | No | Yes | Critically low |
| Rathbone J, 2005 | Yes | Yes | No | Yes | Yes | Yes | Yes | Yes | Yes | No | Yes | Yes | Yes | Yes | Yes | Yes | Moderate |
| Roberts J, 2008 | Yes | No | Yes | Yes | No | Yes | Yes | Yes | PY | No | Yes | Yes | Yes | Yes | Yes | Yes | Critically low |
| Seo SY, 2017 | Yes | No | No | Yes | Yes | Yes | Yes | Yes | Yes | No | Yes | Yes | Yes | Yes | Yes | No | Low |
| Shen FJ, 2019 | Yes | No | No | Yes | Yes | Yes | PY | Yes | Yes | No | Yes | No | Yes | Yes | No | No | Critically low |
| Shen L, 2020 | Yes | No | No | Yes | Yes | Yes | PY | Yes | Yes | No | Yes | No | Yes | No | Yes | Yes | Critically low |
| Sheng J, 2019 | Yes | No | No | Yes | Yes | Yes | PY | Yes | Yes | No | Yes | No | Yes | Yes | No | Yes | Critically low |
| Shergis JL, 2016 | Yes | No | No | Yes | No | Yes | PY | Yes | Yes | Yes | Yes | Yes | Yes | Yes | Yes | Yes | Critically low |
| Sim H, 2011 | Yes | No | No | Yes | No | Yes | PY | Yes | Yes | No | Yes | No | Yes | Yes | No | Yes | Critically low |
| Skjeie H, 2018 | Yes | Yes | No | Yes | Yes | Yes | PY | Yes | Yes | No | Yes | Yes | Yes | Yes | Yes | Yes | Low |
| Smith CA, 2010 | Yes | Yes | No | Yes | Yes | Yes | Yes | Yes | Yes | No | Yes | Yes | Yes | Yes | Yes | Yes | Moderate |
| Smith CA, 2010 | Yes | Yes | No | Yes | Yes | Yes | Yes | Yes | Yes | Yes | Yes | Yes | Yes | Yes | Yes | Yes | High |
| Sniezek DP, 2013 | Yes | No | No | Yes | Yes | Yes | PY | Yes | PY | No | NM | NM | No | No | NM | Yes | Critically low |
| Sorbero M, 2015 | Yes | No | No | Yes | Yes | Yes | Yes | Yes | Yes | No | NM | NM | Yes | No | NM | Yes | Critically low |
| Southern C, 2016 | Yes | No | No | Yes | Yes | Yes | PY | Yes | Yes | No | Yes | Yes | Yes | Yes | Yes | Yes | Critically low |
| Stub T, 2011 | Yes | No | Yes | Yes | No | No | PY | Yes | Yes | No | Yes | Yes | Yes | Yes | Yes | Yes | Critically low |
| Su IJ, 2021 | Yes | No | No | Yes | Yes | Yes | PY | Yes | Yes | No | Yes | Yes | Yes | Yes | Yes | Yes | Critically low |
| Su X, 2021 | Yes | No | No | Yes | Yes | Yes | PY | Yes | Yes | No | Yes | Yes | Yes | Yes | Yes | Yes | Critically low |
| Sun Y, 2008 | Yes | No | No | Yes | Yes | Yes | PY | Yes | PY | No | Yes | Yes | Yes | Yes | No | Yes | Critically low |
| Sun Y, 2008 | Yes | No | No | Yes | Yes | Yes | PY | Yes | RCT | No | Yes | Yes | Yes | Yes | Yes | No | Critically low |
| Sung SH, 2021 | Yes | Yes | No | Yes | Yes | Yes | PY | Yes | Yes | No | NM | NM | No | Yes | NM | Yes | Critically low |
| Sung SH, 2018 | Yes | Yes | No | Yes | Yes | Yes | PY | Yes | Yes | No | Yes | No | No | Yes | Yes | Yes | Critically low |
| Sung WS, 2020 | Yes | Yes | No | Yes | Yes | Yes | PY | Yes | Yes | No | Yes | Yes | Yes | Yes | Yes | Yes | Low |
| Tan J, 2021 | Yes | Yes | No | Yes | Yes | Yes | PY | Yes | Yes | No | Yes | Yes | Yes | Yes | Yes | Yes | Low |
| Tang ECH, 2021 | Yes | No | No | Yes | No | No | PY | Yes | Yes | No | Yes | Yes | Yes | Yes | Yes | Yes | Critically low |
| Tang HZ, 2015 | Yes | Yes | No | Yes | Yes | Yes | PY | Yes | Yes | No | Yes | No | Yes | Yes | No | Yes | Critically low |
| Tang S, 2017 | Yes | No | No | Yes | Yes | Yes | PY | Yes | Yes | No | Yes | Yes | Yes | Yes | Yes | Yes | Critically low |
| Thiagarajah A, 2017 | Yes | No | No | PY | No | No | PY | Yes | RCT | No | NM | NM | Yes | No | NM | No | Critically low |
| Tong QY, 2021 | Yes | Yes | No | Yes | Yes | Yes | PY | Yes | Yes | No | Yes | Yes | Yes | Yes | Yes | Yes | Low |
| Trigkilidas D, 2010 | Yes | No | No | PY | No | No | PY | PY | RCT | No | NM | NM | No | No | NM | No | Critically low |
| Trinh K, 2021 | Yes | No | Yes | Yes | Yes | Yes | PY | Yes | Yes | No | NM | NM | Yes | No | NM | Yes | Critically low |
| Trinh K, 2022 | Yes | No | No | Yes | Yes | Yes | PY | Yes | Yes | No | NM | NM | Yes | Yes | NM | Yes | Critically low |
| Trinh KV, 2016 | Yes | Yes | Yes | Yes | Yes | Yes | Yes | Yes | Yes | Yes | Yes | Yes | Yes | Yes | Yes | Yes | High |
| Tu M, 2021 | Yes | Yes | No | Yes | Yes | Yes | PY | Yes | Yes | No | Yes | Yes | Yes | Yes | Yes | Yes | Low |
| Tu Y, 2022 | Yes | Yes | No | Yes | Yes | Yes | PY | Yes | Yes | No | Yes | Yes | Yes | Yes | Yes | Yes | Low |
| Urroz P, 2013 | Yes | No | No | Yes | No | Yes | No | Yes | PY | No | NM | NM | Yes | Yes | NM | Yes | Critically low |
| Valencia-Chulián R,  2020 | Yes | Yes | No | Yes | Yes | Yes | PY | Yes | Yes | No | NM | NM | Yes | Yes | NM | Yes | Critically low |
| Van den Heuvel E,  2016 | Yes | No | No | Yes | Yes | Yes | PY | Yes | Yes | No | Yes | Yes | Yes | Yes | No | Yes | Critically low |
| Van den Noort M, 2018 | Yes | Yes | No | Yes | Yes | Yes | No | PY | RCT/  NRSI | No | NM | NM | Yes | No | NM | Yes | Critically low |
| Von Trott P, 2020 | Yes | No | No | Yes | No | Yes | PY | Yes | Yes | No | Yes | Yes | Yes | Yes | Yes | Yes | Critically low |
| Wang J, 2018 | Yes | No | No | Yes | Yes | Yes | PY | Yes | Yes | No | Yes | Yes | Yes | Yes | Yes | Yes | Critically low |
| Wang J, 2013 | Yes | No | No | Yes | Yes | Yes | PY | Yes | Yes | No | Yes | Yes | Yes | Yes | Yes | Yes | Critically low |
| Wang J, 2016 | Yes | No | No | Yes | Yes | Yes | PY | Yes | Yes | No | Yes | Yes | Yes | Yes | Yes | Yes | Critically low |
| Wang JH, 2019 | Yes | No | No | Yes | Yes | Yes | PY | Yes | Yes | No | Yes | Yes | Yes | Yes | Yes | Yes | Critically low |
| Wang L, 2021 | Yes | No | No | Yes | Yes | Yes | PY | Yes | Yes | No | Yes | Yes | Yes | Yes | Yes | Yes | Critically low |
| Wang L, 2019 | Yes | No | No | Yes | Yes | Yes | Yes | Yes | Yes | No | Yes | Yes | Yes | Yes | Yes | Yes | Low |
| Wang LQ, 2018 | Yes | No | No | Yes | Yes | Yes | PY | Yes | Yes | No | NM | NM | Yes | Yes | NM | Yes | Critically low |
| Wang M, 2015 | Yes | Yes | No | Yes | Yes | Yes | PY | Yes | Yes | No | Yes | Yes | Yes | Yes | Yes | Yes | Low |
| Wang P, 2022 | Yes | Yes | No | Yes | Yes | Yes | PY | Yes | Yes | No | Yes | Yes | Yes | Yes | Yes | Yes | Low |
| Wang R, 2017 | Yes | No | No | Yes | Yes | Yes | PY | Yes | Yes | No | Yes | Yes | Yes | Yes | Yes | Yes | Critically low |
| Wang TT, 2020 | Yes | No | No | Yes | Yes | Yes | PY | Yes | Yes | No | Yes | Yes | Yes | Yes | Yes | Yes | Critically low |
| Wang WH, 2020 | Yes | No | No | Yes | Yes | Yes | PY | Yes | Yes | No | Yes | Yes | Yes | Yes | Yes | Yes | Critically low |
| Wang XM, 2018 | Yes | No | No | Yes | Yes | Yes | PY | Yes | Yes | No | Yes | Yes | No | Yes | Yes | Yes | Critically low |
| Wang XP, 2018 | Yes | No | No | Yes | Yes | Yes | PY | Yes | Yes | No | Yes | Yes | Yes | Yes | Yes | Yes | Critically low |
| Wang Y, 2018 | Yes | Yes | No | Yes | Yes | Yes | Yes | Yes | Yes | No | Yes | Yes | Yes | Yes | No | Yes | Low |
| Wang Y, 2012 | Yes | No | No | Yes | Yes | Yes | PY | Yes | Yes | No | Yes | No | Yes | Yes | Yes | Yes | Critically low |
| Wang Y, 2013 | Yes | Yes | No | Yes | Yes | Yes | Yes | Yes | Yes | No | Yes | Yes | Yes | Yes | Yes | Yes | Moderate |
| Wei X, 2019 | Yes | No | No | Yes | Yes | Yes | PY | Yes | Yes | No | Yes | Yes | Yes | Yes | Yes | Yes | Critically low |
| Wen X, 2021 | Yes | Yes | No | Yes | Yes | Yes | PY | Yes | Yes | No | Yes | Yes | Yes | Yes | No | Yes | Critically low |
| Wong ISY, 2012 | Yes | No | No | Yes | Yes | Yes | PY | Yes | Yes | No | NM | NM | Yes | No | NM | Yes | Critically low |
| Wong V, 2012 | Yes | Yes | No | Yes | Yes | Yes | Yes | Yes | Yes | No | NM | NM | Yes | Yes | NM | Yes | Critically low |
| Woo HL, 2018 | Yes | Yes | No | Yes | Yes | Yes | PY | Yes | Yes | No | Yes | Yes | Yes | Yes | Yes | Yes | Low |
| Wu HM, 2009 | Yes | Yes | No | Yes | Yes | Yes | Yes | Yes | Yes | No | Yes | Yes | Yes | Yes | Yes | Yes | Moderate |
| Wu IX, 2019 | Yes | Yes | No | Yes | Yes | Yes | Yes | Yes | Yes | No | NM | NM | Yes | No | NM | Yes | Critically low |
| Wu LQ, 2019 | Yes | No | No | Yes | Yes | Yes | PY | Yes | Yes | No | Yes | Yes | Yes | Yes | Yes | Yes | Critically low |
| Xiang A, 2017 | Yes | Yes | No | Yes | Yes | Yes | PY | Yes | Yes | No | Yes | Yes | Yes | Yes | No | Yes | Critically low |
| Xiang Y, 2017 | Yes | PY | No | Yes | Yes | Yes | PY | Yes | Yes | No | Yes | Yes | Yes | Yes | Yes | Yes | Low |
| Xiao X, 2020 | Yes | No | No | Yes | Yes | Yes | PY | Yes | Yes | No | Yes | Yes | Yes | Yes | Yes | Yes | Critically low |
| Xie G, 2020 | Yes | Yes | No | Yes | Yes | Yes | PY | Yes | Yes | No | Yes | No | Yes | Yes | Yes | Yes | Low |
| Xing M, 2019 | Yes | Yes | No | Yes | Yes | Yes | PY | Yes | Yes | No | Yes | Yes | Yes | Yes | No | Yes | Critically low |
| Xiong J, 2021 | Yes | Yes | No | Yes | No | No | PY | Yes | PY | No | Yes | Yes | Yes | Yes | Yes | Yes | Low |
| Xiong W, 2016 | Yes | No | No | Yes | Yes | Yes | PY | Yes | Yes | No | Yes | Yes | Yes | Yes | No | Yes | Critically low |
| Xu G, 2021 | Yes | Yes | No | Yes | Yes | Yes | PY | Yes | Yes | No | Yes | Yes | Yes | Yes | No | Yes | Critically low |
| Xuan Y, 2021 | Yes | Yes | No | Yes | Yes | Yes | PY | Yes | Yes | No | Yes | Yes | Yes | Yes | Yes | Yes | Low |
| Xuan YC, 2020 | Yes | No | No | Yes | Yes | Yes | PY | Yes | Yes | No | Yes | Yes | Yes | Yes | Yes | Yes | Critically low |
| Xue P, 2016 | Yes | No | No | Yes | Yes | Yes | PY | Yes | Yes | No | Yes | No | Yes | No | No | Yes | Critically low |
| Yan B, 2020 | Yes | No | No | Yes | Yes | Yes | PY | Yes | PY | No | Yes | Yes | Yes | Yes | No | Yes | Critically low |
| Yang B, 2013 | Yes | No | No | Yes | Yes | Yes | PY | Yes | Yes | No | Yes | Yes | Yes | Yes | Yes | Yes | Critically low |
| Yang C, 2018 | Yes | No | No | Yes | Yes | Yes | PY | Yes | Yes | No | Yes | Yes | Yes | Yes | Yes | Yes | Critically low |
| Yang J, 2021 | Yes | No | No | Yes | No | No | PY | Yes | Yes | No | Yes | Yes | Yes | Yes | Yes | Yes | Critically low |
| Yang J, 2018 | Yes | Yes | No | Yes | Yes | Yes | Yes | Yes | Yes | Yes | Yes | Yes | Yes | Yes | Yes | Yes | High |
| Yang J, 2020 | Yes | No | No | Yes | Yes | Yes | PY | Yes | Yes | No | Yes | No | Yes | No | Yes | No | Critically low |
| Yang L, 2015 | Yes | No | No | Yes | Yes | Yes | PY | Yes | Yes | No | Yes | Yes | Yes | Yes | No | Yes | Critically low |
| Yang M, 2020 | Yes | Yes | No | Yes | Yes | Yes | PY | Yes | Yes | No | Yes | Yes | Yes | Yes | No | Yes | Critically low |
| Yang MX, 2013 | Yes | No | No | Yes | Yes | Yes | PY | Yes | Yes | No | Yes | Yes | Yes | Yes | No | Yes | Critically low |
| Yang T, 2020 | Yes | Yes | No | Yes | Yes | Yes | PY | Yes | Yes | No | Yes | No | Yes | Yes | Yes | No | Low |
| Yang XY, 2021 | Yes | Yes | No | Yes | Yes | Yes | PY | Yes | Yes | No | Yes | Yes | Yes | Yes | Yes | Yes | Low |
| Yang Y, 2020 | Yes | No | No | Yes | Yes | Yes | PY | Yes | Yes | No | Yes | Yes | Yes | Yes | No | Yes | Critically low |
| Yao JP, 2019 | Yes | No | No | Yes | Yes | Yes | PY | Yes | Yes | No | Yes | Yes | Yes | Yes | Yes | Yes | Critically low |
| Yao Q, 2016 | Yes | Yes | No | Yes | Yes | Yes | PY | Yes | Yes | No | Yes | Yes | Yes | Yes | No | Yes | Critically low |
| Ye JY, 2021 | Yes | No | No | Yes | Yes | Yes | PY | Yes | Yes | No | Yes | Yes | Yes | Yes | No | Yes | Critically low |
| Ye Q, 2017 | Yes | No | No | Yes | Yes | Yes | PY | Yes | Yes | Yes | Yes | Yes | Yes | Yes | Yes | Yes | Critically low |
| Yeung WF, 2009 | Yes | No | No | Yes | Yes | Yes | PY | Yes | Yes | No | NM | NM | No | No | NM | No | Critically low |
| Yi HM, 2020 | Yes | Yes | No | Yes | Yes | Yes | PY | Yes | Yes | No | Yes | Yes | Yes | Yes | No | Yes | Critically low |
| Yin ZH, 2020 | Yes | Yes | No | Yes | Yes | Yes | Yes | Yes | Yes | No | Yes | Yes | Yes | Yes | Yes | Yes | Moderate |
| You F, 2019 | Yes | No | No | Yes | Yes | Yes | PY | Yes | Yes | No | NM | NM | Yes | Yes | NM | Yes | Critically low |
| You YN, 2018 | Yes | No | No | Yes | Yes | Yes | PY | Yes | Yes | No | Yes | Yes | Yes | Yes | No | Yes | Critically low |
| Yu C, 2015 | Yes | No | No | Yes | Yes | Yes | PY | Yes | Yes | No | Yes | Yes | Yes | Yes | Yes | Yes | Critically low |
| Yu CC, 2017 | Yes | No | No | Yes | Yes | Yes | PY | Yes | Yes | No | Yes | Yes | Yes | Yes | No | Yes | Critically low |
| Yu J, 2016 | Yes | No | No | Yes | Yes | Yes | PY | Yes | Yes | No | Yes | Yes | Yes | Yes | No | Yes | Critically low |
| Yu S, 2020 | Yes | No | No | Yes | Yes | Yes | PY | Yes | Yes | No | Yes | No | Yes | Yes | No | Yes | Critically low |
| Yuan J, 2008 | Yes | No | No | PY | Yes | Yes | PY | Yes | Yes | No | Yes | Yes | Yes | No | No | Yes | Critically low |
| Yuan QL, 2016 | Yes | Yes | No | Yes | Yes | Yes | PY | Yes | Yes | No | Yes | Yes | Yes | No | Yes | Yes | Low |
| Yuan XX, 2017 | Yes | No | No | Yes | Yes | Yes | PY | Yes | Yes | No | Yes | Yes | Yes | Yes | Yes | Yes | Critically low |
| Yue J, 2016 | Yes | Yes | No | Yes | Yes | Yes | PY | Yes | Yes | No | Yes | Yes | Yes | Yes | No | Yes | Critically low |
| Yun JM, 2020 | Yes | Yes | No | Yes | Yes | Yes | PY | Yes | Yes | No | Yes | Yes | Yes | Yes | No | Yes | Critically low |
| Zeng YC, 2014 | Yes | No | No | Yes | Yes | Yes | PY | Yes | Yes | No | Yes | No | Yes | Yes | No | Yes | Critically low |
| Zhan J, 2017 | Yes | Yes | No | Yes | Yes | Yes | PY | Yes | Yes | No | Yes | Yes | Yes | Yes | Yes | Yes | Low |
| Zhan J, 2021 | Yes | No | No | Yes | Yes | Yes | PY | Yes | Yes | No | Yes | Yes | Yes | Yes | Yes | Yes | Critically low |
| Zhang BL, 2019 | Yes | Yes | No | Yes | Yes | Yes | PY | Yes | Yes | No | Yes | Yes | Yes | Yes | Yes | Yes | Low |
| Zhang GC, 2012 | Yes | No | No | PY | Yes | Yes | No | Yes | Yes | No | Yes | Yes | Yes | Yes | Yes | Yes | Critically low |
| Zhang J, 2019 | Yes | Yes | No | Yes | Yes | Yes | PY | Yes | Yes | No | Yes | Yes | Yes | Yes | Yes | Yes | Low |
| Zhang J, 2014 | Yes | No | No | Yes | Yes | Yes | PY | Yes | Yes | No | Yes | Yes | Yes | Yes | Yes | Yes | Critically low |
| Zhang JH, 2012 | Yes | No | No | Yes | Yes | Yes | PY | Yes | Yes | No | NM | NM | Yes | Yes | NM | Yes | Critically low |
| Zhang K, 2019 | Yes | PY | No | Yes | Yes | Yes | PY | Yes | Yes | No | Yes | Yes | Yes | Yes | No | Yes | Critically low |
| Zhang K, 2018 | Yes | No | No | Yes | Yes | Yes | PY | Yes | Yes | No | Yes | Yes | Yes | Yes | Yes | Yes | Critically low |
| Zhang N, 2020 | Yes | No | No | Yes | Yes | Yes | PY | Yes | Yes | No | Yes | Yes | Yes | Yes | Yes | Yes | Critically low |
| Zhang N, 2019 | Yes | No | No | Yes | Yes | Yes | PY | Yes | No | No | NM | NM | No | No | NM | Yes | Critically low |
| Zhang Q, 2019 | Yes | No | No | Yes | Yes | Yes | PY | Yes | Yes | No | Yes | Yes | Yes | Yes | Yes | Yes | Critically low |
| Zhang RC, 2019 | Yes | No | No | Yes | Yes | Yes | PY | Yes | Yes | No | Yes | Yes | Yes | Yes | Yes | Yes | Critically low |
| Zhang T, 2013 | Yes | No | No | Yes | Yes | Yes | PY | Yes | Yes | No | Yes | Yes | Yes | Yes | No | Yes | Critically low |
| Zhang W, 2017 | Yes | Yes | No | Yes | Yes | Yes | Yes | Yes | Yes | No | Yes | Yes | Yes | Yes | Yes | Yes | Moderate |
| Zhang X, 2019 | Yes | Yes | No | Yes | Yes | Yes | PY | Yes | Yes | No | Yes | Yes | Yes | Yes | Yes | Yes | Low |
| Zhang X, 2018 | Yes | No | No | Yes | Yes | Yes | PY | Yes | Yes | No | Yes | Yes | Yes | Yes | Yes | Yes | Critically low |
| Zhang X, 2018 | Yes | No | No | Yes | Yes | Yes | PY | Yes | Yes | No | No | Yes | Yes | Yes | Yes | Yes | Critically low |
| Zhang XC, 2019 | Yes | Yes | No | Yes | Yes | Yes | Yes | Yes | Yes | No | Yes | Yes | Yes | Yes | Yes | Yes | Moderate |
| Zhang XC, 2015 | Yes | No | No | Yes | Yes | Yes | PY | Yes | Yes | No | Yes | Yes | Yes | Yes | Yes | Yes | Critically low |
| Zhang XW, 2018 | Yes | No | No | Yes | Yes | Yes | PY | Yes | Yes | No | Yes | Yes | Yes | Yes | Yes | Yes | Critically low |
| Zhang XY, 2019 | Yes | Yes | No | Yes | Yes | Yes | PY | Yes | Yes | No | Yes | Yes | Yes | Yes | No | Yes | Critically low |
| Zhang Y, 2018 | Yes | No | No | Yes | Yes | Yes | PY | Yes | Yes | No | Yes | Yes | Yes | Yes | Yes | Yes | Critically low |
| Zhang Y, 2021 | Yes | Yes | No | Yes | Yes | Yes | PY | Yes | Yes | No | Yes | Yes | Yes | Yes | No | Yes | Critically low |
| Zhang Y, 2020 | Yes | No | No | Yes | Yes | Yes | PY | Yes | Yes | No | Yes | Yes | Yes | Yes | No | Yes | Critically low |
| Zhang Z, 2015 | Yes | No | No | Yes | Yes | Yes | Yes | Yes | No | No | No | Yes | Yes | Yes | Yes | No | Critically low |
| Zhao FY, 2021 | Yes | Yes | No | Yes | Yes | Yes | PY | Yes | Yes | No | Yes | Yes | Yes | Yes | Yes | Yes | Low |
| Zhao FY, 2021 | Yes | Yes | No | Yes | Yes | Yes | Yes | Yes | Yes | No | Yes | Yes | Yes | Yes | Yes | Yes | Moderate |
| Zhao L, 2011 | Yes | No | No | PY | Yes | Yes | PY | Yes | Yes | No | Yes | Yes | Yes | No | No | No | Critically low |
| Zhao QY, 2021 | Yes | Yes | No | Yes | Yes | Yes | PY | Yes | Yes | No | Yes | Yes | Yes | Yes | No | Yes | Critically low |
| Zhao XF, 2015 | Yes | No | No | Yes | Yes | Yes | PY | Yes | Yes | No | Yes | Yes | Yes | Yes | No | Yes | Critically low |
| Zhao YW, 2018 | Yes | Yes | No | No | Yes | Yes | PY | Yes | Yes | No | Yes | Yes | Yes | Yes | No | Yes | Critically low |
| Zheng CH, 2012 | Yes | No | No | Yes | Yes | Yes | No | Yes | Yes | No | Yes | Yes | Yes | Yes | Yes | Yes | Critically low |
| Zheng GQ, 2011 | Yes | No | No | Yes | Yes | Yes | No | Yes | No | No | Yes | Yes | Yes | Yes | Yes | Yes | Critically low |
| Zheng H, 2019 | Yes | No | No | Yes | Yes | Yes | PY | Yes | Yes | No | Yes | Yes | Yes | Yes | Yes | Yes | Critically low |
| Zheng RQ, 2021 | Yes | No | No | Yes | Yes | Yes | PY | Yes | Yes | No | Yes | Yes | Yes | Yes | No | Yes | Critically low |
| Zheng XZ, 2021 | Yes | No | No | Yes | Yes | Yes | PY | Yes | Yes | No | Yes | Yes | Yes | Yes | Yes | Yes | Critically low |
| Zhi FY, 2019 | Yes | No | No | Yes | Yes | Yes | PY | Yes | Yes | No | Yes | Yes | Yes | Yes | No | Yes | Critically low |
| Zhong L, 2021 | Yes | Yes | No | Yes | Yes | Yes | PY | Yes | Yes | No | Yes | Yes | Yes | Yes | Yes | Yes | Low |
| Zhong Y, 2018 | Yes | Yes | No | Yes | Yes | Yes | PY | Yes | Yes | No | Yes | Yes | Yes | Yes | Yes | Yes | Low |
| Zhong Y, 2019 | Yes | Yes | No | Yes | Yes | Yes | PY | Yes | Yes | No | Yes | Yes | Yes | Yes | Yes | Yes | Low |
| Zhong YJ, 2020 | Yes | Yes | No | Yes | Yes | Yes | PY | Yes | Yes | No | Yes | Yes | Yes | Yes | No | No | Critically low |
| Zhong YM, 2019 | Yes | Yes | No | Yes | Yes | Yes | PY | Yes | Yes | No | Yes | Yes | Yes | Yes | No | No | Critically low |
| Zhou J, 2015 | Yes | Yes | No | Yes | Yes | Yes | PY | Yes | Yes | No | Yes | Yes | Yes | Yes | No | Yes | Critically low |
| Zhou L, 2020 | Yes | No | No | Yes | Yes | Yes | PY | Yes | Yes | No | Yes | Yes | Yes | Yes | Yes | Yes | Critically low |
| Zhou M, 2009 | Yes | PY | No | Yes | Yes | Yes | No | Yes | No | No | Yes | Yes | Yes | Yes | No | Yes | Critically low |
| Zhou WM, 2016 | Yes | No | No | Yes | Yes | Yes | PY | Yes | Yes | No | Yes | Yes | Yes | Yes | Yes | Yes | Critically low |
| Zhu F, 2021 | Yes | Yes | No | Yes | Yes | Yes | Yes | Yes | Yes | No | Yes | Yes | Yes | Yes | Yes | Yes | Moderate |
| Zhu J, 2016 | Yes | Yes | No | Yes | Yes | Yes | Yes | Yes | Yes | No | Yes | Yes | Yes | Yes | Yes | Yes | Moderate |
| Zhu X, 2011 | Yes | Yes | No | Yes | Yes | Yes | Yes | Yes | Yes | No | Yes | Yes | Yes | Yes | No | No | Low |
| Zhuang L, 2012 | Yes | No | No | Yes | Yes | Yes | Yes | Yes | Yes | No | NM | NM | Yes | Yes | NM | Yes | Critically low |
| Zheng J, 2022 | Yes | No | No | Yes | Yes | Yes | PY | Yes | No | No | Yes | Yes | Yes | Yes | Yes | Yes | Critically low |
| Kai-feng D, 2022 | Yes | No | No | Yes | Yes | Yes | PY | Yes | Yes | No | Yes | Yes | Yes | Yes | Yes | Yes | Critically low |
| Hou Y, 2020 | Yes | Yes | No | Yes | Yes | Yes | PY | Yes | Yes | No | Yes | Yes | Yes | Yes | Yes | Yes | Low |
| Li, LX, 2019 | Yes | No | No | Yes | Yes | Yes | PY | Yes | Yes | No | Yes | Yes | Yes | Yes | Yes | Yes | Critically low |
| Huang T, 2011 | Yes | Yes | No | Yes | Yes | Yes | Yes | Yes | Yes | No | Yes | Yes | Yes | Yes | No | Yes | Low |
| Lee SW, 2017 | Yes | No | No | Yes | Yes | Yes | PY | Yes | Yes | No | Yes | Yes | Yes | Yes | No | Yes | Critically low |
| Chan YY, 2015 | Yes | No | No | Yes | Yes | Yes | PY | Yes | Yes | No | Yes | Yes | Yes | Yes | No | Yes | Critically low |
| Jiang HL, 2020 | Yes | Yes | No | Yes | Yes | Yes | PY | Yes | Yes | No | Yes | Yes | Yes | Yes | Yes | Yes | Low |
| Liu TT, 2009 | Yes | No | No | Yes | Yes | Yes | No | Yes | No | No | Yes | No | Yes | No | No | Yes | Critically low |
| Li Y, 2017 | Yes | No | No | Yes | Yes | Yes | PY | Yes | Yes | No | Yes | Yes | Yes | Yes | Yes | Yes | Critically low |
| Lingling Y, 2018 | Yes | Yes | No | Yes | Yes | Yes | PY | Yes | Yes | No | Yes | Yes | Yes | Yes | No | Yes | Critically low |
| Xie Q, 2020 | Yes | Yes | No | Yes | Yes | Yes | PY | Yes | Yes | No | Yes | No | Yes | Yes | Yes | Yes | Low |
| Wong V, 2013 | Yes | Yes | No | Yes | Yes | Yes | PY | Yes | Yes | No | Yes | Yes | Yes | Yes | Yes | No | Low |
| Wang X, 2021 | Yes | No | No | Yes | Yes | Yes | PY | Yes | Yes | No | Yes | Yes | Yes | Yes | No | Yes | Critically low |
| Wen X, 2021 | Yes | No | No | Yes | Yes | Yes | PY | Yes | Yes | No | Yes | Yes | Yes | Yes | No | No | Critically low |
| Zhang K, 2021 | Yes | No | No | Yes | Yes | Yes | PY | Yes | Yes | No | Yes | Yes | Yes | Yes | Yes | Yes | Critically low |
| Yang J, 2021 | Yes | Yes | No | Yes | Yes | Yes | PY | Yes | Yes | No | Yes | Yes | Yes | Yes | Yes | Yes | Low |
| You J, 2021 | Yes | Yes | No | Yes | No | Yes | PY | Yes | Yes | No | Yes | No | No | No | Yes | Yes | Critically low |
| Zhang J,2020 | Yes | No | No | Yes | No | Yes | PY | Yes | Yes | No | Yes | Yes | No | Yes | Yes | Yes | Critically low |
| Zhang Q,2017 | Yes | Yes | No | Yes | Yes | Yes | PY | Yes | Yes | No | Yes | Yes | Yes | Yes | No | Yes | Critically low |
| Xu M,2018 | Yes | No | No | Yes | Yes | Yes | Yes | Yes | Yes | No | Yes | Yes | Yes | Yes | Yes | Yes | Low |
| wei Z,2104 | Yes | No | No | Yes | Yes | Yes | PY | Yes | Yes | No | Yes | Yes | Yes | Yes | Yes | No | Critically low |
| Zhang Y, 2020 | Yes | Yes | No | Yes | No | Yes | PY | Yes | Yes | No | Yes | Yes | Yes | Yes | Yes | Yes | Low |
| Zhang ZJ,2010 | Yes | No | No | Yes | No | No | PY | Yes | No | No | Yes | Yes | Yes | No | Yes | Yes | Critically low |
| Zheng C,2022 | Yes | Yes | No | Yes | Yes | Yes | PY | Yes | Yes | No | Yes | Yes | Yes | Yes | Yes | Yes | Low |
| Zhou L,2022 | Yes | Yes | No | Yes | Yes | Yes | PY | Yes | Yes | No | Yes | Yes | Yes | Yes | Yes | Yes | Low |
| **Zhu LB, 2014** | Yes | No | No | Yes | Yes | Yes | PY | Yes | PY | No | Yes | Yes | Yes | Yes | No | Yes | Critically low |
| Xu J,2018 | Yes | Yes | No | Yes | Yes | Yes | PY | Yes | Yes | No | Yes | Yes | Yes | Yes | Yes | Yes | Low |
| Xu Y, 2017 | Yes | No | No | Yes | Yes | Yes | PY | Yes | Yes | No | Yes | Yes | Yes | Yes | Yes | Yes | Critically low |
| Yang J, 2021 | Yes | Yes | No | Yes | Yes | Yes | PY | Yes | Yes | No | Yes | Yes | Yes | Yes | Yes | Yes | Low |
| Yang M, 2019 | Yes | Yes | No | Yes | Yes | Yes | PY | Yes | Yes | No | Yes | Yes | Yes | Yes | No | Yes | Critically low |
| Zheng J, 2022 | Yes | No | No | Yes | Yes | Yes | PY | Yes | No | No | Yes | Yes | Yes | Yes | Yes | Yes | Critically low |
| Deng K, 2022 | Yes | No | No | Yes | Yes | Yes | PY | Yes | Yes | No | Yes | Yes | Yes | Yes | Yes | Yes | Critically low |
| Fan SQ, 2021 | Yes | No | No | Yes | Yes | Yes | PY | Yes | PY | No | Yes | Yes | Yes | Yes | No | Yes | Critically low |
| Fei Y, 2019 | Yes | No | No | Yes | Yes | Yes | PY | PY | PY | Yes | Yes | No | Yes | Yes | No | Yes | Critically low |
| Guo J, 2020 | Yes | Yes | No | Yes | Yes | Yes | PY | Yes | Yes | No | Yes | Yes | Yes | Yes | Yes | Yes | Low |
| He C, 2017 | Yes | No | No | Yes | Yes | Yes | PY | Yes | Yes | No | Yes | Yes | Yes | Yes | Yes | Yes | Critically low |
| Hou XB, 2020 | Yes | No | No | Yes | Yes | Yes | PY | Yes | PY | No | Yes | Yes | Yes | Yes | Yes | Yes | Critically low |
| Höxtermann MD, 2021 | Yes | Yes | No | Yes | Yes | Yes | Yes | Yes | Yes | Yes | Yes | Yes | Yes | Yes | Yes | Yes | High |
| Huang JF, 2020 | Yes | Yes | No | Yes | Yes | Yes | Yes | Yes | Yes | Yes | Yes | Yes | Yes | Yes | Yes | Yes | High |
| Wang L, 2022 | Yes | Yes | No | Yes | Yes | Yes | PY | Yes | Yes | Yes | Yes | Yes | Yes | Yes | Yes | Yes | Low |
| Wang L, 2020 | Yes | Yes | No | Yes | Yes | Yes | PY | Yes | Yes | Yes | Yes | Yes | Yes | Yes | Yes | Yes | Low |
| Wang X, 2020 | Yes | Yes | No | Yes | Yes | Yes | PY | Yes | Yes | Yes | Yes | Yes | Yes | Yes | Yes | Yes | Low |
| Wang XF, 2021 | Yes | No | No | Yes | Yes | Yes | PY | Yes | Yes | Yes | Yes | Yes | Yes | Yes | Yes | Yes | Critically low |
| Lan Y, 2015 | Yes | No | No | Yes | Yes | Yes | PY | Yes | Yes | No | Yes | Yes | Yes | Yes | Yes | Yes | Critically low |
| Li P, 2020 | Yes | Yes | No | Yes | Yes | Yes | PY | Yes | Yes | No | Yes | Yes | Yes | Yes | No | Yes | Critically low |
| Li X, 2017 | Yes | Yes | No | Yes | Yes | Yes | PY | Yes | Yes | No | Yes | Yes | Yes | Yes | Yes | Yes | Low |
| Mu J, 2020 | Yes | Yes | No | Yes | Yes | Yes | Yes | Yes | Yes | Yes | Yes | Yes | Yes | Yes | Yes | Yes | High |
| Ou MQ, 2020 | Yes | No | No | Yes | Yes | Yes | PY | Yes | Yes | No | Yes | No | No | Yes | Yes | Yes | Critically low |
| Pei W, 2019 | Yes | Yes | No | Yes | Yes | Yes | PY | Yes | Yes | No | Yes | Yes | Yes | Yes | Yes | Yes | Low |
| Quan K, 2022 | Yes | No | No | Yes | Yes | Yes | PY | Yes | Yes | No | Yes | Yes | Yes | Yes | Yes | Yes | Critically low |
| Smith CA, 2018 | Yes | Yes | No | Yes | Yes | Yes | Yes | Yes | Yes | No | Yes | Yes | Yes | Yes | Yes | Yes | Moderate |
| Smith CA, 2018 | Yes | No | No | Yes | Yes | Yes | Yes | Yes | Yes | No | Yes | Yes | Yes | Yes | Yes | Yes | Low |
| Smith CA, 2016 | Yes | Yes | No | Yes | Yes | Yes | Yes | Yes | Yes | Yes | Yes | Yes | Yes | Yes | Yes | Yes | High |
| Su XT, 2021 | Yes | Yes | No | Yes | Yes | Yes | PY | Yes | Yes | No | Yes | Yes | Yes | Yes | Yes | Yes | Low |
| Tao B, 2015 | Yes | No | No | Yes | Yes | Yes | PY | Yes | Yes | No | Yes | Yes | Yes | Yes | Yes | No | Critically low |
| Xi-yang Wang, 2021 | Yes | Yes | No | Yes | Yes | Yes | PY | No | Yes | No | Yes | Yes | Yes | Yes | Yes | Yes | Low |
| Wang Y, 2020 | Yes | No | No | Yes | Yes | Yes | PY | Yes | Yes | No | Yes | Yes | Yes | No | Yes | No | Critically low |
| Xiao X, 2020 | Yes | Yes | No | Yes | Yes | Yes | PY | Yes | Yes | No | Yes | Yes | Yes | Yes | Yes | Yes | Low |
| Linde K, 2016 | Yes | Yes | No | Yes | Yes | Yes | Yes | Yes | Yes | Yes | Yes | Yes | Yes | Yes | Yes | Yes | High |
| Linde K, 2018 | Yes | Yes | No | Yes | Yes | Yes | Yes | Yes | Yes | Yes | Yes | Yes | Yes | Yes | Yes | Yes | High |
| Linde K, 2010 | Yes | Yes | No | Yes | Yes | Yes | Yes | Yes | Yes | Yes | Yes | Yes | Yes | Yes | Yes | Yes | High |
| Liu B, 2016 | Yes | No | No | Yes | Yes | Yes | Yes | Yes | Yes | Yes | Yes | Yes | Yes | Yes | Yes | Yes | Low |
| Liu F, 2019 | Yes | No | No | Yes | Yes | Yes | Yes | Yes | Yes | Yes | Yes | Yes | Yes | Yes | No | Yes | Critically low |
| Liu F, 2021 | Yes | Yes | No | No | Yes | Yes | Yes | Yes | Yes | Yes | Yes | Yes | Yes | Yes | Yes | Yes | Low |
| Liu R, 2021 | Yes | Yes | No | PY | Yes | Yes | Yes | Yes | Yes | Yes | Yes | Yes | Yes | Yes | Yes | Yes | Low |
| Lu C, 2021 | Yes | Yes | No | PY | Yes | Yes | Yes | Yes | Yes | Yes | Yes | Yes | Yes | Yes | Yes | Yes | Low |
| Lu W, 2016 | Yes | No | No | Yes | Yes | Yes | Yes | Yes | Yes | No | Yes | Yes | Yes | Yes | Yes | No | Low |
| Luo Y, 2020 | Yes | No | No | No | Yes | Yes | Yes | Yes | Yes | Yes | Yes | Yes | Yes | Yes | Yes | Yes | Critically low |
| Manyanga T, 2014 | Yes | Yes | No | Yes | Yes | Yes | Yes | Yes | Yes | Yes | Yes | Yes | Yes | Yes | Yes | Yes | High |
| Armour M,2019 | Yes | No | No | Yes | No | Yes | PY | Yes | Yes | No | Yes | Yes | Yes | Yes | Yes | Yes | Critically low |
| Bae H,2014 | Yes | No | No | Yes | Yes | Yes | PY | Yes | PY | No | Yes | Yes | Yes | Yes | Yes | Yes | Critically low |
| Cao H,2009 | Yes | No | No | Yes | Yes | Yes | PY | Yes | PY | No | Yes | No | Yes | Yes | NM | Yes | Critically low |
| Cao H,2019 | Yes | Yes | No | Yes | Yes | Yes | PY | Yes | No | No | Yes | Yes | Yes | Yes | NM | Yes | Critically low |
| Chen H,2018 | Yes | Yes | No | Yes | Yes | Yes | PY | Yes | Yes | No | Yes | Yes | Yes | Yes | Yes | Yes | Low |
| Jiang Y,2018 | Yes | No | No | Yes | Yes | Yes | PY | Yes | Yes | No | Yes | Yes | Yes | Yes | Yes | Yes | Critically low |
| Kong DZ,2019 | Yes | Yes | No | Yes | Yes | Yes | Yes | Yes | Yes | Yes | Yes | Yes | Yes | Yes | Yes | Yes | High |
| Chan-Young Kwon,2021 | Yes | Yes | No | Yes | Yes | Yes | PY | Yes | Yes | No | Yes | Yes | Yes | Yes | Yes | Yes | Low |

PY = Partial yes; NM= Not conducted meta-analysis; RCT: Randomized controlled trial NRSI: Non-randomized studies of interventions; Q = Question; Q1: Did the research questions and inclusion criteria for the review include the components of PICO? , Q2: Did the report of the review contain an explicit statement that the review methods were established prior to the conduct of the review and did the report justify any significant deviations from the protocol? , Q3: Did the review authors explain their selection of the study designs for inclusion in the review? , Q4: Did the review authors use a comprehensive literature search strategy? , Q5: Did the review authors perform study selection in duplicate? , Q6: Did the review authors perform data extraction in duplicate? , Q7: Did the review authors provide a list of excluded studies and justify the exclusions? , Q8: Did the review authors describe the included studies in adequate detail? , Q9: Did the review authors use a satisfactory technique for assessing the risk of bias (RoB) in individual studies that were included in the review? , Q10: Did the review authors report on the sources of funding for the studies included in the review? , Q11: If meta-analysis was performed, did the review authors use appropriate methods for statistical combination of results? , Q12: If meta-analysis was performed, did the review authors assess the potential impact of RoB in individual studies on the results of the meta-analysis or other evidence synthesis? , Q13: Did the review authors account for RoB in primary studies when interpreting/discussing the results of the review? , Q14: Did the review authors provide a satisfactory explanation for, and discussion of, any heterogeneity observed in the results of the review? ,Q15: If they performed quantitative synthesis did the review authors carry out an adequate investigation of publication bias (small study bias) and discuss its likely impact on the results of the review? , Q16: Did the review authors report any potential sources of conflict of interest, including any funding they received for conducting the review?
